# Transcriptome and lipidome profile of human mesenchymal stem cells with reduced senescence and increased trilineage differentiation ability upon drug treatment

**DOI:** 10.18632/aging.202759

**Published:** 2021-03-26

**Authors:** Yue Chen, Xinglan An, Zengmiao Wang, Shuanghong Guan, Hongyu An, Qingyuan Huang, Haobo Zhang, Lin Liang, Bo Huang, Huiyu Wang, Min Lu, Huan Nie, Jun Wang, Xiangpeng Dai, Xin Lu

**Affiliations:** 1 School of Life Science and Technology, Harbin Institute of Technology, Harbin 150080, Heilongjiang, China; 2 National & Local Joint Engineering Laboratory for Animal Models of Human Diseases, First Hospital, Jilin University, Changchun 130021, China; 3 Department of Chemistry and Biochemistry, University of California, San Diego, La Jolla, CA 92093, USA; 4 State Key Laboratory of Remote Sensing Science, College of Global Change and Earth System Science, Beijing Normal University, Beijing 100875, China; 5 School of Pharmacy, Qiqihar Medical University, Qiqihar 161000, Heilongjiang, China; 6 BeiGene (Beijing) Co., Ltd, Beijing 102206, China

**Keywords:** lipidomics, transcriptomics, hMSCs, aging, drugs

## Abstract

Human Mesenchymal stem cells (hMSCs) are multi-potential cells which are widely used in cell therapy. However, the frequently emerged senescence and decrease of differentiation capabilities limited the broad applications of MSC. Several strategies such as small molecules treatment have been widely studied and used to improve the stem characteristics bypassing the senescence but the exact mechanisms for them to reduce senescence have not been fully studied. In this study, hMSCs were treated by rapamycin, oltipraz, metformin, and vitamin C for the indicated time and these cells were subjected to senescence evaluation and trilineage differentiation. Furthermore, transcriptomics and lipidomics datasets of hMSCs after drug treatment were analyzed to interpret biological pathways responsible for their anti-senescence effects. Although four drugs exhibited significant activities in promoting MSC osteogenic differentiation, metformin is the optimal drug to promote trilineage differentiation. GO terms illustrated that the anti-aging effects of drugs were mainly associated with cellular senescence, mitotic and meiosis process. Biosynthesis of phosphatidylcholines (PC) and phosphatidylethanolamine (PE) were inhibited whereas production of phosphatidylinositols (PIs) and saturated fatty acids (SFA)/ mono-unsaturated fatty acids (MUFA) conversion was activated. Medium free fatty acids (FFA) was increased in hMSCs with different anti-aging phenotypes. Therefore, we established a comprehensive method in assessing drug intervention based on the results of transcriptomics and lipidomics. The method can be used to study different biological phenotypes upon drug intervention in MSC which will extend the clinical application of hMSCs.

## INTRODUCTION

Aging, a time-dependent process, will cause physiological dysfunction. Aging is the primary risk factor for many diseases, including cancer, neurodegenerative diseases, and diabetes. Stem cell exhaustion and cellular senescence are one of the hallmarks that determine physiological phenotype during aging [1]. Human mesenchymal stem cells (hMSCs) are multi-potential cells presented in bone marrow, adipose tissue, placenta, umbilical cord, muscle and many other tissues [2–6]. Under lineage specific medium, hMSCs can undergo differentiation into adipocyte, osteoblast and chondrocytes as well as other cell lineages *in vitro*. Therefore, hMSCs play important roles in regenerative medicine and cell therapy. However, the clinical usage of MSC is limited by the aging during the longer culture *in vitro*. hMSCs aging was usually associated with the change of telomere length, cell density, proliferation potential, trilineage differentiation potential, epigenetics, mitochondrial function and secretome [7]. Therefore, overcoming MSC aging warrants more in-depth studies to explore the strategies to inhibit hMSC senescence.

Metformin (TAME) is the first drug which was approved in clinical trial to delay aging [8], and is also a FDA-approved first-line drug for treating type 2 diabetes mellitus [9]. Agonizing Nrf2 pathway is thought the underlying mechanism for metformin to mediate lifespan elongation [10]. Oltipraz, an antischistosomal agent, was also used to inhibit cancer progression via NRF2 pathway in a rodent model [11, 12]. It was also reported that oltipraz exhibited anti-aging effect by repressing antioxidant NRF2 pathway [13]. Moreover, rapamycin (Sirolimus) was used to coat coronary stents, prevent organ transplant rejection and treat lymphangioleiomyomatosis [14]. It was reported that rapamycin could inhibit activation of T cells and B cells through mTOR inhibition [15], which has been further confirmed by the aggravation of the deficiency of stem cells [16]. Vitamin C is a well-known reductant and has been reported to alleviate aging in the Werner syndrome stem cell model [17]. However, whether they shared similar pathways to exert their roles in anti-aging and the specific mechanisms for suppressing cellular aging in humans remains unclear. Furthermore, the transcriptome and lipidome for these MSC treated by the four drugs was not fully investigated which urged us to perform the below experiments to address these important questions.

In this study, metformin, oltipraz, rapamycin and vitamin C were used to treat Bone Marrow hMSCs (BM-hMSCs). Firstly, differentiation phenotypes of hMSCs after treatment were characterized to illustrate their anti-aging potentials. Transcriptomics and lipidomics analysis was performed on the drug treated cells. Afterwards, GO analysis based on transcriptomics dataset was carried out to anchor the signaling events responsible for senescence and anti-aging activities. Metabolic profiling and pathway analysis were conducted which illustrates a fluctuation of lipids production during drug treatment. Altogether, we have established an overall evaluation system of drug intervention on hMSCs senescence. The system can be used to study different biological phenotypes upon drug intervention in MSC which will extend the clinical application of hMSCs.

## RESULTS

### BM-hMSC subjects to senescence after long time culture *in vitro*

**Figure 1:**
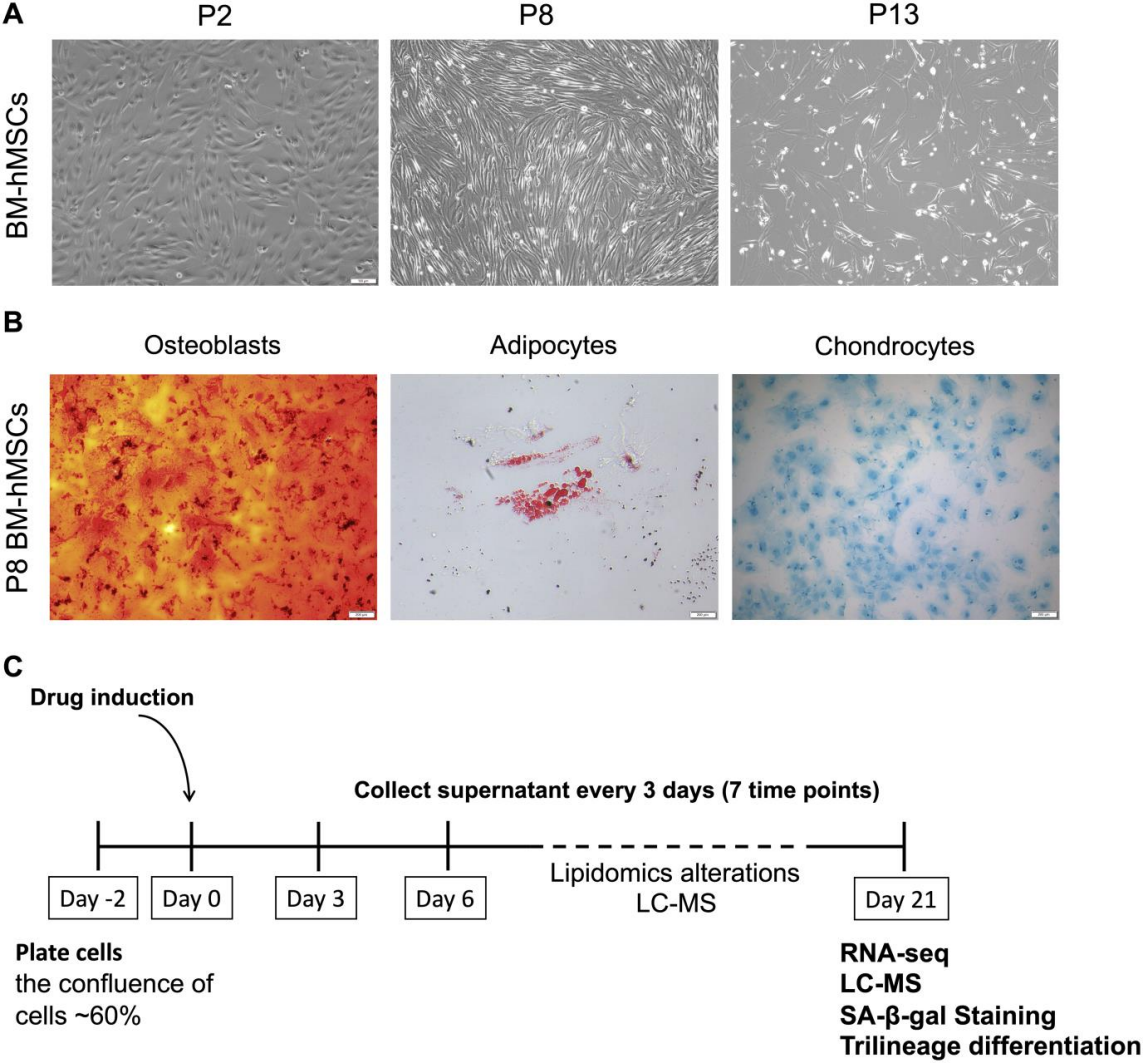
**BM-hMSCs senescence phenotype and drug induction strategy.** (**A**) Morphology of BM-hMSCs at passage 2 (p2), passage 8 (p8) and passage 13 (p13). Scale bar, 100 μm. (**B**) Trilineage differentiation of BM-hMSCs. Scale bar, 100 μm. (**C**) An illustration of the method used for drug treatment of hMSCs and evaluation system.

BM-hMSCs are multipotent stem cells with common characteristics of self-renew and differentiation into different cell types. However, with the passage increasing *in vitro*, cells gradually subject to senescence and will lost the majority of their differentiation capabilities. The status of BM-hMSCs was analyzed at different passage (P2, P8, P13) and the cells condition became worse, proved by the slowing down of cell proliferation, the increase of death and the lengthening of morphology (Figure 1A). Furthermore, the trilineage differentiation abilities of P8 BM-hMSCs was also tested. As shown in Figure 1B, compared with that from our previous studies [18], the differentiation abilities of P8 BM-hMSCs decreased, but can still differentiated to osteoblasts, adipocytes and chondrocytes under induction medium. According to the *in vitro* hMSCs senescence model as reported [18], we further treated BM-hMSCs by four candidate drugs aiming to intervene the senescence *in vitro*. As shown in Figure 1C, cells were plated in 24-well plate with a density of 60 percent and drugs were added to the cells after 48 hours. The supernatant of cells was collected every three days for seven times. By day 21 after drug treatment, cells were collected for RNA-seq, liquid chromatography - mass spectrometry (LC-MS), senescence-associated-β-galactosidase (SA-β-gal) activity analysis and trilineage differentiation assay.

### Drugs treatment reduced the senescence and improved the trilineage differentiation abilities of hMSC

**Figure 2:**
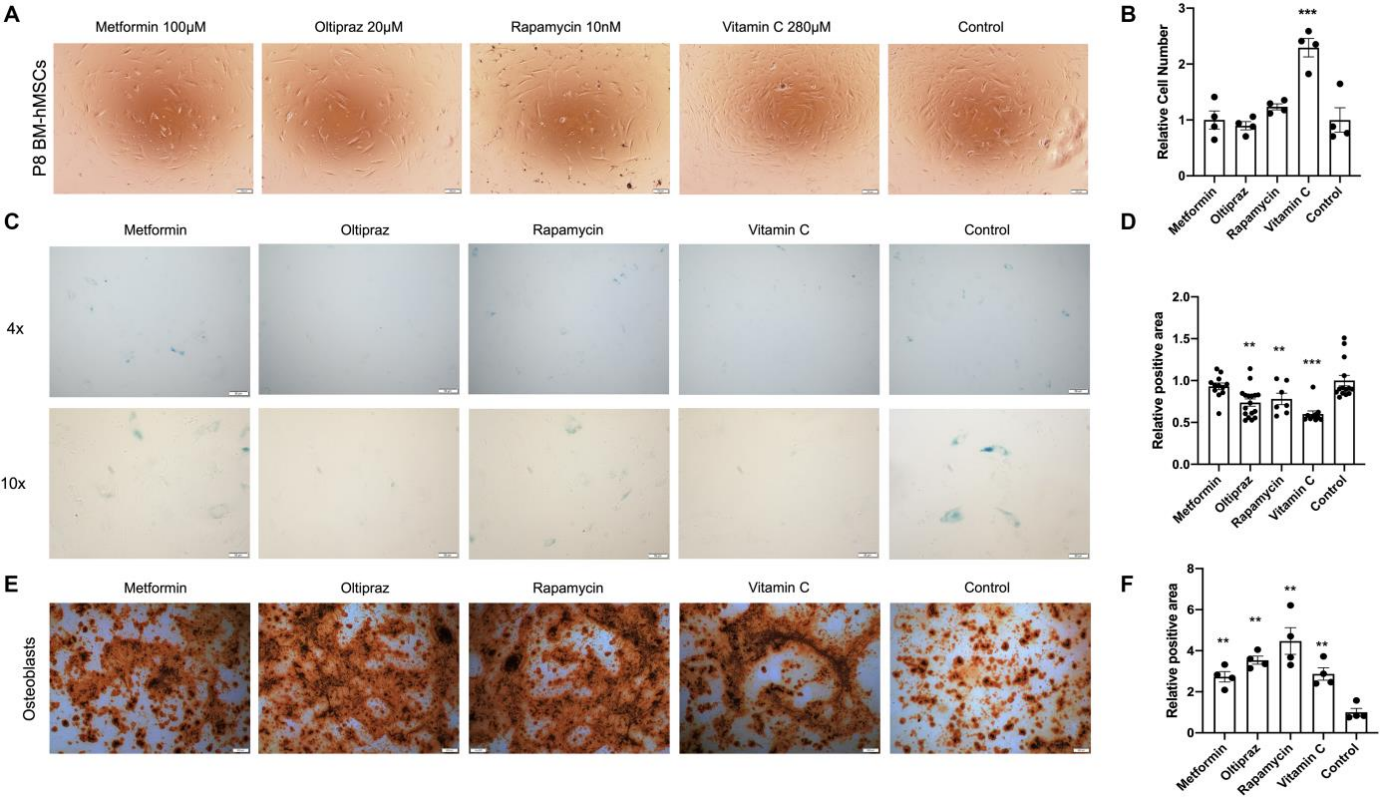
**Senescence alleviation and trilineage differentiation improvement of drug inducted BM-hMSCs**. (**A**) Morphology of BM-hMSCs in day 10 after drug induction. Scale bar, 100 μm. (**C**) SA-β-gal staining of BM-hMSCs inducted with the indicated drugs. (**E**) Oil red O staining on day 25 after transduction to label adipocyte. Scale bar, 200 μm. (**B**), (**D**), and (**F**) ImageJ was used for image analysis. Pictures were quantified by measuring the positive staining area. Data are presented as the mean ± SEM, *n* > 3, ^*^P < 0.05, ^**^P < 0.01, ^***^P < 0.001.

Interestingly, all the candidate drugs play important roles in anti-aging [10, 13, 16, 17], but their effect in combating BM-hMSCs senescence was not investigated. To analyze their effect on hMSC proliferation, P8 BM-hMSCs were treated by metformin (100 μM), oltipraz (20 μM), rapamycin (10 nM) and vitamin C (280 μM) for 10 days. As shown in Figure 2A, except for the Vitamin C, which could dramatically promote cell proliferation, the other three drugs had no significant effect on proliferation of BM-hMSCs (Figure 2B). Moreover, the ability of drugs to alleviate the senescence was assessed by SA-β-gal activity assay and all the four drugs significantly reduced the SA-β-gal activity (*p* < 0.01, Figure 2C) although metformin slightly inhibits SA-β-gal activity (Figure 2D). Altogether, these results indicated that all the candidate drugs could prevent BM-hMSCs from senescence at different degrees.

After confirming the anti-senescence role of the drugs in BM-hMSCs, we further analyzed the effect of drugs on trilineage differentiation abilities of BM-hMSCs. Under osteogenic differentiation medium induction, BM-hMSCs could differentiate into osteoblasts (Figure 2E). However, the four drugs show distinct ability in improving the osteogenic differentiation efficiency of BM-hMSCs. The capability to induce osteogenic differentiation is as follows: rapamycin (^**^p < 0.01) > oltipraz (^**^p < 0.01) > metformin (^**^p < 0.01) > vitamin C (^**^p < 0.01) (Figure 2F). Upon chondrogenic differentiation medium induction, BM-hMSCs differentiated into chondrocytes (Supplementary Figure 1A). Oltipraz, rapamycin, and vitamin C can effectively improve the differentiation potential which was directly proportional to the antagonism of senescence (Supplementary Figure 1B). Interestingly, only oltipraz showed a positive effect on adipocytes differentiation under adipogenic differentiation medium (Supplementary Figure 1C, 1D).

In summary, oltipraz is the best drug in improving trilineage differentiation abilities, rapamycin and vitamin C could effectively improve osteogenic and chondrogenic differentiation potential, while metformin only exhibits limited positive effect on osteogenic differentiation.

### Transcriptomics profile of drug treated BM-hMSCs

**Figure 3:**
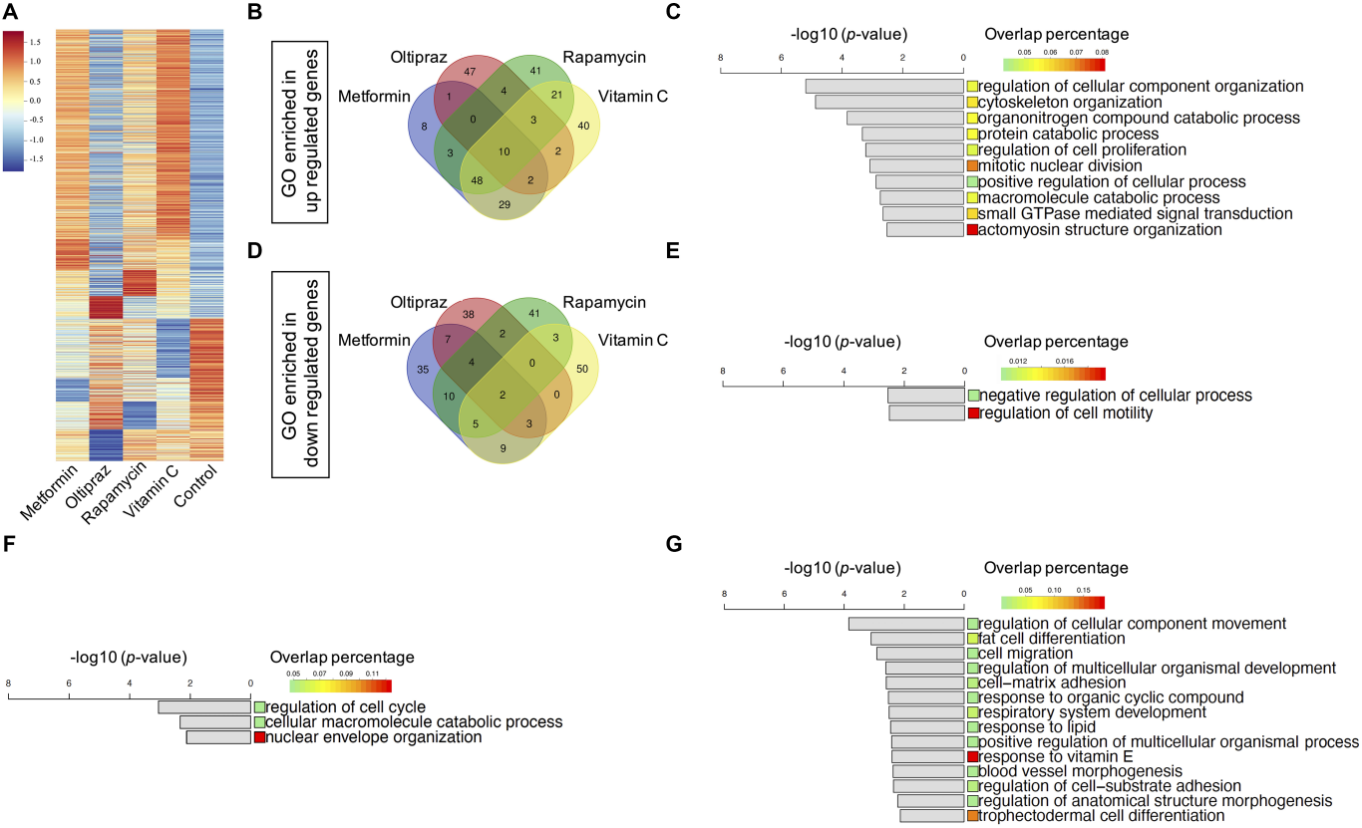
**Transcriptomics characterization of drug induced BM-hMSCs.** (**A**) 1758 DEGs with fold-change above 2 between drugs treatment group and control, which containing both upregulated and downregulated genes. (**B**) Venn diagram of the enriched upregulated GOs of the four drugs. (**C**) Ten common upregulated GOs shared by four drugs. (**D**) Venn diagram of the enriched downregulated GOs of the four drugs. (**E**) Two common downregulated GOs shared by four drugs. (**F**) Three common upregulated GOs shared by oltipraz and metformin. (**G**) Fourteen common downregulated GOs shared by oltipraz and metformin.

Given that all four drugs could counteract senescence of BM-hMSCs with diverse efficiency, next we sought to explore the underlying mechanisms for their distinct effect by investigating the transcriptomics profile of BM-hMSCs upon drug treatment. We calculated the fold-change of gene expression between each drug-control pair respectively. For each drug-control pair, the differentially expressed genes (DEGs) with fold-change above 2 were selected, which containing both upregulating and downregulating genes (Supplementary Table 1). We got 1758 genes in total after analyzing all the DEGs from the drugs treated BM-hMSCs (Figure 3A). To investigate the functions of the DEGs, we identify the enriched gene ontology (GO) terms (^**^p < 0.01) based on each drug’s DEGs [19] (Supplementary Table 2). These enriched GO terms represent the functions influenced by the drugs. Furthermore, we plot the Venn diagram to study the similarity and specificity of the enriched GO terms. As shown in Figure 3B, although each drug has its unique regulated GO terms, the drugs also share common GO which suggests that there are common functions influenced by all four drugs. These common GO terms may associate with the drugs’ effects on senescence alleviation and trilineage differentiation improvement.

Twelve common GO terms were shared by four drugs (Figure 3B, 3D), of which mainly enriched in the negative regulation of cell motility (Figure 3E), positive regulation of actomyosin structure organization, regulation of cellular process and cellular component organization, cytoskeleton organization, macromolecule catabolic process, regulation of cell proliferation, protein catabolic process and small GTPase mediated signal transduction (Figure 3C, 3E). These functions are closely related to cell fate, especially senescence related phenotypes.

After analyzing the common GO terms between metformin and oltipraz, we found that they share the same function as NRF2 agonist. As shown in Figure 3F, the function for cell cycle, macromolecule catabolic process and nuclear envelope organization were upregulated. Moreover, the function for fat/trophectodermal cell differentiation, cellular component movement, cell migration/adhesion and response to vitamin E/organic cyclic compound/lipid were downregulated (Figure 3G). These results altogether indicated that metformin and oltipraz may affect the cell differentiation abilities, cell fate and cell metabolism. As oltipraz is the best drug to improve the trilineage differentiation abilities, we further sought to identify the specific pathways altered by oltipraz. Interestingly, the enriched 85 GO terms were mainly related to cellular senescence, cell cycle, mitotic and meiosis process, cellular response to drugs, vitamin, cAMP, DNA damage stimulus, nutrient and osmotic stress (Supplementary Figure 2A, 2B).

### Lipidomics profile of drug treated BM-hMSCs

**Figure 4:**
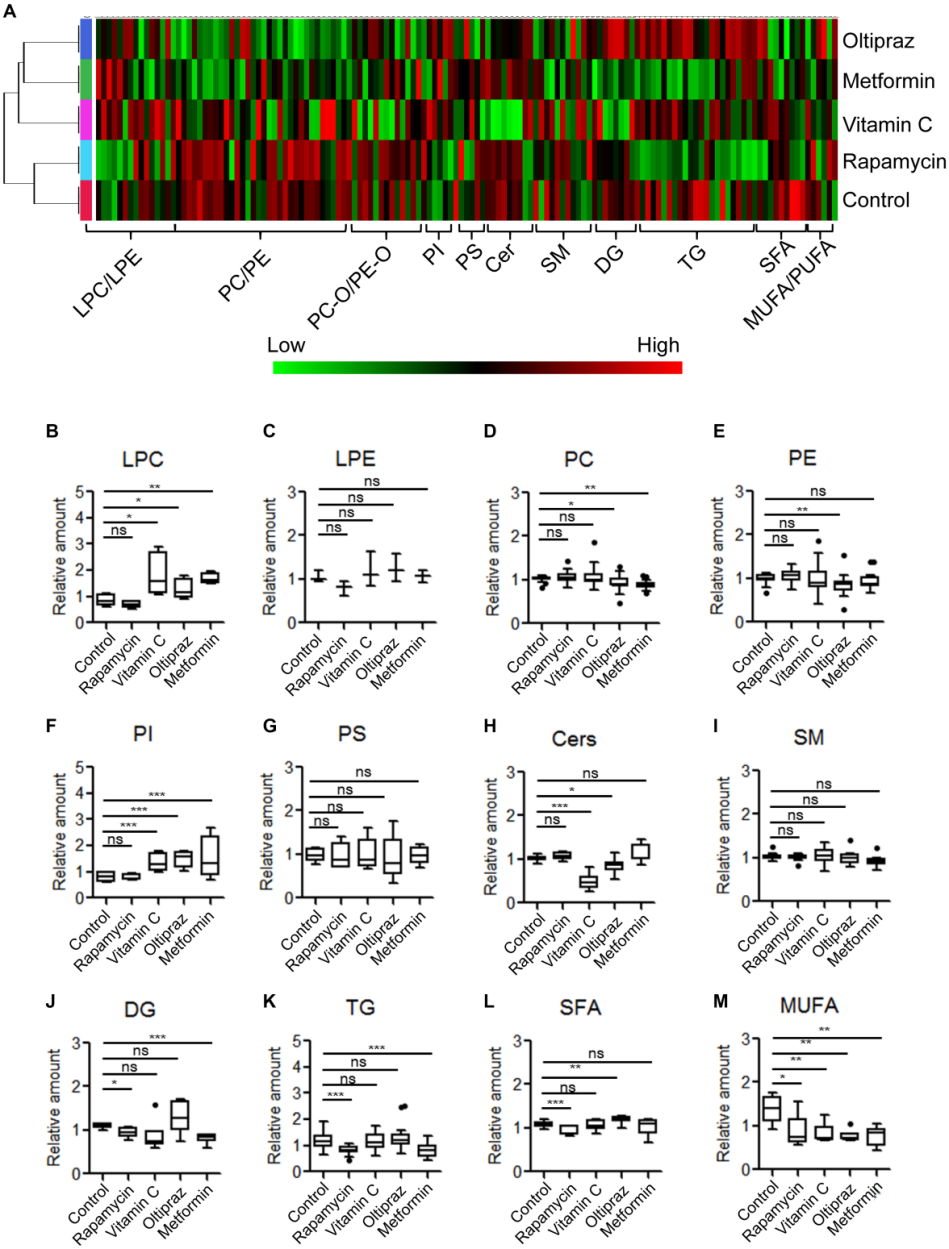
**Metabolic alterations of lipid species among MSCs induced by different drugs.** (**A**) Clustering-heatmap diagram indicated global alteration layout of various lipids among inducement groups. (**B**–**M**) Box plots showed differential significance of lipids between control and drug inducement groups.

To investigate the global fluctuation of lipids regarding to BM-hMSCs osteogenic differentiation upon drugs treatment, the LC-MS based lipidomics profiling analysis was conducted. A total of 139 lipid species were identified in cellular lipidome, including 4 lysophosphatidylcholines (LPC), 21 phosphatidylcholines (PC), 10 PC-Os, 3 lysophosphatidylethanolamines (LPE), 21 PEs, 4 PE-Os, 4 phosphatidylinositols (PI), 5 phosphatidylserines (PS), 8 Ceramides (Cer), 4 CerGs, 11 sphingomyelins (SM), 6 diacylglycerides (DG), 18 triacylglycerides (TG) and 20 free fatty acids (FFA) (Figure 4A-4M). According to the clustering matter, cells treated with oltipraz showed the largest variations of lipids, which was in agreement with its best effect on cell senescence alleviation and trilineage differentiation improvement. In detail, monophosphatidylglycerols LPCs were increased in oltipraz treatment group compared with control, whereas their counterparts PCs and PEs were down-regulated (Figure 4B, 4D, 4E). The levels of PIs and SFAs are also elevated while the level of Cers and MUFAs were reduced (Figure 4F, 4L, 4H, 4M). These results globally implicated a possible mechanism for the better efficiency of oltipraz to induce trilineage differentiation. Similarly, the increases in LPCs and PIs as well as decreases in PCs and MUFAs were also observed in the control group compared with oltipraz and metformin treatment group (Figure 4B, 4D, 4F, 4M). However, only a reduction of MUFAs were observed in all four drugs treatment groups (Figure 4M), suggesting the potential roles of MUFA in hMSCs senescence alleviation.

### Lipidomics- and transcriptomics-based pathway analysis in drug treated hMSCs

**Figure 5:**
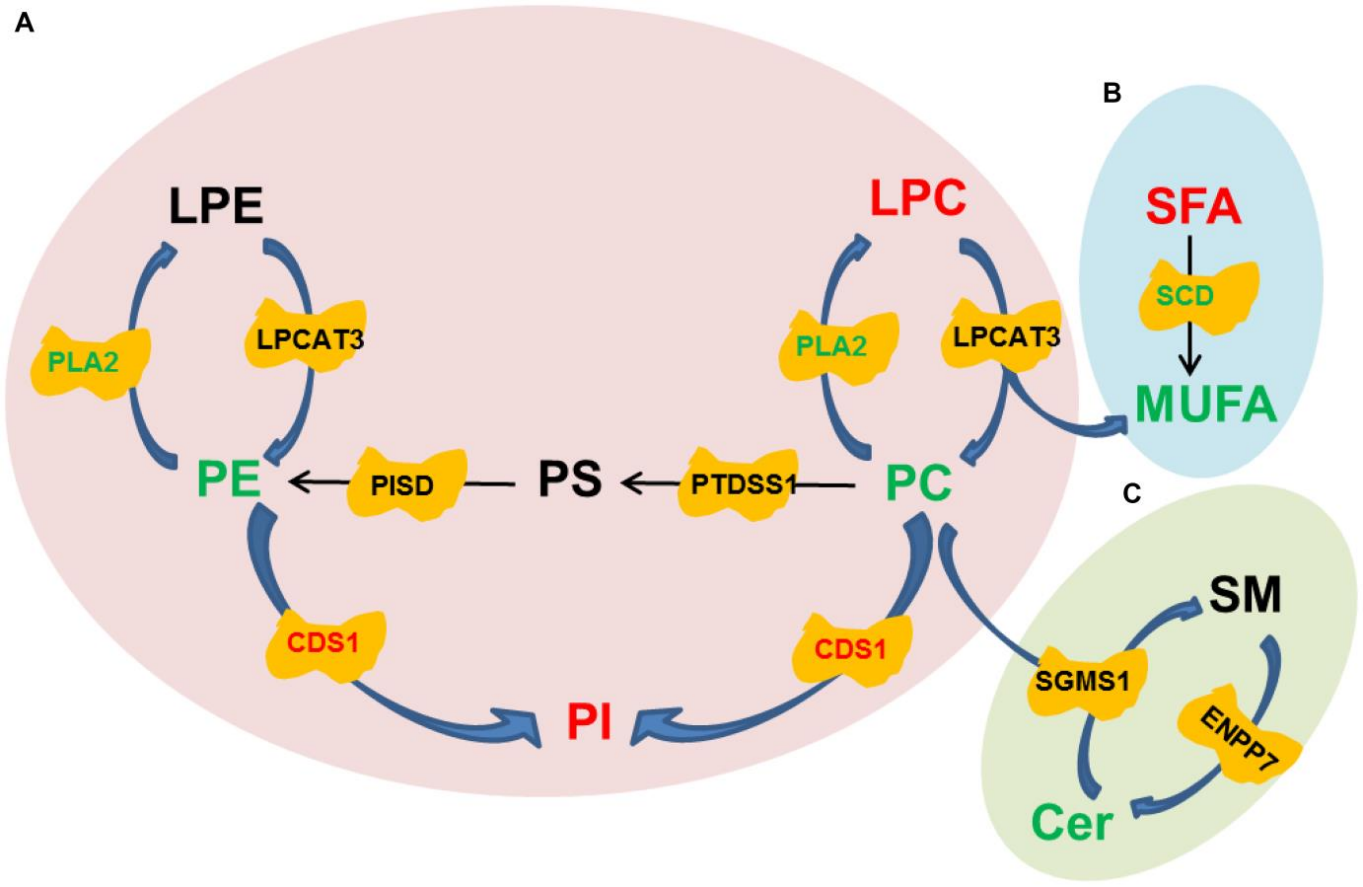
**Pathway diagrams constructed with various lipid species.** (**A**) Conversion among Glycerophospholipids. (**B**) Conversion between SFA and MUFA. (**C**) Conversion between SM and Cer.

To further investigate the underlying mechanisms for the improvement of trilineage differentiation by oltipraz, pathway analysis was performed based on the lipidomic and transcriptomic datasets. As shown in Figure 5A, CDP-Diacylglycerol Synthase 1 (CDS1) was the only enzyme up-regulated in oltipraz treated cells, which might be the reason to cause elevation of PIs and reductions of PCs. However, the down-regulated lecithin cholesterol acyl transferase (LCAT) and unchanged lysophosphatidylcholine acyltransferase3 (LPCAT3) were inconsistent with the increased LPCs, suggesting the excessively produced LPCs may not be synthesized from PCs/Pes (Figure 5A). Interestingly, an obvious conversion could be observed between SFAs and MUFAs (Figure 5B). The increase in saturated fatty acids (SFA) and decrease in MUFAs may be resulted from the down-regulated stearoyl-CoA desaturase (SCD). This layout tends to reveal the mechanism for the decreased activity of lipogenesis after treatment with oltipraz. Although oltipraz treatment could reduce the production of Cers, the conversions mediated by Cers were not significantly altered (Figure 5C).

### Global profiles of lipid species in hMSCs supernatant upon drug treatment

**Figure 6:**
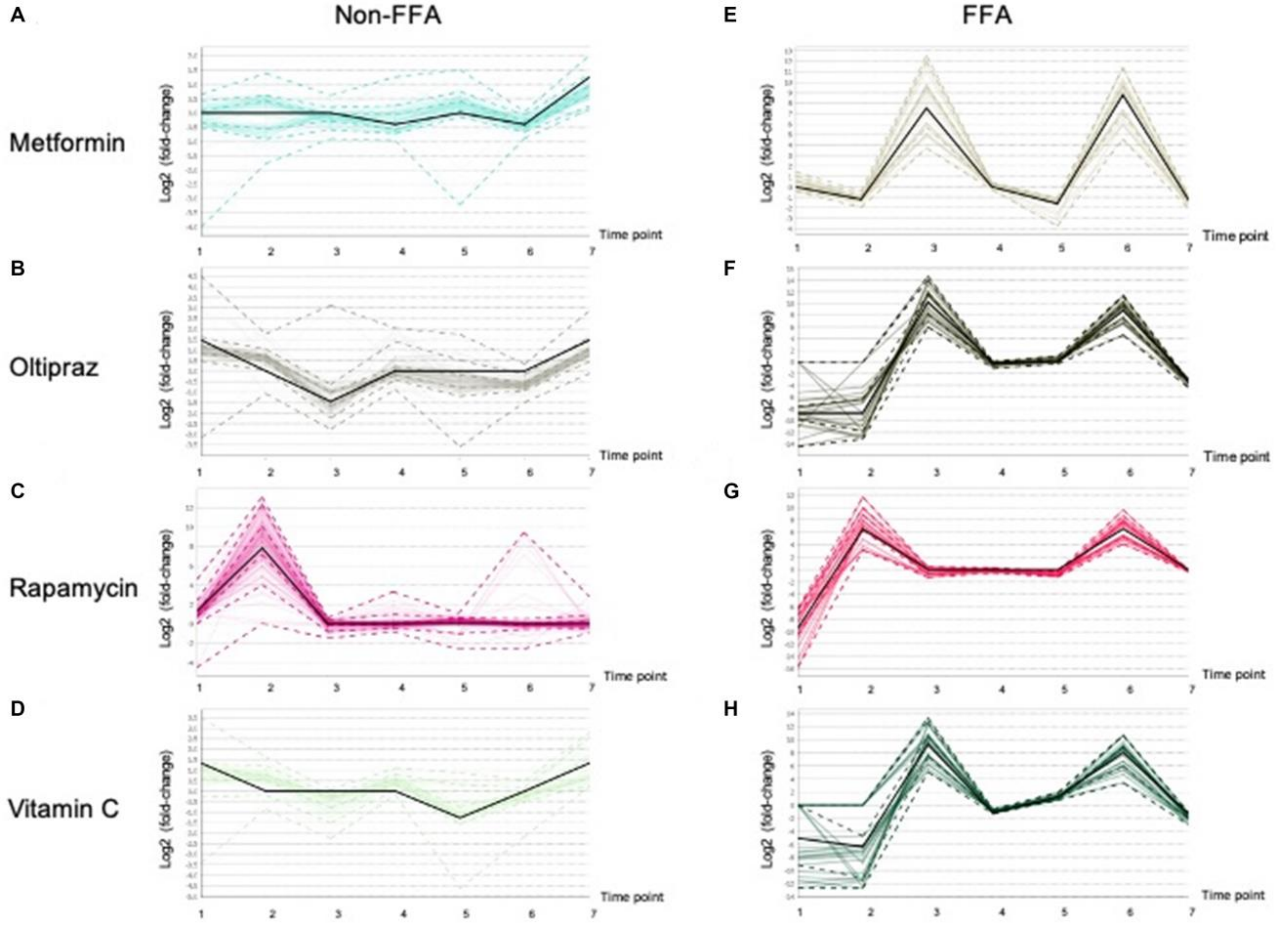
**Patterns of the Lipid fluctuation in drug treatment cells along 7 time points, and each data point is the log2 (fold-change) of a lipid between a drug and control.** The black line shows the overall trend of the cluster. (**A**), (**B**), (**C**), (**D**) showed the trends of non-FFAs in cells treated by Metformin, Oltipraz, Rapamycin and Vitamin C, respectively. (**E**), (**F**), (**G**), (**H**) presented the trends of FFAs in cells treated by Metformin, Oltipraz, Rapamycin and Vitamin C, respectively.

Furthermore, the lipidomics alterations in the medium were characterized in order to thoroughly investigate the influence of drugs on hMSCs. As shown in Supplementary Table 3, clustering analysis demonstrated that the lipids were classified into two clusters, i.e. majority of FFAs and other lipids (non-FFAs). As shown in Figure 6, in four groups, FFAs exhibited obvious alterations at different treatment periods. In contrast, non-FFAs showed slight fluctuations in four groups (Figure 6A-6D). The FFAs was significantly elevated at early stages in all groups (Figure 6E-6H). Moreover, FFAs in cells induced by metformin (Figure 6E), oltipraz and vitamin C treatment was rapidly increased from the 2nd time point to the 3rd time point. However, in the cells treated by rapamycin, FFAs was elevated from the 1st to the 2nd time point (Figure 6F-6H). Interestingly, FFAs in metformin treated cells showed a greater decline than the other three groups (Figure 6E). Then, slight rises of FFAs could be seen in oltipraz and vitamin C treatment group while from the 4th to the 5th time point, FFAs showed a slight decline in the metformin treatment group (Figure 6E, 6F, 6H).

In rapamycin treated cells, levels of FFAs were basically maintained from the 3rd to the 5th time point. Specifically, as shown in Supplementary Figure 3 and Supplementary Table 4, the alteration patterns of FFAs could be further classified into 8 clusters. Therein, cluster 6 mainly consisting of SFAs showed the same layout as those showed in oltipraz and vitamin C group, while cluster 1 was basically the same as the rapamycin group. Generally, the medium-FFAs in all groups except for metformin were globally increased along with the inducement, suggesting the association between FFAs in internal cultural environment and cell senescence. Cells induced by metformin and vitamin C contributed a similar influence to the SFAs in the internal environment compared with rapamycin induction (Figure 6A, 6D, 6E, 6H).

### The general strategy to study anti-aging effects of drugs in hMSCs

**Figure 7:**
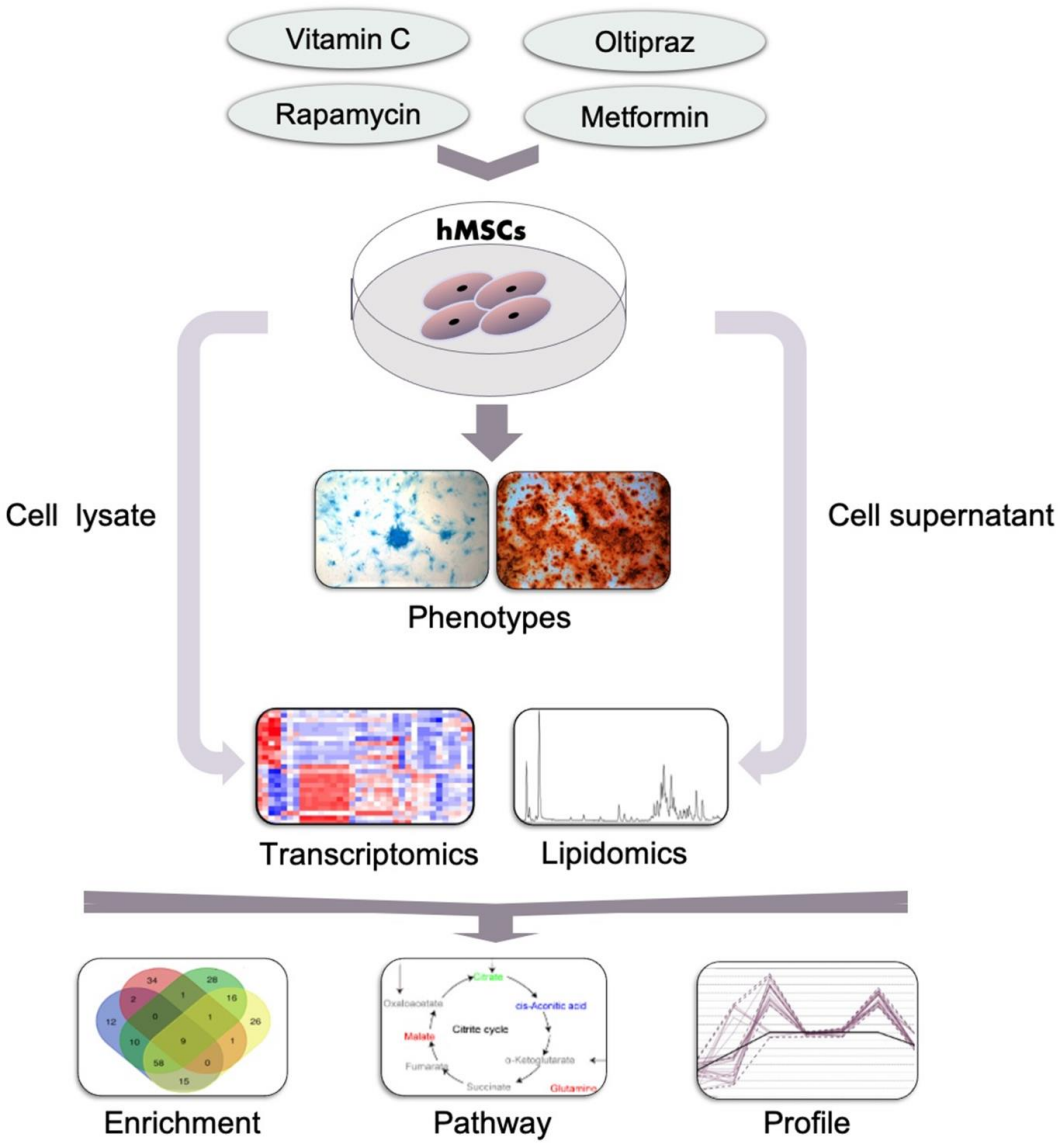
**General workflow of the strategy established in this study.** Cells such as hMSCs were treated with indicated drugs and the phenotypes were observed and determined which was used to classify the effect of drugs. Then the cells and supernatants which was collected at indicated time points were subjected to the lipidomics and transcriptomics analysis by MS. Furthermore, the altered genes, pathways and lipids were further analyzed based on the results of MS and to narrow down the underlying mechanisms for the drugs in inducing the observed phenotypes.

To sum up, as shown in Figure 7, to characterize the anti-aging effects of four drugs in hMSCs, a systematic study was conducted based on the workshop consisting of phenotype characterization, transcriptomics, cell lipidomics and medium lipidomics analysis. Oltipraz alleviated senescence and improved trilineage differentiation efficiency through promoting the production of PIs, inhibiting the biosynthesis of PCs/PEs, and blocking the conversion from SFA to MUFA. GO terms also illustrated the phenotype associated with cellular senescence, mitotic and meiosis process, cellular response to DNA damage stimulus, ammonium ion and oxygen levels. Interestingly, the NRF2 pathway was regulated by both oltipraz and metformin to promote cell differentiation at different degree. Meanwhile, the medium lipidomics layout demonstrated the critical roles of medium FFAs in hMSCs differentiation into multiple cell types. Therefore, the multidimensional strategy provides multiple perspective to interpret different effect in drug intervention on hMSCs senescence. This method can be used to study different biological phenotypes upon drug intervention in MSC which will extend the clinical application of hMSCs.

## DISCUSSION

The novel coronavirus disease 2019 (COVID-19) has grown to be a global public health emergency. Currently, there were no specific drugs to protect the patients from the infection of COVID-19, hence there is an urgent need for effective treatments for patients infected with COVID-19. hMSCs have been widely used from basic research to clinical trials in cell-based therapy. It plays roles mainly in two ways, differentiation abilities and immunomodulatory effects. It was reported that the application of MSC in COVID-19 severe cases improves the outcome of the patients by regulating the inflammatory response of the body and promoting lung tissue repair and regeneration [20]. However, frequently emerged senescence of hMSCs during longer time culture *in vitro* hampered the clinical application of hMSCs. In this study, we establish an *in vitro* drug screening system based on BM-hMSCs senescence phenotype [21]. Many studies conducted aging related intervention research for hMSCs based on manipulation of TFs [22–27] and application of different drugs [10, 13, 17, 28, 29]. However, we are the first to thoroughly assess the anti-aging effect of different drugs on BM-hMSCs from the perspective of SA-β-gal activity, osteogenic, chondrogenic, and adipogenic differentiation efficiency. Rapamycin, oltipraz, metformin and vitamin C are the four traditional drugs firstly used for hMSCs aging intervention. Although all the four drugs counteract BM-hMSCs senescence, the exact effects and underlying mechanism of the four drugs in promoting cell differentiation were different. More importantly, we also establish an overall evaluation system of drug intervention by integrated analysis of transcriptomics and lipidomics profiles in cell supernatants and lysates. In detail, transcriptomics data were fully analyzed by combination with the phenotype of stem cells after drug treatment to explore the altered pathway and underlying mechanisms influenced by drugs treatment. Moreover, the lipid change pattern of cells treated with different drugs at different time points was determined by LC-MS. Furthermore, through integrating the data of transcriptomics and lipid metabolomics, drug-specific metabolic pathways and drug-related lipid changes were analyzed and summarized.

Oltipraz is the best drug to improve the differentiation abilities of BM-hMSCs among four drugs. The DEGs affected by oltipraz treatment were mainly enriched in cellular senescence, cell cycle, and cellular response to drugs, cAMP and so on. These changes play critical roles in cellular aging. However, oltipraz and metformin, the Nrf2 pathway inhibitors, influenced common regulatory pathways [30], which mainly enriched in cell differentiation abilities, cell fate and cell metabolism. As an inhibitor of mTOR pathway, rapamycin could prevent cellular senescence [31, 32]. Consistently, we also found that rapamycin plays the best role in promoting the osteogenic differentiation which mainly through regulating phosphate-containing compound metabolic process pathway, cellular component organization, cell communication, signal transduction, macromolecule biosynthetic process, cellular protein metabolic process and skeletal system development. Vitamin C is a well-known reductant and it plays the best role in promoting cell proliferation compared with the other three drugs, but there was no significant effect in the trilineage differentiation abilities improvement of hMSC. These phenotypes may be caused by the response to cytokine/lipid/oxidative stress, positive regulation of catalytic activity (Supplementary Figure 4).

Lipids have been demonstrated to play irreplaceable roles in differentiation of stem cells [33]. We previously found that the increases of membrane glycerophospholipids including PCs and PEs, a hallmark of senescence, were globally observed in the aged MSCs [18]. In current study, the reductions in PCs and PEs were observed in the cells treated with oltipraz which performed best in delaying senescence of MSCs. PCs and PEs are the lipids with the highest abundances in the construction of eukaryotic membranes [34]. The increased levels of PCs and PEs in sera have been shown in elder people compared with younger individuals [34]. Interestingly, PIs were the minority GPLs decreased in aged MSCs in our previous observations. In this study, PIs was dramatically elevated in all induced cells. Mechanically, pathway analysis suggested that the increases in PIs may be associated with the activated CDS1. CDS1 kinase has been proved to participate into the regulation of ROS signaling and further influenced the expression of genes that functioned in oxidative resistance [35]. Another significantly blocked metabolic flux is the conversion from SFA to MUFA mediated by SCD. As a biobarometer of lipogenesis, the activated SFA/MUFA has been observed in a batch of diseases such as cancer and nonalcoholic fatty liver disease (NAFLD) [36, 37]. The similar results are also observed in aged mice, which reflects the abnormal metabolism of lipids in liver during aging [38].

Altogether, our strategy can be used in anti-aging drug screening and molecular mechanism exploration after drug treatment in stem cell and cancer. By combining the strategy with the evaluation system, we can perform the real-time monitoring based on the aging related status of stem cells to discover new drugs which will be used into stem cell therapies.

## MATERIALS AND METHODS

### Reagents and cell culture

Rapamycin (SM-V900930-1MG), oltipraz (SM-O9389-5MG) and vitamin C (SM-V900134-100G) were purchased from Sigma. Metformin (MKL-M813341-5g) was purchased from Macklin. Metformin (100 μM) [10], oltipraz (20 μM) [13], rapamycin (10 nM) [16] and vitamin C (280 μM) [17] were used to treat MSC as indicted. BM-hMSCs were purchased from Cyagen Biosciences (Guangzhou, China). The cells were cultured in human bone marrow mesenchymal stem cell medium (Cyagen Biosciences, Cat#: HUXMA-90011) according to the manufacturer’s instructions.

### hMSCs differentiation

The quality of hMSC was verified by differentiation toward bone, cartilage and adipocytes using Human Mesenchymal Stem Cell Osteogenic Differentiation Medium (Cyagen Biosciences, Cat#: UXMA-90021), Human Mesenchymal Stem Cell Chondrogenic Differentiation Medium (Cyagen Biosciences, Cat#: HUXMA-90041), and Human Mesenchymal Stem Cell adipogenic Differentiation Medium (Cyagen Biosciences, Cat#: HUXMA-90031) according to the manufacturer’s instructions. The trilineage differentiation abilities of hMSC lines were examined by histochemical staining with Alizarin red solution (osteogenesis), Alcian blue (chondrogenesis) and Oil Red O (adipogenesis), respectively.

### SA- β -gal staining assay

SA- β -gal staining was performed using Senescence β-Galactosidase Staining Kit (Cell Signaling Technology, #9860) according to the manufacturer’s protocol. Briefly, cells were fixed in 1× Fixative Solution 10–15 min at room temperature. Then stained with fresh β-Galactosidase Staining Solution at 37°C overnight. The number of cells positive for the SA- β -gal signal was observed and the percentage was analyzed.

### RNA-Seq processing

Total RNA extraction was performed by using TRIzol™ Reagent (Invitrogen, Cat#: 15596026). Library preparation and sequencing were finished by BGI company (Shenzhen, China).

Adapter and low-quality bases below a quality score of 15 were trimmed from raw RNA-Seq reads. After trimming, reads with less than 20 bp were further removed. The remaining reads were aligned to human reference genome hg38 via STAR software (version 2.5.3a) [39].

### Differentially expressed genes (DEGs) definition

The edgeR package was employed to calculate the DEGs based on the raw counts of mapped reads. Non-expressed and low-expressed genes with reads count lower than 10 in all of the five (four drugs and one control) RNA-Seq samples were filtered out using the filterByExpr function. There were 8891 genes remained for the downstream DEG analysis after the filtering step. To get the DEGs between drug-induced and control group, we calculated the fold-change of the CPM (count per million) of genes. For each drug, we got the gene list upregulated with fold-change more than 2, as well as downregulated with fold-change less than 0.5 compared with control.

### Time-series lipidomics analysis for the supernatant of hMSC

The supernatant for lipidomics analysis are collected in a time-series manner during the drug treatment. Supernatant samples were collected at three replicates at 7 time points. To study the dynamic pattern of lipidomics related to the observed phenotypes, the following procedures were conducted: 1) Normalize the lipidomics in each individual sample through dividing the original intensities by average peak intensities, which results in the relative intensity. 2) Take the mean normalized intensities across the biological replicates (samples from different wells collected at the same time-point) as the intensities for each time point in each drug or control. 3) Calculate the logarithmic fold-change between drug-control pair. 4) Utilize the TiCoNE (version 1.3) [40] to cluster the time-series fold-change data in each drug-control pair.

### Extraction of lipid species

Cells were washed with phosphate buffer saline (PBS) three times prior to harvesting. Then 1 mL of liquid nitrogen was added into culture plates for quenching. The cells were further added by 200 mL precooled-water followed by sonication at 30 Hz for 1 min. Then, the mixture was added by 200 mL ethyl acetate and vortexed for 1 min. The mixture was then centrifuged at 15000 rpm for 15 min and 180 mL of the supernatant was isolated. The extraction steps were further repeated thrice followed by combining the supernatant that was further dried via rotating-vacuum. The extraction of lipids from medium was similar to that for cells. 300 mL ethyl acetate was added into 300 mL medium followed by 1 min vortex. The mixture was further centrifuged at 15000 rpm for 15 min and 270 mL supernatant was isolated. The procedure was repeated for three times and the supernatants were combined and dried by rotating-vacuum.

### LC-MS analysis

The residual extracted from cells and medium was re-dissolved into 60 μL isopropanol-methanol solution (1:1, v/v). Chromatographic separation was conducted by an ultrahigh performance liquid chromatography system (Shimadzu, Kyoto, Japan) with a column of HSS T3 2.1 mm × 100 mm, 1.8 μm (Waters, Milford, MA, USA) which will be work at the temperature of 40°C. The mobile phase B was isopropanol/methanol (9/1, v/v) with 10 mM ammonium formate and mobile phase A consisted of 60% acetonitrile with 10 mM ammonium formate. The chromatographic gradient was run at flow rate of 0.3 mL/min and compiled as follow: maintain 10% B from 0 to 1.0 min; 10 to 80% B from 1.0 to 15.0 min; 80 to 95% B from 15.0 to 20.0 min, hold the gradient for 4 min; change B back to 10% from 24.0 to 25.0 min.

MS acquisition was conducted on a Triple TOF 5600 plus series quadrupole time-of-flight mass spectrometer (AB Sciex, Redwood City, CA, USA) in both positive and negative ion modes. The acquisition parameters were applied as follows: spray voltages: 5.5 kV and 5.0 kV for positive and negative ion modes; GS1 and GS2: 55 psi; TEM: 550°C. Mass range: m/z 300 to 1500. The identification of lipid species was conducted based on their specific fragments [41]. The differential significance among lipids of different groups was characterized by the two-sided Student’s *t*-test.

### Image analysis

ImageJ software was used for image analysis. For all the image statistical analysis, at least four fields were used for evaluation. Values are presented as the mean ± SEM. Statistical analyses were performed via the two-sided Student's *t*-test. *p* < 0.05 was considered as statistically significant, ^*^p < 0.05, ^**^p < 0.01, and ^***^p < 0.001.

### Data availability

Data generated for this study are available through the NCBI Gene Expression Omnibus (GEO) under accession number GSE165201. Lipidomic datasets are available on xcmsonline platform (https://xcmsonline.scripps.edu/landing_page.php?pgcontent=mainPage.) under accession number 1415233 and 1438843 (cell lysate in positive and negative ion modes), 1415497 and 1415529 (cell medium in positive and negative ion modes), respectively. The authors declare that all the data supporting the findings of this study are available within the article and its supplementary information files or from the corresponding author upon reasonable request.

## Supplementary Material

Supplementary Figures

Supplementary Table 1

Supplementary Table 2

Supplementary Tables 3 and 4

Supplementary References
