# Supplementary material for: Transcriptome and lipidome profile of human mesenchymal stem cells with reduced senescence and increased trilineage differentiation ability upon drug treatment
Source: Aging (Albany NY). 2021 Mar 26;13(7):9991–10014. doi: 10.18632/aging.202759 (PMC8064146; doi:10.18632/aging.202759)
Supplement: Supplementary Figures [file aging-13-202759-s001.pdf]

SUPPLEMENTARY FIGURES

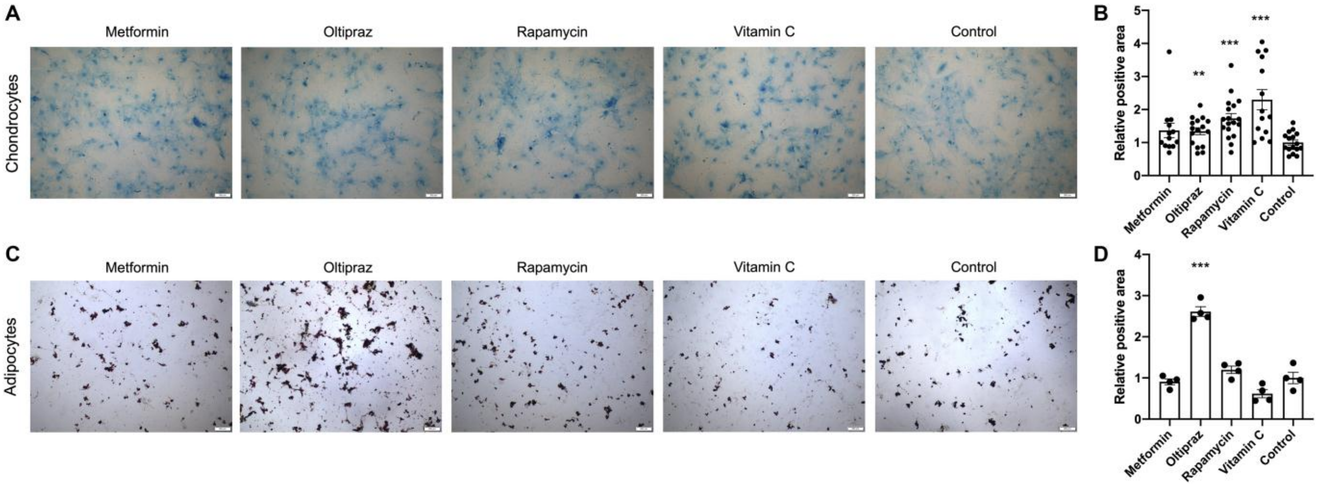

Supplementary Figure 1. The chondrogenic and adipogenic differentiation abilities of drug inducted cells. (**A**) Alcian blue staining on day 21 after transduction to label chondrocytes. Scale bar, 200 μm. (**C**) Alizarin red staining on day 21 after transduction to label osteoblast. Scale bar, 200 μm. (**B**) and (**D**) ImageJ was used for image analysis. Pictures were quantified by measuring the positive staining intensity area. Data are presented as sthe mean ± SEM, *n* > 3, $^{*}P < 0.05$, $^{**}P < 0.01$, $^{***}P < 0.001$.

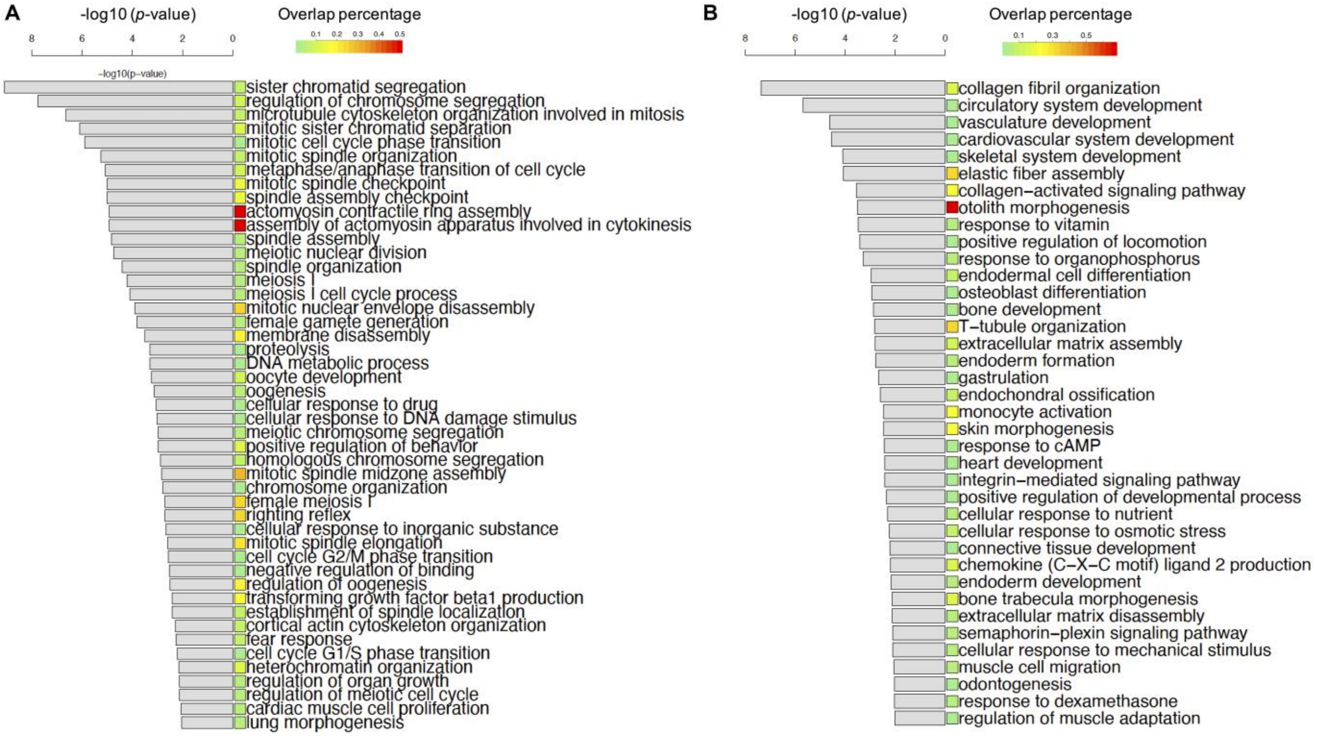

Supplementary Figure 2. Transcriptomics characterization of oltipraz induced BM-hMSCs. (**A**) Common upregulated GOs enriched in the DEG of oltipraz treated cells. (**B**) Common downregulated GOs enriched in the DEG of oltipraz treated cells.

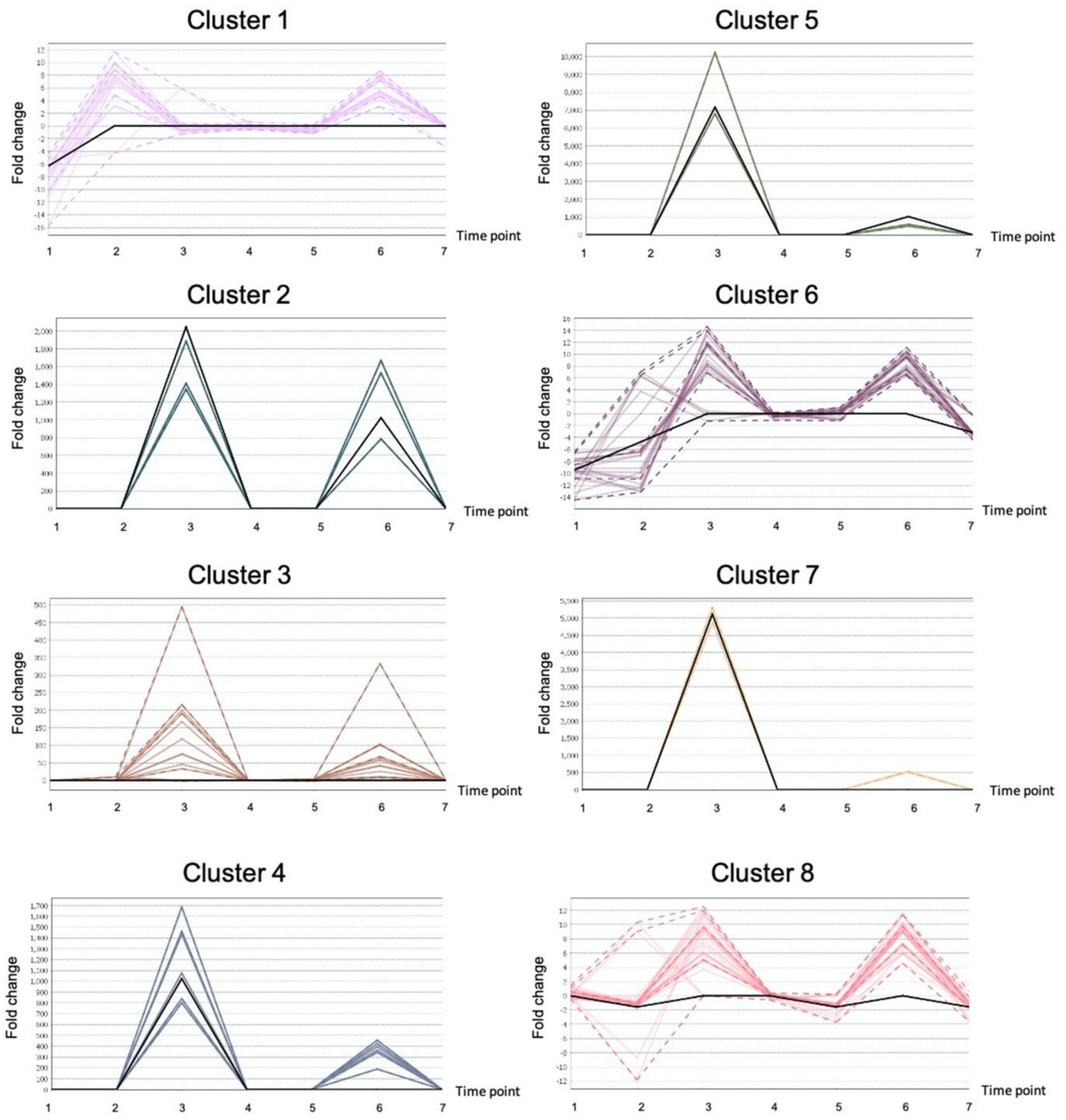

Supplementary Figure 3. Clustering of the FFA time series data. We combined all the FFAs' time-series fold-changes from the four drug-control pairs and clustered them using TiCoNE (version 1.3) [3]. Totally, we got 8 clusters. Each line shows a FFA's fluctuation, and each data point is the fold-change of a FFA between a drug and control. The black line shows the trend.

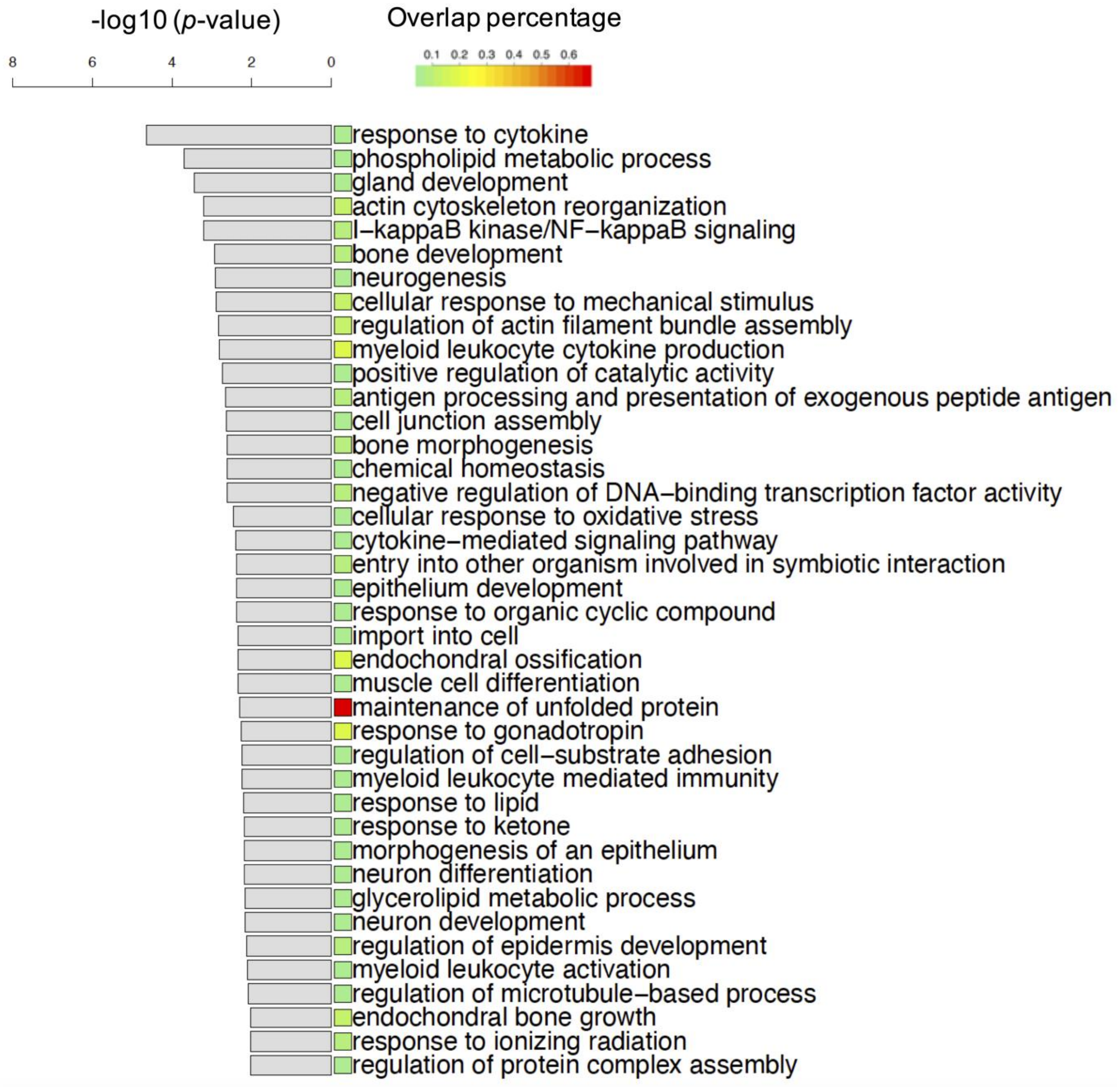

Supplementary Figure 4. Common upregulated GOs enriched in the DEG of vitamin C treated cells.
