# Supplementary material for: Transcriptome and lipidome profile of human mesenchymal stem cells with reduced senescence and increased trilineage differentiation ability upon drug treatment
Source: Aging (Albany NY). 2021 Mar 26;13(7):9991–10014. doi: 10.18632/aging.202759 (PMC8064146; doi:10.18632/aging.202759)
Supplement: Supplementary Table 1 [file aging-13-202759-s002.docx]

Supplementary Table 1. DEGs list for each drug. We supply the values of gene expression and fold-change for the all 6955 genes. The DEGs lists contain gene names with fold-change above 2 considering both up and down regulation.

| **GeneID** | **"Control (CountPerMillion, CPM)"** | **Metformin (CPM)** | **Oltipraz (CPM)** | **Rapamycin (CPM)** | **VitaminC (CPM)** |
| --- | --- | --- | --- | --- | --- |
| LASP1 | 298.88 | 582.07 | 264.75 | 475.12 | 611.27 |
| DVL2 | 3.90 | 12.27 | 4.18 | 17.39 | 15.40 |
| CCDC109B | 8.50 | 12.74 | 10.30 | 8.26 | 18.53 |
| SPPL2B | 11.62 | 33.52 | 8.33 | 27.49 | 43.88 |
| CREBBP | 12.21 | 23.05 | 8.79 | 31.08 | 25.84 |
| SPATA20 | 28.91 | 77.46 | 30.24 | 70.72 | 71.99 |
| ALDH3B1 | 11.91 | 28.92 | 15.13 | 23.75 | 28.07 |
| SCIN | 4.79 | 6.08 | 4.86 | 6.92 | 14.43 |
| MARK4 | 4.87 | 8.46 | 3.50 | 8.79 | 11.30 |
| RPUSD1 | 6.13 | 22.02 | 3.88 | 18.44 | 22.78 |
| PIGQ | 10.65 | 24.88 | 6.67 | 19.86 | 22.63 |
| CYB561 | 19.86 | 43.83 | 19.52 | 28.76 | 48.58 |
| NISCH | 26.01 | 54.85 | 23.90 | 50.98 | 57.15 |
| SCMH1 | 9.99 | 21.15 | 10.60 | 31.98 | 21.06 |
| LYPLA2 | 29.06 | 53.82 | 22.24 | 40.88 | 62.30 |
| MRC2 | 26.83 | 61.83 | 19.36 | 83.67 | 74.60 |
| APBA3 | 5.01 | 10.84 | 2.67 | 9.61 | 11.67 |
| PTBP1 | 28.98 | 71.83 | 33.11 | 72.97 | 87.20 |
| LARS2 | 5.76 | 10.21 | 6.52 | 13.80 | 14.13 |
| ANLN | 7.98 | 26.07 | 40.74 | 19.11 | 23.08 |
| CLDN11 | 139.11 | 322.19 | 388.72 | 112.91 | 327.43 |
| MVP | 112.69 | 289.36 | 173.79 | 209.11 | 335.40 |
| ANGEL1 | 5.76 | 16.96 | 6.97 | 10.02 | 13.60 |
| HEBP1 | 24.83 | 41.93 | 28.73 | 26.67 | 53.05 |
| MAMLD1 | 8.95 | 22.02 | 11.66 | 7.44 | 18.38 |
| ZC3H3 | 2.42 | 14.09 | 3.50 | 10.66 | 13.38 |
| CAPN1 | 27.50 | 80.31 | 24.88 | 64.52 | 90.85 |
| XYLT2 | 29.35 | 53.43 | 35.83 | 42.07 | 60.58 |
| PHLDB1 | 50.58 | 94.35 | 49.81 | 124.58 | 127.76 |
| CD74 | 3.31 | 4.73 | 5.84 | 2.43 | 32.92 |
| NCDN | 12.58 | 37.01 | 13.85 | 29.73 | 43.21 |
| RNF10 | 92.51 | 204.50 | 91.97 | 149.34 | 191.73 |
| RNH1 | 185.78 | 380.40 | 179.83 | 324.16 | 424.20 |
| EHD2 | 188.68 | 547.89 | 146.13 | 564.58 | 538.58 |
| RNASET2 | 4.27 | 24.47 | 4.33 | 9.46 | 15.40 |
| B4GALT7 | 21.27 | 49.54 | 20.72 | 33.62 | 49.40 |
| PNPLA6 | 22.97 | 37.01 | 14.30 | 34.37 | 48.73 |
| CHPF2 | 13.10 | 49.07 | 11.13 | 45.74 | 55.96 |
| ATP6V0A1 | 65.20 | 147.64 | 53.66 | 106.48 | 144.83 |
| MAP2K3 | 53.10 | 128.85 | 49.96 | 80.37 | 166.45 |
| EDC4 | 5.38 | 20.30 | 8.39 | 16.69 | 22.38 |
| CLEC16A | 9.91 | 27.57 | 21.48 | 24.65 | 30.98 |
| FAM65A | 45.31 | 91.18 | 35.46 | 95.93 | 116.28 |
| PQLC2 | 5.98 | 12.11 | 5.09 | 13.80 | 12.42 |
| CTNS | 6.87 | 20.20 | 11.51 | 16.04 | 22.78 |
| TNC | 42.71 | 71.83 | 52.53 | 65.79 | 129.10 |
| CAPG | 22.75 | 36.77 | 21.03 | 17.69 | 65.73 |
| CUL7 | 10.65 | 15.28 | 11.73 | 13.80 | 23.45 |
| WDR37 | 6.42 | 11.24 | 18.68 | 9.76 | 14.43 |
| TSPAN17 | 14.29 | 32.41 | 12.94 | 25.02 | 37.69 |
| ELN | 152.91 | 312.75 | 67.64 | 890.65 | 378.80 |
| NEDD4L | 3.75 | 4.58 | 4.78 | 4.00 | 20.10 |
| BCAR1 | 18.96 | 32.73 | 15.51 | 30.63 | 40.00 |
| FAM160A2 | 4.94 | 13.70 | 5.01 | 17.54 | 13.91 |
| HOMER3 | 7.54 | 12.11 | 9.68 | 11.26 | 16.14 |
| CYBA | 6.05 | 18.30 | 5.54 | 10.13 | 13.91 |
| USP13 | 5.83 | 10.60 | 9.39 | 12.23 | 13.09 |
| FLYWCH1 | 9.61 | 13.62 | 8.71 | 9.83 | 22.11 |
| PARP12 | 5.16 | 3.94 | 8.41 | 4.52 | 19.05 |
| SPHK2 | 6.28 | 8.94 | 3.27 | 14.40 | 15.17 |
| AHRR | 10.65 | 26.37 | 15.96 | 33.62 | 24.64 |
| EYA2 | 5.01 | 13.38 | 2.22 | 2.88 | 19.27 |
| CNN2 | 152.24 | 352.88 | 137.14 | 236.94 | 445.90 |
| TRAM2 | 56.29 | 138.84 | 61.52 | 136.25 | 132.08 |
| PDE4A | 5.09 | 14.96 | 8.49 | 13.50 | 11.22 |
| ELAVL1 | 18.74 | 53.19 | 24.43 | 26.74 | 52.60 |
| KDM4A | 6.65 | 11.40 | 5.16 | 12.68 | 15.47 |
| PLPP1 | 36.40 | 112.83 | 32.51 | 83.52 | 96.29 |
| STOML1 | 14.29 | 28.13 | 15.44 | 24.65 | 35.31 |
| TFE3 | 20.52 | 44.86 | 19.36 | 50.23 | 52.60 |
| ATP11A | 2.86 | 7.19 | 9.85 | 6.84 | 13.91 |
| TTC7A | 6.87 | 11.40 | 5.31 | 17.17 | 14.13 |
| DGCR2 | 9.69 | 33.36 | 7.35 | 31.68 | 40.67 |
| TRIP13 | 3.23 | 8.62 | 15.13 | 5.57 | 7.72 |
| TCF3 | 5.98 | 5.85 | 4.78 | 10.28 | 12.64 |
| CPSF1 | 9.24 | 15.28 | 8.71 | 23.52 | 23.60 |
| ZFYVE26 | 5.46 | 9.73 | 3.88 | 7.89 | 11.30 |
| ACADVL | 67.27 | 179.68 | 51.32 | 154.36 | 156.17 |
| STK10 | 13.70 | 27.34 | 12.79 | 25.77 | 34.26 |
| EVC | 4.20 | 8.86 | 2.22 | 12.75 | 9.58 |
| NDE1 | 4.72 | 10.44 | 3.88 | 12.38 | 13.68 |
| AP1M1 | 50.28 | 110.05 | 54.65 | 98.48 | 112.40 |
| SCARB1 | 5.61 | 12.19 | 4.03 | 16.12 | 17.11 |
| NUAK1 | 5.98 | 14.65 | 4.56 | 10.21 | 12.71 |
| FSCN1 | 12.66 | 54.14 | 9.62 | 45.22 | 60.66 |
| BAZ2A | 10.65 | 29.08 | 9.01 | 29.51 | 30.31 |
| SLC46A1 | 10.13 | 18.93 | 7.50 | 17.99 | 21.36 |
| PLXNA2 | 8.50 | 8.94 | 7.28 | 6.47 | 19.13 |
| SPAG5 | 3.83 | 8.78 | 20.95 | 7.07 | 9.28 |
| TRAF4 | 8.35 | 12.27 | 6.22 | 14.25 | 18.45 |
| GPC4 | 13.77 | 29.79 | 4.41 | 13.57 | 41.20 |
| ARHGEF1 | 6.94 | 17.98 | 7.28 | 18.44 | 15.77 |
| GTF3C1 | 18.67 | 54.14 | 20.50 | 43.35 | 61.70 |
| SIRT6 | 7.09 | 18.06 | 7.28 | 12.08 | 20.10 |
| FBLN1 | 19.93 | 43.12 | 21.03 | 44.32 | 58.27 |
| SDF4 | 9.47 | 114.57 | 4.18 | 34.22 | 77.21 |
| TOLLIP | 38.70 | 81.82 | 35.61 | 63.17 | 77.51 |
| SLC1A3 | 2.49 | 18.93 | 9.54 | 3.70 | 20.10 |
| CIC | 3.68 | 8.07 | 2.67 | 10.73 | 11.60 |
| MOXD1 | 18.52 | 27.02 | 15.59 | 15.97 | 50.37 |
| SRCAP | 8.09 | 28.80 | 7.74 | 25.25 | 26.00 |
| MOK | 66.31 | 51.13 | 85.17 | 29.36 | 171.15 |
| UBA5 | 22.38 | 43.12 | 22.01 | 60.33 | 51.93 |
| JMJD4 | 8.20 | 24.56 | 20.35 | 23.75 | 23.23 |
| KCNK2 | 83.60 | 126.31 | 116.07 | 60.10 | 243.32 |
| AGBL5 | 8.06 | 27.49 | 4.48 | 29.81 | 23.90 |
| MAPRE3 | 14.73 | 29.48 | 12.26 | 21.88 | 30.24 |
| CHERP | 5.24 | 16.79 | 7.88 | 17.61 | 16.07 |
| EPDR1 | 33.66 | 140.66 | 46.41 | 29.88 | 95.32 |
| TRIP6 | 14.81 | 51.44 | 9.47 | 23.30 | 49.40 |
| MMP2 | 197.73 | 417.43 | 161.85 | 299.70 | 545.74 |
| AURKA | 8.28 | 14.89 | 25.79 | 8.56 | 25.54 |
| ASAP3 | 11.17 | 22.74 | 9.09 | 16.72 | 23.15 |
| SLC23A2 | 18.45 | 43.59 | 10.90 | 42.75 | 41.72 |
| SLC8B1 | 7.46 | 16.39 | 5.31 | 13.72 | 15.25 |
| PXN | 35.96 | 102.28 | 36.74 | 90.70 | 115.68 |
| CDIP1 | 18.00 | 44.23 | 15.21 | 30.41 | 45.15 |
| PCBP4 | 10.73 | 33.60 | 9.69 | 37.29 | 37.02 |
| ICAM1 | 3.60 | 10.92 | 2.90 | 3.78 | 23.90 |
| STRN4 | 7.61 | 10.37 | 10.37 | 16.72 | 16.52 |
| P3H2 | 116.18 | 299.90 | 129.44 | 191.08 | 290.45 |
| USP48 | 17.33 | 42.17 | 14.38 | 29.96 | 37.24 |
| PLEKHG2 | 12.43 | 24.08 | 9.47 | 28.98 | 38.88 |
| DTX2 | 3.97 | 10.50 | 3.88 | 6.59 | 14.74 |
| CD200 | 1.97 | 13.70 | 13.09 | 2.50 | 13.91 |
| SLC7A8 | 5.24 | 29.48 | 17.10 | 5.65 | 40.45 |
| NUP188 | 20.89 | 60.33 | 31.07 | 58.61 | 72.88 |
| CRAT | 19.26 | 56.12 | 16.34 | 45.89 | 63.79 |
| TBC1D2 | 32.99 | 79.04 | 53.97 | 87.48 | 112.85 |
| SIRT1 | 6.13 | 9.65 | 9.77 | 6.47 | 12.94 |
| KCNK6 | 57.48 | 98.55 | 48.60 | 81.35 | 126.04 |
| HSD3B7 | 6.94 | 24.64 | 9.17 | 28.24 | 30.46 |
| POLRMT | 3.53 | 11.55 | 3.12 | 9.08 | 16.81 |
| KLHL22 | 4.57 | 17.50 | 5.46 | 16.87 | 14.13 |
| MED15 | 31.88 | 73.73 | 32.96 | 62.80 | 77.58 |
| LZTR1 | 9.02 | 25.59 | 7.35 | 24.57 | 37.99 |
| MMP11 | 5.16 | 11.55 | 1.91 | 34.30 | 14.80 |
| SF3A1 | 17.63 | 33.92 | 23.82 | 31.60 | 40.30 |
| GGT5 | 3.38 | 12.27 | 9.54 | 15.07 | 14.73 |
| SPECC1L | 17.63 | 38.44 | 23.37 | 33.77 | 37.99 |
| PPIL2 | 14.14 | 34.00 | 10.00 | 33.47 | 30.38 |
| PPM1F | 11.91 | 19.25 | 9.17 | 17.69 | 27.18 |
| TOP3B | 6.53 | 14.25 | 4.18 | 11.48 | 13.98 |
| SLC25A1 | 16.89 | 123.45 | 15.74 | 42.07 | 78.55 |
| GGA1 | 9.32 | 17.66 | 7.20 | 20.53 | 25.84 |
| GTPBP1 | 10.43 | 22.02 | 8.03 | 18.29 | 25.91 |
| PPP6R2 | 18.52 | 38.44 | 14.15 | 36.39 | 42.16 |
| LMF2 | 20.15 | 94.19 | 19.36 | 62.42 | 88.47 |
| AP1B1 | 17.18 | 61.20 | 16.19 | 47.88 | 59.16 |
| TOM1 | 62.38 | 121.23 | 37.27 | 120.32 | 128.43 |
| HMOX1 | 9.54 | 19.09 | 10.60 | 26.22 | 25.54 |
| MCAT | 5.09 | 12.98 | 3.42 | 8.34 | 17.41 |
| MCM5 | 7.54 | 18.06 | 8.64 | 10.81 | 17.93 |
| TTLL12 | 5.09 | 13.93 | 5.84 | 7.14 | 21.88 |
| TAB1 | 5.31 | 8.62 | 3.42 | 12.90 | 10.78 |
| SGSM3 | 21.41 | 56.84 | 14.68 | 45.89 | 52.16 |
| RANGAP1 | 28.69 | 55.96 | 29.03 | 33.77 | 65.35 |
| RIN3 | 5.46 | 10.05 | 5.77 | 9.83 | 14.20 |
| ITPK1 | 5.61 | 8.70 | 4.25 | 8.94 | 12.56 |
| MTHFD1 | 32.62 | 51.76 | 54.72 | 49.11 | 80.11 |
| TRPC4AP | 52.73 | 136.85 | 57.44 | 116.95 | 136.18 |
| PYGB | 47.83 | 121.39 | 48.07 | 129.52 | 132.68 |
| ABHD12 | 38.41 | 158.58 | 40.29 | 75.59 | 158.40 |
| SLC17A9 | 16.00 | 38.36 | 15.51 | 28.61 | 39.11 |
| ARFGAP1 | 14.07 | 36.38 | 11.36 | 34.59 | 37.62 |
| SNPH | 1.45 | 8.62 | 5.01 | 7.59 | 12.71 |
| ABCD1 | 4.12 | 13.70 | 4.10 | 10.43 | 14.35 |
| CCDC22 | 7.24 | 14.57 | 7.35 | 14.62 | 15.55 |
| UBL4A | 11.99 | 24.72 | 14.08 | 22.85 | 25.31 |
| CDK16 | 22.53 | 106.80 | 19.21 | 69.08 | 97.19 |
| PORCN | 10.65 | 20.75 | 7.50 | 19.33 | 30.68 |
| PLA2G15 | 49.46 | 104.82 | 36.14 | 68.18 | 135.74 |
| NAGPA | 9.24 | 20.52 | 9.17 | 25.84 | 18.53 |
| COTL1 | 170.94 | 421.95 | 225.54 | 252.80 | 422.34 |
| TSC2 | 13.55 | 30.19 | 17.32 | 28.46 | 28.97 |
| NME4 | 73.80 | 155.85 | 75.42 | 106.93 | 148.11 |
| CLCN7 | 1.90 | 13.62 | 4.71 | 12.75 | 19.35 |
| EEF2K | 6.65 | 12.35 | 12.04 | 14.92 | 14.43 |
| PIEZO1 | 55.70 | 94.90 | 35.76 | 86.81 | 113.82 |
| XYLT1 | 7.98 | 27.18 | 21.86 | 11.48 | 39.33 |
| RNF40 | 15.77 | 45.50 | 12.19 | 46.26 | 51.19 |
| CD276 | 47.24 | 85.55 | 33.11 | 109.25 | 104.94 |
| CEMIP | 144.67 | 294.75 | 48.68 | 182.18 | 519.79 |
| RPAP1 | 8.35 | 20.44 | 5.69 | 25.62 | 19.35 |
| EHD4 | 20.75 | 38.99 | 26.47 | 23.67 | 44.03 |
| VPS18 | 8.35 | 31.94 | 9.85 | 18.29 | 37.09 |
| PLAT | 155.88 | 231.31 | 299.12 | 137.23 | 407.50 |
| GSDMD | 7.46 | 11.79 | 6.45 | 13.35 | 16.81 |
| PYCRL | 9.09 | 18.85 | 4.33 | 21.43 | 25.31 |
| NEFM | 9.09 | 20.36 | 37.72 | 8.86 | 19.80 |
| TUSC3 | 36.85 | 113.30 | 31.75 | 76.93 | 83.17 |
| SARS2 | 6.13 | 10.84 | 11.05 | 6.99 | 13.53 |
| CLPTM1 | 29.50 | 182.06 | 32.59 | 93.24 | 152.44 |
| ERCC2 | 26.98 | 45.50 | 29.87 | 32.28 | 56.85 |
| DOT1L | 4.94 | 6.40 | 3.65 | 6.62 | 15.62 |
| DMPK | 16.37 | 105.21 | 10.53 | 68.93 | 101.96 |
| TBC1D17 | 12.73 | 37.49 | 11.96 | 31.45 | 33.14 |
| SNAPC2 | 9.84 | 25.04 | 8.33 | 22.48 | 28.67 |
| PPP6R1 | 9.24 | 13.35 | 4.83 | 14.55 | 19.72 |
| TYK2 | 8.28 | 18.93 | 6.67 | 19.33 | 23.90 |
| KDELR1 | 57.26 | 98.39 | 60.77 | 92.87 | 117.10 |
| GRWD1 | 20.45 | 40.18 | 26.54 | 29.43 | 45.52 |
| PLPPR2 | 22.82 | 61.44 | 9.85 | 45.07 | 57.08 |
| MIER2 | 2.49 | 18.30 | 2.74 | 12.68 | 19.13 |
| ISYNA1 | 7.39 | 12.51 | 6.97 | 14.32 | 16.67 |
| ARMC6 | 11.99 | 22.34 | 10.00 | 16.34 | 27.48 |
| FKBP8 | 13.84 | 36.30 | 8.18 | 34.15 | 33.29 |
| ATP13A1 | 12.21 | 47.40 | 19.82 | 27.11 | 46.94 |
| ZNF574 | 6.20 | 26.86 | 7.05 | 16.94 | 19.80 |
| STEAP1B | 5.24 | 6.36 | 9.40 | 2.88 | 13.64 |
| OGDH | 23.86 | 80.79 | 25.64 | 60.25 | 108.37 |
| LMBR1 | 19.93 | 50.41 | 18.61 | 27.34 | 42.76 |
| BRAT1 | 3.60 | 27.57 | 4.78 | 22.18 | 33.81 |
| TAF6 | 15.18 | 31.38 | 21.25 | 36.46 | 34.86 |
| PCOLCE | 29.35 | 59.14 | 31.30 | 71.47 | 67.81 |
| IMPDH1 | 23.49 | 82.14 | 19.21 | 68.85 | 88.61 |
| SFRP4 | 45.90 | 85.70 | 53.66 | 35.64 | 111.43 |
| AEBP1 | 12.88 | 10.68 | 11.36 | 39.98 | 34.26 |
| YKT6 | 45.46 | 92.84 | 35.38 | 75.14 | 102.56 |
| CLIP2 | 4.49 | 16.87 | 11.05 | 9.61 | 18.23 |
| ENG | 202.26 | 555.74 | 186.25 | 419.46 | 567.88 |
| TBC1D13 | 15.55 | 40.82 | 9.24 | 25.77 | 34.78 |
| DDX58 | 26.01 | 25.51 | 43.99 | 17.54 | 52.83 |
| NPDC1 | 25.35 | 54.30 | 15.74 | 35.42 | 52.01 |
| ZFAND5 | 109.40 | 225.56 | 83.07 | 209.52 | 236.85 |
| DNMBP | 9.17 | 19.33 | 19.52 | 17.61 | 24.42 |
| MAPK8 | 10.50 | 17.82 | 13.02 | 21.05 | 21.44 |
| C10orf54 | 127.68 | 286.50 | 67.64 | 170.06 | 346.59 |
| GIT1 | 6.57 | 12.03 | 6.90 | 11.03 | 17.26 |
| PSMD3 | 18.37 | 71.59 | 16.57 | 48.28 | 72.06 |
| PNPO | 22.38 | 42.17 | 28.43 | 26.96 | 46.49 |
| CAMTA2 | 20.45 | 38.12 | 16.49 | 33.17 | 42.76 |
| CYTH1 | 4.64 | 10.05 | 5.61 | 12.68 | 10.33 |
| RAB5C | 37.67 | 90.30 | 57.14 | 72.15 | 90.85 |
| VAT1 | 166.19 | 413.31 | 164.49 | 300.67 | 478.93 |
| HDAC5 | 16.81 | 31.54 | 7.28 | 30.93 | 37.09 |
| DPH1 | 25.41 | 60.13 | 14.54 | 53.85 | 64.87 |
| SLC9A3R1 | 3.60 | 10.05 | 5.24 | 6.99 | 13.24 |
| TMEM104 | 18.30 | 36.38 | 20.35 | 24.20 | 48.50 |
| UNC119 | 9.91 | 43.36 | 11.73 | 22.70 | 31.80 |
| SLAIN2 | 16.96 | 66.99 | 22.01 | 40.50 | 47.68 |
| USP46 | 4.64 | 18.30 | 15.21 | 13.28 | 10.85 |
| TRIM2 | 4.79 | 12.03 | 9.54 | 9.76 | 13.16 |
| NCAPG | 2.19 | 9.57 | 14.00 | 8.56 | 6.15 |
| VWA5A | 6.79 | 15.12 | 10.37 | 13.13 | 13.91 |
| DNAJC4 | 7.76 | 14.49 | 11.58 | 13.80 | 17.26 |
| EHD1 | 70.39 | 205.21 | 71.19 | 145.23 | 228.26 |
| ST3GAL4 | 6.05 | 15.84 | 5.09 | 15.15 | 17.41 |
| CCND1 | 1064.71 | 2221.20 | 1720.79 | 1095.69 | 2268.87 |
| CCDC86 | 12.43 | 19.72 | 14.61 | 21.65 | 26.36 |
| TRIM3 | 9.02 | 16.63 | 7.50 | 14.62 | 23.97 |
| ARHGEF17 | 35.59 | 76.19 | 52.15 | 64.89 | 89.14 |
| MDK | 14.29 | 18.53 | 18.53 | 13.57 | 33.81 |
| TCIRG1 | 15.92 | 68.68 | 14.68 | 63.37 | 71.54 |
| MVK | 5.01 | 14.96 | 7.28 | 11.03 | 13.68 |
| KRT18 | 136.07 | 74.13 | 86.00 | 43.12 | 351.51 |
| TNS2 | 4.94 | 7.27 | 4.18 | 12.75 | 12.71 |
| ACAD10 | 5.01 | 11.87 | 5.39 | 16.34 | 11.97 |
| LTBR | 47.46 | 107.75 | 38.33 | 83.74 | 137.15 |
| VDR | 21.12 | 35.98 | 23.44 | 33.17 | 45.74 |
| USP5 | 45.83 | 104.58 | 50.41 | 81.12 | 107.18 |
| PPARD | 6.28 | 20.67 | 4.93 | 17.17 | 21.36 |
| PEX7 | 4.79 | 15.92 | 3.27 | 5.79 | 13.01 |
| CCND3 | 26.83 | 105.69 | 41.65 | 56.89 | 131.71 |
| NUDT12 | 6.42 | 12.82 | 14.91 | 12.23 | 12.86 |
| DPYSL3 | 7.54 | 15.20 | 10.83 | 3.92 | 22.78 |
| HMGXB3 | 19.86 | 54.85 | 27.22 | 40.58 | 45.52 |
| DBN1 | 53.84 | 123.45 | 43.84 | 106.86 | 162.13 |
| AMOTL2 | 112.47 | 227.42 | 122.19 | 201.71 | 262.19 |
| KLHL18 | 13.32 | 20.52 | 12.11 | 24.65 | 28.67 |
| SCAP | 11.10 | 26.54 | 10.30 | 31.45 | 29.94 |
| MAPKAPK3 | 16.07 | 26.23 | 12.79 | 36.32 | 36.20 |
| ABHD14B | 16.52 | 39.87 | 14.08 | 41.85 | 41.34 |
| EIF4G1 | 100.37 | 191.10 | 114.56 | 166.85 | 242.06 |
| STEAP3 | 10.50 | 21.63 | 11.58 | 31.53 | 31.88 |
| GTF3C2 | 13.10 | 31.30 | 12.11 | 27.26 | 29.86 |
| ID2 | 5.46 | 29.16 | 14.30 | 11.78 | 26.88 |
| HPCAL1 | 29.13 | 102.67 | 26.01 | 75.14 | 111.73 |
| SDC1 | 4.35 | 19.49 | 6.45 | 7.67 | 22.33 |
| PHF13 | 6.65 | 18.53 | 8.33 | 18.29 | 15.62 |
| AMPD2 | 31.51 | 72.30 | 23.67 | 60.33 | 73.40 |
| MEF2D | 18.15 | 39.23 | 12.94 | 44.17 | 42.54 |
| LEPR | 54.01 | 124.51 | 76.41 | 51.32 | 119.73 |
| MIIP | 6.50 | 14.17 | 8.64 | 11.93 | 14.43 |
| OLFML3 | 12.58 | 42.96 | 16.80 | 26.37 | 62.07 |
| ECE1 | 35.22 | 87.92 | 38.33 | 100.65 | 81.68 |
| IPO13 | 19.86 | 46.21 | 16.87 | 17.84 | 41.42 |
| STMN1 | 61.63 | 104.42 | 110.70 | 67.58 | 146.85 |
| RCN2 | 62.08 | 164.93 | 53.21 | 126.15 | 152.66 |
| CTSD | 146.98 | 369.69 | 83.27 | 426.28 | 359.02 |
| NRP2 | 17.04 | 33.13 | 8.49 | 15.37 | 54.47 |
| TNFAIP3 | 11.47 | 21.86 | 33.57 | 22.18 | 32.92 |
| PIGZ | 2.71 | 10.05 | 3.12 | 9.24 | 12.12 |
| WDR34 | 19.63 | 38.12 | 31.60 | 35.19 | 42.39 |
| PTPA | 17.85 | 41.29 | 15.36 | 42.75 | 46.34 |
| ZBTB45 | 6.28 | 15.28 | 6.22 | 13.42 | 15.55 |
| GPR68 | 4.94 | 14.17 | 5.01 | 15.82 | 12.34 |
| TMEM214 | 26.16 | 59.77 | 29.03 | 44.77 | 59.61 |
| OGFRL1 | 8.50 | 11.55 | 11.36 | 11.78 | 17.63 |
| IFIT3 | 7.83 | 11.00 | 28.51 | 11.48 | 40.45 |
| HSPH1 | 47.68 | 139.00 | 83.05 | 84.56 | 123.43 |
| SORBS3 | 26.76 | 70.79 | 17.32 | 63.84 | 73.40 |
| PPP3CC | 15.92 | 47.88 | 11.05 | 23.82 | 37.24 |
| ADCY7 | 13.10 | 26.07 | 15.89 | 14.02 | 31.06 |
| ANXA11 | 45.23 | 133.37 | 38.40 | 112.32 | 140.28 |
| NAA60 | 16.37 | 29.56 | 7.20 | 29.73 | 38.96 |
| PLAU | 92.73 | 125.20 | 79.12 | 71.62 | 211.41 |
| ACADS | 2.49 | 11.71 | 1.99 | 8.79 | 12.12 |
| DDX54 | 10.50 | 24.16 | 6.29 | 20.31 | 26.21 |
| ADGRE5 | 75.59 | 138.24 | 68.47 | 110.93 | 195.94 |
| NR4A1 | 7.24 | 22.90 | 11.96 | 15.07 | 14.73 |
| TUBA1B | 918.07 | 1847.65 | 1245.79 | 1358.66 | 1978.40 |
| SLC12A4 | 54.58 | 155.97 | 36.59 | 134.46 | 154.08 |
| EREG | 10.06 | 13.85 | 19.29 | 14.17 | 23.97 |
| MT2A | 85.46 | 166.67 | 223.95 | 107.30 | 172.64 |
| HS3ST3B1 | 8.35 | 12.11 | 11.36 | 4.75 | 19.80 |
| GGA3 | 2.56 | 12.03 | 4.56 | 14.17 | 12.49 |
| MBOAT7 | 50.35 | 95.93 | 50.04 | 71.47 | 112.10 |
| PPDPF | 9.91 | 20.91 | 11.36 | 18.51 | 22.70 |
| VASP | 7.83 | 17.98 | 9.47 | 15.07 | 18.01 |
| SYMPK | 2.64 | 12.19 | 5.39 | 7.52 | 12.56 |
| NRSN2 | 29.13 | 56.20 | 17.02 | 44.69 | 59.24 |
| NCLN | 19.93 | 56.28 | 19.29 | 45.74 | 73.70 |
| GDF5 | 7.61 | 11.48 | 7.73 | 16.94 | 28.82 |
| ID1 | 6.87 | 34.32 | 17.85 | 15.89 | 24.42 |
| RALY | 50.06 | 106.64 | 35.00 | 82.32 | 116.95 |
| TMEM115 | 38.11 | 89.51 | 31.07 | 78.35 | 89.51 |
| HECTD3 | 14.14 | 38.60 | 12.57 | 28.01 | 30.53 |
| KLC1 | 76.55 | 193.66 | 72.09 | 98.59 | 167.08 |
| FRMD8 | 13.84 | 34.55 | 11.13 | 26.22 | 35.60 |
| SCAF1 | 5.24 | 9.41 | 5.31 | 5.27 | 13.01 |
| GLIS2 | 7.17 | 9.57 | 5.16 | 14.47 | 14.88 |
| ELK1 | 10.73 | 26.07 | 13.32 | 19.56 | 23.15 |
| IDUA | 2.64 | 20.04 | 1.54 | 9.31 | 15.70 |
| FGFRL1 | 19.93 | 45.18 | 21.33 | 25.02 | 49.84 |
| SIN3B | 10.58 | 22.02 | 10.15 | 16.27 | 31.95 |
| WDR24 | 4.94 | 12.03 | 3.88 | 9.24 | 12.56 |
| TICAM1 | 4.72 | 9.65 | 3.27 | 11.26 | 12.79 |
| TNFRSF19 | 2.27 | 13.93 | 4.18 | 7.07 | 14.50 |
| POR | 24.38 | 65.40 | 14.76 | 55.39 | 66.69 |
| ZFP36 | 12.58 | 38.36 | 11.58 | 37.21 | 55.66 |
| CDC42EP1 | 26.24 | 78.80 | 15.81 | 105.58 | 88.09 |
| TST | 8.65 | 17.11 | 8.56 | 8.04 | 22.70 |
| RAC2 | 21.19 | 40.98 | 33.64 | 20.98 | 50.74 |
| CPA4 | 14.44 | 41.93 | 23.90 | 10.58 | 48.50 |
| PODXL | 20.67 | 42.17 | 33.27 | 22.78 | 50.96 |
| FLNC | 117.81 | 256.88 | 87.69 | 236.59 | 357.14 |
| ARHGAP22 | 11.40 | 22.18 | 20.27 | 14.62 | 35.45 |
| ISLR | 43.01 | 159.38 | 66.28 | 40.50 | 87.65 |
| PUS7L | 9.39 | 19.41 | 20.50 | 20.01 | 22.78 |
| SLC44A2 | 96.51 | 232.10 | 108.21 | 202.60 | 225.21 |
| ADCY4 | 13.79 | 42.89 | 15.84 | 19.78 | 44.23 |
| DOHH | 2.05 | 13.62 | 1.16 | 7.96 | 15.85 |
| MAU2 | 11.54 | 25.91 | 16.64 | 29.13 | 29.56 |
| NECTIN2 | 21.04 | 59.53 | 22.24 | 37.14 | 64.83 |
| TOMM40 | 5.31 | 13.46 | 3.35 | 10.73 | 13.83 |
| SLC27A1 | 6.50 | 14.96 | 5.69 | 15.07 | 21.51 |
| ACTN4 | 33.14 | 78.25 | 45.81 | 59.65 | 84.22 |
| MAP1S | 6.13 | 27.65 | 6.67 | 17.69 | 35.68 |
| ASS1 | 5.98 | 16.71 | 5.92 | 7.22 | 22.18 |
| SESN2 | 19.26 | 35.50 | 14.30 | 46.41 | 43.73 |
| EMC8 | 14.81 | 40.42 | 9.47 | 34.59 | 39.48 |
| SH3BP5 | 8.95 | 16.55 | 11.13 | 7.96 | 18.08 |
| PDLIM4 | 44.12 | 75.47 | 48.22 | 59.21 | 89.51 |
| MGAT1 | 115.21 | 265.64 | 95.67 | 196.10 | 267.26 |
| GFPT2 | 11.25 | 18.69 | 18.31 | 9.83 | 33.44 |
| TRAF7 | 18.52 | 34.47 | 12.34 | 30.70 | 39.78 |
| TOP2A | 7.91 | 27.73 | 37.04 | 13.50 | 20.92 |
| STARD3 | 16.96 | 28.76 | 23.60 | 23.75 | 38.14 |
| CC2D1A | 5.76 | 8.22 | 6.97 | 12.00 | 11.74 |
| LRRC41 | 104.68 | 218.85 | 79.05 | 174.48 | 212.76 |
| DHX30 | 18.74 | 46.29 | 21.71 | 48.43 | 54.39 |
| SCLY | 3.69 | 9.80 | 6.14 | 9.95 | 14.13 |
| MYBBP1A | 10.73 | 25.67 | 13.32 | 22.78 | 29.27 |
| SERPINF1 | 8.28 | 11.79 | 17.63 | 6.54 | 25.02 |
| MATN2 | 5.83 | 9.73 | 6.97 | 6.17 | 16.14 |
| VPS4A | 24.45 | 66.24 | 32.69 | 54.85 | 80.84 |
| PCED1A | 8.35 | 15.44 | 7.05 | 11.78 | 19.05 |
| DPH2 | 11.77 | 24.16 | 16.64 | 30.26 | 29.27 |
| BEX1 | 14.81 | 10.13 | 12.34 | 2.80 | 33.52 |
| HSPBP1 | 20.89 | 37.88 | 23.22 | 36.17 | 48.80 |
| ARF3 | 43.24 | 81.74 | 49.30 | 68.54 | 99.93 |
| SLC43A3 | 8.72 | 12.19 | 14.23 | 11.63 | 19.87 |
| DHX34 | 2.12 | 11.24 | 2.14 | 9.98 | 10.18 |
| RAB11FIP5 | 24.90 | 39.39 | 16.95 | 43.57 | 59.09 |
| FHOD1 | 8.35 | 11.24 | 11.36 | 11.85 | 17.56 |
| SERPINE2 | 2036.46 | 4844.09 | 1969.27 | 1561.65 | 4356.66 |
| FLNB | 89.54 | 128.37 | 93.93 | 158.40 | 186.44 |
| TBC1D4 | 10.13 | 23.53 | 14.98 | 10.88 | 23.08 |
| TNS3 | 10.36 | 27.10 | 14.61 | 18.14 | 23.38 |
| RAC1 | 66.55 | 185.94 | 61.55 | 99.35 | 145.55 |
| TTYH3 | 9.84 | 16.00 | 4.63 | 19.63 | 21.51 |
| RSAD1 | 4.79 | 18.30 | 6.07 | 23.37 | 20.47 |
| TACO1 | 6.65 | 22.18 | 8.33 | 15.37 | 20.84 |
| SMPD4 | 15.78 | 33.78 | 9.24 | 31.28 | 38.23 |
| SAP130 | 3.90 | 8.78 | 5.39 | 11.85 | 8.99 |
| FAM129B | 14.66 | 33.05 | 17.32 | 40.50 | 42.02 |
| ST6GALNAC4 | 7.91 | 21.71 | 11.13 | 20.76 | 29.56 |
| SLC2A8 | 5.31 | 14.33 | 4.63 | 10.66 | 12.56 |
| USP20 | 5.61 | 17.82 | 3.58 | 6.69 | 18.83 |
| IL11RA | 8.43 | 22.87 | 7.05 | 17.65 | 27.77 |
| TMEM63B | 5.01 | 12.11 | 5.61 | 12.08 | 15.47 |
| TUBB2A | 86.31 | 161.07 | 69.93 | 138.98 | 183.17 |
| HMGA1 | 99.26 | 332.58 | 307.13 | 190.94 | 392.44 |
| MTCH1 | 89.02 | 284.36 | 82.75 | 116.06 | 238.33 |
| NUMA1 | 21.34 | 37.09 | 18.68 | 53.74 | 46.94 |
| SULF1 | 74.40 | 157.32 | 107.08 | 106.18 | 348.53 |
| FDX1 | 8.35 | 25.35 | 6.14 | 11.11 | 20.62 |
| MAPKBP1 | 4.87 | 8.22 | 2.22 | 12.60 | 11.82 |
| CYP1B1 | 230.16 | 711.57 | 254.92 | 198.19 | 583.91 |
| PREB | 49.98 | 90.30 | 34.40 | 92.34 | 111.21 |
| LOXL4 | 21.93 | 38.91 | 14.98 | 34.07 | 59.09 |
| MYPN | 1.53 | 5.92 | 16.12 | 1.91 | 13.98 |
| HECW2 | 12.73 | 23.13 | 32.81 | 26.29 | 33.66 |
| PARP9 | 7.98 | 8.94 | 17.48 | 17.09 | 17.41 |
| RGS3 | 68.46 | 102.83 | 74.29 | 61.52 | 152.74 |
| GUCD1 | 22.01 | 39.39 | 23.22 | 27.26 | 49.17 |
| KIAA1644 | 7.83 | 17.50 | 11.43 | 5.12 | 18.68 |
| PHLDA1 | 29.50 | 104.66 | 114.40 | 70.35 | 126.86 |
| DUSP6 | 11.25 | 38.60 | 32.74 | 11.85 | 52.68 |
| RITA1 | 9.32 | 16.71 | 10.30 | 16.12 | 19.42 |
| ANKRD52 | 22.75 | 73.33 | 7.43 | 58.76 | 73.40 |
| VPS37B | 7.16 | 16.06 | 4.02 | 19.54 | 16.25 |
| FGF7 | 76.43 | 196.96 | 93.13 | 50.60 | 202.32 |
| PML | 29.72 | 59.06 | 28.58 | 61.15 | 77.73 |
| POLG | 13.47 | 32.81 | 17.10 | 29.28 | 31.58 |
| FURIN | 11.62 | 27.57 | 9.62 | 21.73 | 31.28 |
| SLC5A2 | 7.69 | 20.28 | 6.90 | 16.94 | 23.45 |
| C16orf58 | 18.15 | 44.94 | 12.64 | 40.06 | 46.79 |
| RHOT2 | 18.15 | 37.57 | 10.68 | 30.41 | 46.49 |
| SLC14A1 | 14.81 | 56.68 | 32.43 | 13.72 | 95.32 |
| MINK1 | 6.79 | 23.85 | 8.03 | 23.75 | 30.09 |
| ARHGDIA | 88.79 | 207.44 | 78.29 | 186.90 | 188.67 |
| ERBB2 | 16.59 | 38.36 | 15.96 | 35.79 | 41.05 |
| FKBP10 | 9.47 | 23.77 | 14.08 | 35.19 | 30.24 |
| SLC39A3 | 14.22 | 30.98 | 17.17 | 23.22 | 30.98 |
| NFIC | 11.84 | 19.17 | 12.26 | 24.87 | 27.33 |
| SH3GL1 | 16.44 | 46.37 | 16.80 | 40.13 | 55.21 |
| DPP9 | 15.03 | 32.89 | 17.85 | 26.44 | 40.38 |
| CCDC97 | 7.02 | 13.78 | 6.97 | 15.82 | 16.52 |
| SCYL1 | 55.10 | 109.42 | 45.50 | 94.66 | 126.49 |
| DOPEY2 | 7.46 | 15.28 | 11.13 | 13.95 | 19.27 |
| URB1 | 10.50 | 22.50 | 16.57 | 12.90 | 27.48 |
| RNPEPL1 | 3.83 | 12.35 | 3.95 | 13.95 | 18.01 |
| C19orf52 | 3.83 | 14.25 | 3.50 | 4.97 | 11.97 |
| PEX14 | 5.90 | 12.27 | 5.69 | 10.73 | 13.01 |
| MAP3K6 | 3.75 | 12.19 | 7.35 | 10.36 | 12.64 |
| HSPG2 | 71.21 | 157.39 | 59.25 | 228.26 | 144.83 |
| CREG1 | 30.54 | 96.33 | 21.56 | 73.19 | 67.89 |
| PI4KB | 57.63 | 96.17 | 46.49 | 90.62 | 127.09 |
| SELENBP1 | 2.86 | 15.60 | 4.18 | 7.44 | 16.96 |
| DYRK3 | 5.68 | 19.01 | 4.86 | 6.69 | 17.86 |
| ADAM15 | 22.08 | 59.30 | 14.08 | 52.70 | 70.05 |
| JTB | 32.84 | 91.65 | 24.88 | 68.85 | 100.54 |
| GALNT2 | 100.00 | 193.79 | 59.63 | 154.43 | 209.47 |
| MALL | 2.24 | 12.59 | 7.43 | 2.97 | 10.75 |
| SLC20A1 | 96.66 | 165.96 | 191.31 | 139.17 | 208.65 |
| POMGNT2 | 5.76 | 11.71 | 6.97 | 8.26 | 16.29 |
| CSRNP1 | 9.39 | 28.45 | 12.87 | 30.03 | 27.48 |
| LIMD1 | 5.90 | 12.98 | 8.41 | 15.52 | 12.04 |
| CCNA2 | 4.27 | 10.84 | 13.09 | 6.47 | 11.37 |
| USP53 | 17.11 | 40.58 | 31.83 | 14.17 | 40.82 |
| HAPLN1 | 25.57 | 51.29 | 24.12 | 15.15 | 54.84 |
| TNFRSF21 | 7.76 | 14.73 | 8.03 | 5.27 | 21.51 |
| TPBG | 20.82 | 51.68 | 14.61 | 23.15 | 42.54 |
| EGFR | 20.15 | 32.09 | 31.15 | 20.98 | 46.27 |
| SIGMAR1 | 73.21 | 179.20 | 86.68 | 122.49 | 190.76 |
| SLC25A25 | 4.87 | 8.30 | 5.54 | 7.74 | 12.27 |
| FAM73B | 4.87 | 9.18 | 5.31 | 7.89 | 16.07 |
| PTGES | 74.18 | 144.63 | 55.78 | 71.85 | 179.20 |
| LRSAM1 | 7.31 | 15.84 | 12.26 | 17.39 | 15.40 |
| NOTCH1 | 5.01 | 14.17 | 5.69 | 17.31 | 13.68 |
| INA | 3.83 | 16.31 | 7.88 | 4.82 | 22.85 |
| PPRC1 | 7.91 | 26.46 | 5.77 | 23.00 | 27.77 |
| ITPRIP | 23.94 | 50.57 | 23.67 | 33.62 | 55.51 |
| TUT1 | 4.93 | 14.66 | 4.21 | 8.39 | 11.47 |
| CAPN5 | 10.95 | 22.26 | 7.05 | 9.98 | 24.05 |
| PAK1 | 24.53 | 48.03 | 41.27 | 34.30 | 54.24 |
| NUDT22 | 13.55 | 31.22 | 18.39 | 22.63 | 29.79 |
| CDC42EP2 | 15.85 | 35.19 | 14.91 | 27.94 | 42.61 |
| TAOK2 | 5.98 | 15.68 | 5.01 | 12.90 | 22.78 |
| LYPD1 | 3.68 | 10.37 | 11.28 | 6.84 | 26.06 |
| ABCB9 | 10.36 | 17.58 | 15.13 | 13.95 | 22.26 |
| INPP1 | 10.13 | 14.33 | 10.37 | 15.52 | 20.54 |
| BAG3 | 22.23 | 60.49 | 20.42 | 24.42 | 58.34 |
| AP1S3 | 5.38 | 9.10 | 7.88 | 4.82 | 12.56 |
| ZFP36L2 | 21.56 | 35.11 | 16.80 | 25.32 | 43.13 |
| PID1 | 4.57 | 14.09 | 7.96 | 3.63 | 11.60 |
| AK5 | 16.22 | 29.24 | 35.91 | 19.93 | 36.13 |
| THY1 | 237.06 | 337.02 | 241.70 | 188.17 | 501.45 |
| ANKH | 18.00 | 28.37 | 21.86 | 12.83 | 43.06 |
| MAGED4 | 1.37 | 47.26 | 17.44 | 16.17 | 24.69 |
| CXXC1 | 7.83 | 15.60 | 5.77 | 16.94 | 16.14 |
| MARVELD1 | 27.80 | 43.04 | 37.19 | 35.94 | 57.15 |
| GRAMD3 | 69.51 | 119.73 | 75.72 | 78.43 | 157.28 |
| MOV10 | 19.04 | 47.32 | 11.51 | 43.72 | 53.50 |
| KCNMA1 | 4.27 | 8.70 | 5.46 | 5.72 | 13.53 |
| SH3RF2 | 4.12 | 15.84 | 8.26 | 6.99 | 18.23 |
| FBRS | 3.83 | 8.70 | 7.28 | 12.08 | 11.07 |
| TMEM171 | 13.10 | 18.06 | 33.64 | 14.10 | 29.12 |
| NRG1 | 22.01 | 22.97 | 11.58 | 16.72 | 45.67 |
| STEAP2 | 4.64 | 12.51 | 15.29 | 4.00 | 11.37 |
| MMP14 | 8.06 | 20.67 | 5.01 | 13.87 | 25.76 |
| CCNB2 | 5.53 | 9.73 | 20.27 | 8.11 | 12.49 |
| ETS2 | 5.53 | 7.03 | 6.45 | 6.99 | 11.74 |
| SLC35B2 | 48.57 | 96.41 | 47.39 | 66.39 | 117.99 |
| SLC38A10 | 14.81 | 47.00 | 14.53 | 33.85 | 41.72 |
| LDLRAP1 | 20.23 | 59.22 | 17.25 | 50.38 | 55.73 |
| EXTL1 | 12.43 | 37.01 | 9.85 | 26.44 | 41.05 |
| XKR8 | 6.50 | 11.79 | 5.99 | 15.82 | 13.09 |
| FAM160B2 | 7.46 | 21.63 | 11.05 | 20.91 | 23.30 |
| FBXW5 | 40.56 | 88.08 | 33.64 | 78.06 | 81.46 |
| ALDH4A1 | 10.95 | 33.52 | 6.37 | 33.70 | 44.62 |
| PSKH1 | 4.35 | 16.79 | 3.35 | 18.59 | 14.80 |
| ZYX | 140.74 | 483.57 | 160.41 | 339.12 | 517.78 |
| ABR | 12.73 | 32.41 | 11.51 | 28.46 | 41.72 |
| RRP1 | 8.80 | 22.50 | 16.34 | 25.84 | 23.67 |
| C21orf2 | 6.28 | 15.84 | 4.18 | 10.81 | 13.38 |
| SPATC1L | 4.94 | 17.66 | 4.18 | 15.82 | 13.16 |
| LSS | 23.12 | 54.93 | 15.44 | 51.05 | 55.73 |
| SLC2A6 | 4.49 | 17.90 | 9.09 | 14.17 | 20.69 |
| GPSM1 | 5.38 | 12.11 | 5.46 | 12.98 | 13.38 |
| SHKBP1 | 19.78 | 44.23 | 26.16 | 40.35 | 50.29 |
| PLPP7 | 12.14 | 28.13 | 8.86 | 20.23 | 26.21 |
| SHC1 | 111.95 | 237.25 | 84.79 | 208.44 | 298.42 |
| NLRX1 | 3.97 | 12.19 | 5.84 | 12.68 | 19.65 |
| LMNA | 383.40 | 920.69 | 482.32 | 801.11 | 976.01 |
| LRWD1 | 6.20 | 14.96 | 7.81 | 10.13 | 17.26 |
| PGAP3 | 5.83 | 13.70 | 4.10 | 13.05 | 12.71 |
| CYGB | 6.35 | 13.30 | 9.09 | 8.71 | 14.13 |
| NEU3 | 3.60 | 11.40 | 5.09 | 11.63 | 12.19 |
| LRP5 | 6.65 | 15.52 | 4.48 | 10.88 | 16.44 |
| TPCN2 | 6.13 | 17.34 | 7.88 | 12.90 | 16.96 |
| PLPP3 | 69.50 | 242.80 | 155.05 | 163.71 | 218.35 |
| SEPN1 | 8.87 | 12.11 | 10.37 | 9.54 | 18.68 |
| MXRA8 | 48.28 | 345.27 | 33.79 | 311.44 | 380.44 |
| VCAM1 | 5.31 | 14.89 | 3.73 | 5.72 | 14.95 |
| OLFML2B | 21.86 | 25.12 | 11.58 | 57.48 | 49.25 |
| SPATA18 | 14.36 | 21.23 | 24.88 | 22.55 | 33.66 |
| CLDN1 | 20.08 | 22.50 | 15.66 | 19.78 | 45.22 |
| PYGO2 | 3.46 | 15.68 | 3.12 | 11.78 | 13.53 |
| ATP1A1 | 56.51 | 107.51 | 99.07 | 81.35 | 115.61 |
| TMEM79 | 4.49 | 12.19 | 5.92 | 7.81 | 10.63 |
| IL17RC | 3.53 | 15.60 | 4.33 | 11.55 | 13.38 |
| ZDHHC3 | 27.57 | 60.72 | 17.10 | 50.83 | 60.80 |
| HEYL | 7.61 | 9.97 | 5.84 | 41.55 | 20.54 |
| SHISA5 | 146.83 | 304.03 | 138.73 | 253.25 | 349.65 |
| RNF123 | 6.50 | 28.13 | 11.96 | 28.91 | 28.00 |
| FAM198B | 32.03 | 66.59 | 30.55 | 30.33 | 69.53 |
| CITED2 | 18.52 | 51.37 | 17.55 | 29.13 | 50.74 |
| STEAP1 | 5.83 | 13.42 | 14.60 | 2.95 | 17.83 |
| SBSPON | 4.05 | 15.12 | 2.97 | 3.25 | 18.83 |
| INTS1 | 6.50 | 13.85 | 9.47 | 16.04 | 20.32 |
| FASTK | 25.27 | 72.54 | 27.30 | 61.67 | 71.47 |
| INTS8 | 9.24 | 25.51 | 14.83 | 22.33 | 24.05 |
| NOL6 | 13.77 | 30.51 | 7.43 | 13.72 | 35.53 |
| INPPL1 | 18.45 | 39.63 | 12.11 | 43.87 | 62.82 |
| NSMF | 5.98 | 8.78 | 5.31 | 9.68 | 12.12 |
| SLC39A13 | 67.65 | 177.46 | 54.19 | 142.84 | 175.48 |
| TRMT61A | 3.90 | 8.70 | 5.77 | 5.79 | 13.24 |
| PLEKHF1 | 7.46 | 15.36 | 6.82 | 15.82 | 17.49 |
| APBB1 | 23.71 | 62.07 | 21.25 | 48.51 | 64.53 |
| MAPK7 | 9.76 | 40.34 | 7.88 | 40.43 | 37.62 |
| ANPEP | 25.49 | 79.76 | 39.31 | 46.79 | 125.89 |
| TERF2IP | 25.20 | 78.57 | 23.44 | 28.83 | 68.41 |
| PLK1 | 4.27 | 15.52 | 8.56 | 9.31 | 11.52 |
| NAB2 | 16.66 | 29.56 | 14.61 | 28.09 | 35.90 |
| STAT6 | 24.38 | 51.37 | 29.19 | 67.28 | 66.47 |
| MAP1A | 258.06 | 634.41 | 216.77 | 510.12 | 575.86 |
| ACSF2 | 9.69 | 25.04 | 14.45 | 21.95 | 24.42 |
| SLC27A4 | 11.77 | 15.52 | 10.22 | 16.94 | 27.77 |
| IGF2 | 15.62 | 109.55 | 12.87 | 38.39 | 86.82 |
| TBC1D16 | 6.94 | 8.62 | 8.64 | 9.91 | 15.10 |
| STIM1 | 21.93 | 53.35 | 31.60 | 59.50 | 56.63 |
| ZNF668 | 8.13 | 19.25 | 4.56 | 25.69 | 18.60 |
| GATAD2A | 6.65 | 10.52 | 10.75 | 9.01 | 17.19 |
| MVD | 9.61 | 25.35 | 7.96 | 24.72 | 27.25 |
| TP53I13 | 13.47 | 35.50 | 15.13 | 24.12 | 38.66 |
| KMT2D | 5.61 | 7.99 | 4.48 | 9.01 | 15.99 |
| CORO6 | 5.90 | 19.09 | 5.84 | 17.02 | 17.41 |
| TRAPPC9 | 2.49 | 7.35 | 2.44 | 10.43 | 12.86 |
| DAPK3 | 22.30 | 42.09 | 14.23 | 39.16 | 53.94 |
| MFSD3 | 7.46 | 19.88 | 6.52 | 11.63 | 20.17 |
| SPRYD3 | 42.56 | 105.13 | 45.66 | 79.25 | 101.74 |
| IGFBP6 | 58.96 | 179.28 | 68.62 | 164.98 | 162.20 |
| TK1 | 20.23 | 50.26 | 51.40 | 25.09 | 42.69 |
| FAM234A | 76.27 | 148.17 | 34.21 | 136.75 | 157.94 |
| E4F1 | 5.76 | 16.87 | 7.58 | 14.85 | 16.74 |
| KCTD5 | 12.58 | 28.37 | 16.42 | 23.00 | 33.07 |
| VPS37C | 4.87 | 11.55 | 7.13 | 10.66 | 10.55 |
| LTBP3 | 10.95 | 24.08 | 6.82 | 20.83 | 28.30 |
| PBK | 2.12 | 6.64 | 14.30 | 4.22 | 10.40 |
| VASN | 16.07 | 66.83 | 14.83 | 28.01 | 62.74 |
| MFSD2A | 3.01 | 11.71 | 3.95 | 5.12 | 11.74 |
| ATG4B | 22.75 | 44.47 | 32.21 | 40.13 | 47.61 |
| MTCL1 | 5.68 | 13.93 | 8.33 | 13.65 | 17.71 |
| SERINC2 | 127.90 | 249.39 | 119.09 | 184.43 | 321.54 |
| SLC20A2 | 42.41 | 73.41 | 62.50 | 51.43 | 109.27 |
| ZSWIM1 | 4.12 | 10.13 | 10.15 | 11.78 | 11.89 |
| PPIP5K1 | 8.89 | 26.11 | 11.85 | 23.90 | 29.64 |
| CHTF8 | 13.40 | 36.54 | 15.29 | 40.50 | 39.55 |
| ATOH8 | 3.38 | 13.14 | 3.73 | 6.24 | 11.82 |
| SLC35G2 | 9.02 | 15.76 | 15.44 | 12.60 | 19.13 |
| TMEM129 | 3.68 | 12.51 | 4.56 | 10.51 | 16.67 |
| CPT1C | 7.46 | 15.60 | 12.41 | 16.42 | 18.01 |
| LUZP1 | 25.20 | 34.47 | 39.54 | 36.69 | 50.96 |
| SPNS1 | 10.90 | 53.45 | 8.36 | 25.64 | 39.43 |
| ASPSCR1 | 5.83 | 13.06 | 7.05 | 6.62 | 12.19 |
| FASN | 5.76 | 17.98 | 5.24 | 15.52 | 22.48 |
| NLGN1 | 3.60 | 10.76 | 13.70 | 8.11 | 10.85 |
| TPST1 | 28.39 | 48.03 | 23.97 | 36.39 | 62.00 |
| TM4SF1 | 99.18 | 257.32 | 308.26 | 123.54 | 387.45 |
| PUSL1 | 5.76 | 11.40 | 5.92 | 11.33 | 12.56 |
| NFRKB | 6.87 | 14.96 | 7.73 | 12.38 | 16.59 |
| B3GNT2 | 44.05 | 99.42 | 52.38 | 70.87 | 95.32 |
| KRT8 | 12.51 | 9.49 | 9.39 | 6.02 | 35.60 |
| RASA4B | 5.59 | 13.29 | 3.70 | 14.64 | 21.06 |
| TSEN34 | 9.32 | 40.66 | 9.32 | 16.42 | 31.73 |
| TRH | 17.93 | 19.17 | 8.56 | 12.30 | 66.84 |
| ATP6V0E2 | 8.80 | 18.06 | 6.90 | 16.64 | 22.03 |
| JUNB | 7.31 | 27.89 | 6.90 | 50.08 | 32.02 |
| SHCBP1 | 6.72 | 15.28 | 24.28 | 11.03 | 16.14 |
| ZDHHC16 | 23.94 | 71.67 | 19.44 | 38.78 | 78.47 |
| KRT19 | 16.14 | 52.24 | 16.80 | 15.89 | 215.22 |
| LRRC8D | 10.36 | 24.08 | 18.23 | 15.22 | 24.49 |
| BCL2L1 | 46.05 | 118.30 | 53.89 | 102.29 | 132.98 |
| ZNF584 | 5.46 | 11.71 | 6.22 | 12.23 | 12.34 |
| ENC1 | 23.27 | 36.30 | 24.58 | 27.41 | 55.29 |
| RRM2 | 4.87 | 16.63 | 24.65 | 15.52 | 23.15 |
| TLN2 | 5.98 | 10.21 | 8.94 | 9.09 | 13.98 |
| THOP1 | 7.91 | 17.26 | 6.37 | 11.03 | 18.23 |
| HCFC1 | 9.02 | 26.15 | 10.98 | 19.71 | 25.24 |
| SLFN11 | 7.49 | 14.96 | 10.95 | 13.29 | 17.90 |
| RARG | 4.27 | 19.80 | 5.77 | 18.14 | 34.41 |
| SSH3 | 3.83 | 15.92 | 8.79 | 12.45 | 13.16 |
| DHCR7 | 33.58 | 63.02 | 32.74 | 57.86 | 72.96 |
| MRGPRF | 10.36 | 23.53 | 8.33 | 24.57 | 34.71 |
| FAM222B | 9.84 | 24.40 | 14.45 | 25.84 | 21.29 |
| RHOD | 8.72 | 16.71 | 7.35 | 12.08 | 21.29 |
| MAP3K11 | 4.12 | 13.06 | 4.56 | 9.98 | 12.79 |
| RNF26 | 23.05 | 89.67 | 24.73 | 72.74 | 72.44 |
| CSPG4 | 30.91 | 76.82 | 23.07 | 140.07 | 81.38 |
| PC | 6.35 | 11.40 | 4.86 | 11.78 | 14.95 |
| PLK3 | 10.73 | 30.03 | 6.97 | 21.73 | 34.93 |
| HOXB2 | 1.67 | 16.23 | 2.67 | 5.50 | 10.70 |
| CTSF | 13.55 | 38.84 | 7.35 | 34.37 | 28.15 |
| RGMB | 114.17 | 234.16 | 203.63 | 170.44 | 241.46 |
| CTU2 | 6.94 | 14.01 | 5.77 | 12.83 | 15.32 |
| AKIRIN1 | 60.22 | 125.12 | 52.61 | 89.35 | 124.18 |
| RIN1 | 1.90 | 11.87 | 4.41 | 13.65 | 10.33 |
| CD248 | 186.16 | 834.09 | 114.86 | 1024.93 | 867.31 |
| RAB1B | 46.12 | 121.47 | 46.71 | 91.74 | 109.86 |
| KCTD13 | 5.53 | 21.55 | 5.69 | 14.62 | 23.45 |
| KLC2 | 5.53 | 25.75 | 2.37 | 25.17 | 23.45 |
| UBE2C | 3.97 | 9.49 | 13.70 | 7.07 | 9.73 |
| PACS1 | 13.25 | 28.21 | 13.32 | 31.08 | 33.81 |
| MED16 | 17.55 | 72.62 | 18.99 | 60.55 | 66.77 |
| TP53I11 | 81.23 | 265.41 | 52.76 | 256.99 | 240.12 |
| TOM1L2 | 22.97 | 45.58 | 17.78 | 32.87 | 55.88 |
| MSRA | 5.31 | 11.32 | 5.92 | 8.79 | 11.97 |
| BAIAP2 | 11.77 | 34.24 | 9.92 | 29.21 | 37.02 |
| SPHK1 | 10.80 | 31.78 | 17.17 | 20.61 | 44.85 |
| WDR25 | 9.54 | 16.15 | 14.23 | 14.70 | 20.32 |
| LMNB2 | 4.51 | 8.62 | 7.14 | 11.28 | 14.89 |
| DPP7 | 17.78 | 54.22 | 14.53 | 44.69 | 45.37 |
| SEC24C | 48.94 | 93.24 | 46.34 | 83.67 | 112.55 |
| PDDC1 | 5.09 | 13.54 | 6.60 | 7.22 | 15.32 |
| MIEF2 | 9.24 | 16.87 | 4.18 | 13.28 | 22.63 |
| SLC25A22 | 4.49 | 19.88 | 4.18 | 10.96 | 19.13 |
| RABEP2 | 4.57 | 9.97 | 4.78 | 10.66 | 14.88 |
| PNPLA2 | 40.19 | 87.29 | 43.16 | 46.56 | 84.29 |
| FLII | 58.52 | 154.38 | 56.91 | 119.20 | 147.14 |
| TMEM187 | 5.61 | 9.41 | 4.41 | 8.86 | 12.94 |
| ADAMTSL1 | 19.93 | 27.10 | 33.64 | 23.82 | 50.22 |
| KDELC2 | 15.33 | 34.47 | 21.48 | 31.90 | 45.15 |
| PLEC | 60.30 | 175.71 | 40.52 | 235.74 | 242.95 |
| WDR6 | 28.24 | 56.44 | 31.00 | 70.28 | 71.76 |
| FAM220A | 3.75 | 22.10 | 7.05 | 15.37 | 15.70 |
| P4HTM | 5.68 | 21.23 | 4.63 | 9.83 | 17.41 |
| GRINA | 47.98 | 104.34 | 43.69 | 75.59 | 106.73 |
| FAM101A | 3.16 | 8.54 | 3.58 | 1.61 | 18.53 |
| GAK | 6.94 | 21.94 | 6.60 | 13.28 | 21.96 |
| GADD45GIP1 | 31.28 | 74.84 | 46.49 | 48.13 | 63.49 |
| SHARPIN | 15.55 | 33.60 | 15.13 | 28.76 | 41.05 |
| CDC42EP4 | 4.72 | 16.87 | 6.22 | 10.36 | 12.79 |
| MAF1 | 25.64 | 109.50 | 20.20 | 78.65 | 96.59 |
| MYADM | 83.45 | 167.94 | 80.48 | 149.87 | 217.75 |
| MROH1 | 5.31 | 13.85 | 6.37 | 13.50 | 14.65 |
| RCC1 | 5.84 | 9.33 | 13.63 | 15.15 | 15.08 |
| GREM2 | 6.13 | 34.39 | 6.45 | 11.93 | 42.54 |
| AEN | 31.28 | 61.36 | 32.28 | 59.06 | 65.35 |
| EHMT1 | 6.05 | 21.71 | 4.86 | 21.20 | 20.99 |
| PENK | 21.41 | 27.42 | 24.12 | 25.02 | 71.32 |
| POLR2A | 42.93 | 129.80 | 51.02 | 126.60 | 125.52 |
| OGFOD3 | 5.68 | 14.89 | 2.74 | 15.07 | 16.29 |
| SGSH | 18.07 | 54.93 | 6.60 | 42.45 | 52.30 |
| SLC35C1 | 19.41 | 39.39 | 27.67 | 31.00 | 43.28 |
| TMEM259 | 6.20 | 15.60 | 6.60 | 14.40 | 25.61 |
| TSEN54 | 3.31 | 7.59 | 9.92 | 9.61 | 11.89 |
| UBA7 | 6.79 | 18.93 | 15.21 | 29.81 | 19.35 |
| MOB2 | 3.08 | 11.48 | 3.42 | 12.23 | 7.35 |
| HHIPL1 | 12.14 | 21.23 | 10.30 | 15.15 | 27.63 |
| EXOC7 | 41.45 | 93.55 | 55.55 | 92.19 | 104.50 |
| MFSD5 | 29.35 | 61.75 | 32.36 | 33.55 | 67.89 |
| NTM | 11.15 | 21.07 | 22.46 | 14.92 | 27.77 |
| TSKU | 15.33 | 40.42 | 14.00 | 41.55 | 33.14 |
| GAS6 | 159.59 | 870.65 | 115.01 | 383.03 | 934.86 |
| TMEM119 | 14.59 | 17.03 | 1.16 | 51.20 | 37.77 |
| CCBE1 | 20.52 | 58.42 | 63.03 | 31.23 | 63.19 |
| FHL3 | 18.07 | 40.02 | 11.81 | 47.31 | 38.06 |
| OR7E38P | 6.11 | 13.68 | 6.66 | 11.38 | 15.31 |
| TBL3 | 22.01 | 53.35 | 27.22 | 43.20 | 54.91 |
| GPR39 | 1.75 | 7.35 | 7.81 | 4.30 | 17.86 |
| SMTN | 33.29 | 101.09 | 37.95 | 75.21 | 110.16 |
| SCFD2 | 33.66 | 48.51 | 46.56 | 26.81 | 80.11 |
| IRAK1 | 16.07 | 23.37 | 14.61 | 25.09 | 34.19 |
| ALDH1A3 | 14.66 | 22.66 | 33.87 | 6.47 | 68.41 |
| CSF1 | 13.55 | 39.87 | 5.92 | 25.02 | 48.20 |
| TXNRD2 | 5.61 | 11.08 | 10.22 | 8.34 | 12.04 |
| MED12 | 9.76 | 14.89 | 11.58 | 13.95 | 19.87 |
| 9-Sep | 35.14 | 111.32 | 33.04 | 83.14 | 96.44 |
| 5-Sep | 10.38 | 28.61 | 9.82 | 37.25 | 36.06 |
| PRR16 | 23.64 | 61.04 | 19.36 | 41.78 | 54.02 |
| TMED9 | 42.86 | 110.13 | 60.92 | 72.30 | 106.58 |
| NOC4L | 5.38 | 11.87 | 4.41 | 11.33 | 13.09 |
| TMEM106A | 7.17 | 11.24 | 3.35 | 7.89 | 15.92 |
| MAFF | 26.61 | 52.32 | 30.39 | 34.67 | 56.55 |
| INTS5 | 3.31 | 14.57 | 5.39 | 15.59 | 14.50 |
| HSF1 | 26.98 | 69.92 | 17.32 | 71.47 | 79.52 |
| C6orf120 | 9.99 | 19.09 | 10.00 | 12.45 | 21.88 |
| RAB11B | 17.63 | 72.14 | 14.98 | 52.17 | 63.26 |
| CDK10 | 18.30 | 38.36 | 24.43 | 33.92 | 43.13 |
| HGS | 31.36 | 103.47 | 24.80 | 92.72 | 104.50 |
| FAAP100 | 4.42 | 20.99 | 5.31 | 23.52 | 21.21 |
| C11orf87 | 22.75 | 31.94 | 19.97 | 21.35 | 53.05 |
| IFIT1 | 8.13 | 6.88 | 9.01 | 6.77 | 40.15 |
| DMWD | 5.83 | 10.68 | 6.52 | 11.85 | 12.79 |
| PCYT2 | 9.02 | 20.36 | 6.67 | 6.84 | 21.29 |
| IFITM1 | 10.29 | 7.80 | 14.34 | 15.75 | 34.42 |
| PIP5K1C | 22.60 | 39.23 | 23.97 | 40.95 | 48.20 |
| BCL9L | 11.02 | 16.63 | 10.90 | 28.09 | 22.63 |
| MKL2 | 3.16 | 6.72 | 6.29 | 7.89 | 12.04 |
| ARAP1 | 10.23 | 23.77 | 11.96 | 29.12 | 25.09 |
| TPCN1 | 5.53 | 20.36 | 8.41 | 22.40 | 20.62 |
| KRT14 | 3.93 | 9.33 | 5.00 | 15.10 | 120.82 |
| ZACN | 15.11 | 43.36 | 19.29 | 42.15 | 52.01 |
| MAGED4B | 22.83 | 28.42 | 22.42 | 45.98 | 49.20 |
| TAF9B | 6.20 | 12.11 | 8.56 | 9.54 | 21.29 |
| PEAR1 | 35.36 | 64.61 | 34.40 | 88.15 | 85.33 |
| COL14A1 | 20.30 | 39.23 | 11.43 | 19.56 | 54.69 |
| MAPK12 | 7.61 | 12.43 | 8.64 | 9.56 | 15.70 |
| FAM83G | 7.69 | 13.78 | 3.05 | 10.06 | 16.14 |
| NOC2L | 28.46 | 64.29 | 36.74 | 49.26 | 71.24 |
| S100A4 | 33.73 | 82.69 | 29.41 | 41.78 | 92.94 |
| PLEKHG4 | 8.72 | 19.88 | 13.93 | 19.71 | 24.94 |
| STK40 | 8.20 | 12.51 | 8.18 | 19.33 | 18.31 |
| TMEM63A | 13.40 | 18.85 | 12.11 | 26.74 | 33.81 |
| IPO4 | 9.76 | 30.75 | 13.47 | 28.71 | 32.10 |
| NCOR2 | 15.85 | 57.39 | 17.40 | 47.69 | 50.81 |
| PLXNB2 | 95.84 | 183.09 | 77.31 | 198.79 | 233.33 |
| MKL1 | 7.69 | 10.84 | 4.78 | 12.90 | 16.74 |
| HRH1 | 7.54 | 12.98 | 14.61 | 11.18 | 23.60 |
| ARID5A | 6.20 | 17.74 | 4.10 | 15.89 | 15.55 |
| FLNA | 394.90 | 800.23 | 281.14 | 1128.23 | 862.16 |
| AP2A1 | 18.52 | 44.39 | 15.51 | 53.45 | 53.50 |
| METTL9 | 25.35 | 100.93 | 29.34 | 43.35 | 76.83 |
| ANXA6 | 91.10 | 198.39 | 130.42 | 155.63 | 201.05 |
| ZNF441 | 3.83 | 12.27 | 10.22 | 11.48 | 8.61 |
| IGF2R | 12.51 | 61.52 | 14.98 | 31.45 | 45.30 |
| SRC | 3.23 | 11.63 | 4.03 | 10.21 | 11.37 |
| PCNX3 | 5.31 | 14.65 | 4.86 | 10.66 | 24.57 |
| MIRLET7BHG | 6.05 | 9.10 | 3.35 | 6.99 | 12.79 |
| HTT | 16.74 | 34.08 | 18.31 | 27.04 | 39.70 |
| ENTPD6 | 25.20 | 45.89 | 15.21 | 32.50 | 70.35 |
| SERPINB2 | 9.61 | 20.12 | 13.17 | 7.37 | 75.12 |
| ATAD3A | 3.16 | 6.98 | 5.06 | 8.67 | 12.67 |
| GPAA1 | 61.86 | 190.23 | 53.06 | 167.75 | 179.50 |
| MYO1C | 91.84 | 190.38 | 76.78 | 178.14 | 202.17 |
| FCHSD1 | 9.76 | 18.61 | 7.50 | 15.89 | 23.90 |
| ZNF335 | 6.28 | 11.71 | 6.97 | 10.21 | 12.64 |
| DDRGK1 | 14.88 | 34.95 | 11.13 | 26.37 | 44.03 |
| SZT2 | 5.24 | 10.29 | 7.43 | 8.26 | 12.64 |
| GLMP | 37.67 | 65.24 | 41.20 | 44.92 | 90.55 |
| APCDD1L | 7.17 | 11.00 | 13.70 | 14.17 | 16.22 |
| TMEM184B | 32.25 | 116.63 | 24.58 | 97.43 | 116.95 |
| MT-CO1 | 3781.08 | 3700.75 | 3230.78 | 5127.57 | 8938.85 |
| DENND4B | 3.23 | 8.22 | 6.14 | 13.05 | 11.74 |
| RUSC2 | 23.42 | 40.98 | 15.21 | 50.45 | 47.01 |
| SHISA4 | 24.75 | 63.26 | 24.12 | 50.68 | 64.68 |
| SMG5 | 10.65 | 42.56 | 10.22 | 33.62 | 57.00 |
| TGM2 | 309.49 | 610.85 | 246.01 | 394.10 | 1109.92 |
| INF2 | 15.63 | 36.14 | 18.16 | 34.07 | 45.89 |
| NHSL2 | 5.53 | 12.82 | 8.64 | 7.44 | 13.76 |
| PSMB8 | 8.95 | 10.05 | 18.08 | 14.47 | 22.56 |
| SKIV2L | 12.58 | 25.99 | 8.79 | 29.81 | 30.53 |
| VARS | 16.07 | 27.97 | 15.59 | 21.50 | 35.75 |
| PRRC2A | 12.66 | 29.32 | 14.61 | 38.33 | 41.72 |
| PPP1R10 | 22.38 | 48.99 | 15.81 | 42.97 | 50.22 |
| GNL1 | 15.11 | 39.63 | 25.79 | 37.51 | 42.54 |
| AKT1S1 | 7.54 | 39.39 | 7.96 | 19.33 | 35.08 |
| DCTN1 | 91.99 | 202.44 | 71.57 | 157.42 | 222.90 |
| NYNRIN | 2.86 | 12.74 | 11.43 | 13.05 | 14.95 |
| MT-RNR2 | 15065.79 | 28113.08 | 14421.44 | 33498.52 | 42652.27 |
| CRIP1 | 4.79 | 8.99 | 4.03 | 13.57 | 9.81 |
| ATF6B | 4.87 | 12.59 | 6.82 | 13.35 | 19.13 |
| TAX1BP3 | 103.11 | 197.32 | 71.85 | 181.54 | 229.97 |
| ARHGEF28 | 2.71 | 13.62 | 10.07 | 8.79 | 17.11 |
| IFI30 | 13.96 | 18.81 | 15.37 | 13.16 | 33.79 |
| RP11-228B15.4 | 5.09 | 11.48 | 5.31 | 9.76 | 10.85 |
| MCRIP1 | 45.38 | 104.02 | 44.82 | 86.28 | 121.57 |
| MIR29A | 4.87 | 8.86 | 13.93 | 3.25 | 12.34 |
| SCAMP4 | 13.47 | 41.21 | 9.32 | 35.12 | 49.17 |
| TAPBP | 92.06 | 192.37 | 93.70 | 165.73 | 227.00 |
| RP11-196G11.6 | 6.20 | 12.59 | 4.18 | 14.10 | 12.42 |
| CYP1B1-AS1 | 8.72 | 24.16 | 11.28 | 8.11 | 21.44 |
| RBM14 | 14.22 | 39.40 | 11.54 | 42.86 | 48.16 |
| GET4 | 17.11 | 29.72 | 19.82 | 22.48 | 35.38 |
| PCDHGC3 | 44.34 | 113.19 | 44.71 | 100.26 | 131.99 |
| PLEKHO2 | 9.76 | 23.85 | 9.32 | 20.91 | 24.79 |
| C8orf58 | 5.68 | 11.81 | 6.97 | 8.11 | 12.06 |
| PEG10 | 13.77 | 19.17 | 23.82 | 11.03 | 28.07 |
| RGAG4 | 4.35 | 9.02 | 6.14 | 11.26 | 10.48 |
| AP5Z1 | 7.76 | 14.17 | 2.29 | 12.38 | 20.77 |
| FAM86DP | 12.42 | 22.40 | 11.63 | 12.75 | 25.90 |
| RP11-597D13.9 | 5.53 | 13.46 | 5.92 | 6.02 | 11.22 |
| RP11-295K3.1 | 11.69 | 33.50 | 6.45 | 21.82 | 36.40 |
| SEPP1 | 8.80 | 24.01 | 16.80 | 10.13 | 26.81 |
| RP11-834C11.4 | 13.84 | 25.12 | 15.06 | 19.48 | 33.22 |
| PCDHGA12 | 6.68 | 21.82 | 4.51 | 18.89 | 21.17 |
| PCDHGB4 | 9.49 | 26.03 | 9.32 | 18.28 | 26.73 |
| CTC-435M10.3 | 2.49 | 8.54 | 5.94 | 15.18 | 5.47 |
| RP11-286N22.8 | 5.96 | 7.47 | 13.19 | 11.05 | 12.64 |
| RP11-290L1.3 | 3.83 | 18.14 | 13.02 | 10.58 | 18.23 |
| TUBB3 | 8.55 | 27.39 | 12.17 | 16.38 | 57.01 |
| RP11-66B24.4 | 7.46 | 9.97 | 11.73 | 4.37 | 28.82 |
| RP11-343C2.11 | 4.95 | 25.55 | 10.80 | 24.21 | 24.22 |
| BOP1 | 7.98 | 19.33 | 6.45 | 10.81 | 20.02 |
| RP13-1032I1.7 | 10.80 | 23.53 | 11.05 | 20.08 | 22.70 |
| GTF2I | 31.27 | 134.68 | 39.17 | 78.01 | 115.47 |
| RP11-334E6.12 | 27.20 | 43.83 | 30.62 | 21.73 | 59.31 |
| RP11-400F19.6 | 5.09 | 10.60 | 5.01 | 12.45 | 11.89 |
| EGLN2 | 20.60 | 40.34 | 20.80 | 50.98 | 56.33 |
| RN7SL2 | 6.97 | 29.07 | 6.27 | 26.96 | 27.18 |
| CTB-147N14.6 | 4.27 | 39.07 | 3.20 | 48.51 | 27.10 |
| RN7SL1 | 15.10 | 66.17 | 17.43 | 40.93 | 38.15 |
| PCDHGB5 | 4.83 | 16.29 | 4.93 | 19.75 | 19.53 |
| AC008522.1 | 66.31 | 137.01 | 132.16 | 100.87 | 136.26 |
| AC011558.5 | 9.99 | 22.18 | 12.11 | 17.76 | 26.36 |
| RP11-2C24.3 | 10.21 | 20.20 | 8.86 | 23.07 | 25.76 |
| CH507-513H4.4 | 75.02 | 115.28 | 49.95 | 241.39 | 177.66 |
| CH507-513H4.6 | 75.02 | 115.28 | 49.95 | 241.39 | 177.66 |
| CH507-513H4.3 | 75.02 | 115.28 | 49.95 | 241.39 | 177.66 |
| ABALON | 7.17 | 24.16 | 11.66 | 20.38 | 27.85 |
| RP11-2C24.9 | 5.85 | 14.36 | 10.76 | 11.98 | 19.86 |
| AL513122.2 | 9.39 | 117.45 | 14.41 | 72.13 | 75.69 |
| POLDIP2 | 92.95 | 186.10 | 94.69 | 119.65 | 185.09 |
| ZFP64 | 10.80 | 24.32 | 12.34 | 16.72 | 18.01 |
| OSBPL5 | 7.31 | 15.76 | 5.46 | 16.12 | 13.61 |
| TDP1 | 7.46 | 15.84 | 13.55 | 7.22 | 12.34 |
| MCUR1 | 12.21 | 24.96 | 9.47 | 16.94 | 16.96 |
| FOXJ2 | 9.91 | 27.10 | 9.09 | 24.65 | 18.83 |
| TMEM260 | 10.21 | 21.47 | 12.49 | 14.25 | 17.26 |
| RNF126 | 7.17 | 14.96 | 5.24 | 12.23 | 11.74 |
| STXBP2 | 3.61 | 7.87 | 5.99 | 14.70 | 5.87 |
| ZNF343 | 5.01 | 11.72 | 6.29 | 7.66 | 7.57 |
| RAB36 | 8.65 | 22.74 | 5.09 | 14.47 | 16.81 |
| RHBDD3 | 8.65 | 18.30 | 10.98 | 15.67 | 16.22 |
| MTMR3 | 13.10 | 27.49 | 11.58 | 33.10 | 23.67 |
| BDKRB1 | 16.29 | 40.55 | 44.90 | 19.77 | 25.99 |
| C20orf24 | 17.87 | 39.55 | 6.75 | 23.22 | 33.07 |
| DNTTIP1 | 6.50 | 13.38 | 4.86 | 9.01 | 11.07 |
| MID1 | 25.57 | 53.43 | 23.97 | 17.99 | 42.84 |
| TAZ | 5.83 | 12.03 | 3.58 | 7.37 | 9.81 |
| GGA2 | 17.33 | 43.67 | 11.58 | 19.63 | 27.55 |
| SLC1A5 | 145.71 | 296.02 | 95.52 | 411.83 | 226.92 |
| MOSPD3 | 10.13 | 22.58 | 8.33 | 14.47 | 13.01 |
| AHR | 83.15 | 178.89 | 91.36 | 138.65 | 127.01 |
| KANK1 | 23.34 | 49.62 | 17.78 | 20.53 | 39.41 |
| KLF3 | 7.17 | 14.49 | 10.15 | 20.76 | 11.82 |
| APH1A | 6.72 | 13.62 | 4.33 | 12.15 | 9.88 |
| CTGF | 5992.43 | 12756.23 | 4937.79 | 11004.02 | 11750.07 |
| ELL2 | 105.12 | 234.48 | 73.53 | 110.74 | 187.78 |
| PGF | 28.69 | 65.40 | 25.94 | 63.62 | 23.08 |
| PTGIS | 13.18 | 26.54 | 12.41 | 18.14 | 23.23 |
| PEPD | 30.54 | 68.02 | 24.35 | 60.18 | 60.80 |
| ABCC10 | 9.24 | 21.63 | 2.59 | 15.52 | 17.26 |
| C16orf70 | 10.06 | 21.86 | 16.49 | 22.85 | 20.10 |
| TMX4 | 29.58 | 67.86 | 34.70 | 60.10 | 49.84 |
| ZNF426 | 12.21 | 24.48 | 26.54 | 18.36 | 17.56 |
| CHMP1A | 50.13 | 116.79 | 35.23 | 86.06 | 94.58 |
| PODNL1 | 6.28 | 13.46 | 1.38 | 15.37 | 10.55 |
| WBP2 | 67.05 | 146.93 | 52.91 | 126.60 | 134.10 |
| REEP2 | 8.28 | 18.22 | 5.01 | 12.45 | 13.76 |
| FLOT2 | 41.67 | 86.97 | 48.98 | 71.02 | 67.29 |
| FADS2 | 13.70 | 31.62 | 6.07 | 37.96 | 19.35 |
| ADAMTS7 | 13.84 | 29.99 | 9.09 | 38.83 | 25.53 |
| HLX | 8.20 | 17.66 | 7.13 | 15.22 | 12.27 |
| TJAP1 | 5.90 | 12.19 | 4.56 | 10.43 | 10.40 |
| SEMA6D | 6.35 | 16.71 | 24.20 | 10.81 | 10.70 |
| AFG3L2 | 22.60 | 52.48 | 19.59 | 40.43 | 42.61 |
| SLC16A3 | 8.80 | 29.16 | 10.30 | 25.17 | 15.70 |
| NT5C3B | 33.06 | 81.58 | 26.47 | 32.50 | 53.05 |
| PFKL | 68.68 | 169.13 | 38.10 | 177.69 | 83.62 |
| C1orf216 | 16.89 | 35.58 | 13.55 | 29.73 | 33.22 |
| OSR1 | 16.07 | 35.66 | 21.33 | 30.85 | 15.55 |
| SLC30A5 | 22.97 | 59.14 | 21.48 | 32.35 | 33.59 |
| ATXN7L1 | 3.75 | 8.54 | 4.03 | 13.95 | 6.82 |
| NCAPG2 | 7.91 | 16.71 | 13.47 | 10.21 | 9.81 |
| SH3KBP1 | 75.73 | 174.05 | 89.32 | 147.03 | 147.29 |
| TMEM47 | 144.67 | 334.72 | 144.62 | 135.21 | 177.27 |
| SLC16A2 | 28.39 | 65.16 | 25.94 | 37.36 | 48.65 |
| MTA2 | 6.13 | 12.43 | 11.05 | 10.88 | 10.78 |
| PPP4C | 50.80 | 102.60 | 59.56 | 63.92 | 91.15 |
| SEC24D | 105.57 | 212.11 | 100.43 | 133.64 | 151.17 |
| PLBD2 | 42.04 | 84.51 | 20.57 | 75.81 | 64.31 |
| TXNDC11 | 12.66 | 30.59 | 13.40 | 10.88 | 21.96 |
| PPP2R5E | 20.15 | 45.34 | 25.11 | 26.59 | 32.10 |
| SLC26A2 | 4.42 | 11.55 | 12.57 | 7.67 | 7.20 |
| CCDC117 | 17.48 | 40.82 | 15.89 | 36.32 | 24.79 |
| ST6GALNAC6 | 16.52 | 33.20 | 11.51 | 31.83 | 26.21 |
| ITGA5 | 507.92 | 1088.90 | 375.27 | 1007.50 | 920.17 |
| SPC24 | 3.46 | 7.03 | 16.27 | 5.57 | 6.38 |
| GMEB1 | 4.05 | 9.49 | 11.66 | 6.92 | 7.42 |
| KIAA1841 | 4.42 | 9.41 | 12.26 | 8.56 | 7.87 |
| IGFBP7 | 419.10 | 840.91 | 225.69 | 626.37 | 487.28 |
| STK36 | 8.13 | 16.55 | 6.45 | 17.02 | 13.91 |
| HDAC11 | 6.13 | 15.12 | 8.41 | 11.85 | 10.70 |
| NUAK2 | 8.87 | 20.75 | 10.30 | 9.31 | 16.96 |
| TPRA1 | 12.58 | 29.24 | 8.64 | 20.01 | 24.49 |
| HHIP | 34.33 | 79.68 | 233.39 | 48.13 | 60.36 |
| GPER1 | 12.06 | 30.03 | 7.58 | 20.46 | 23.45 |
| TAF3 | 10.06 | 20.28 | 12.11 | 14.25 | 15.85 |
| IKBIP | 20.75 | 45.66 | 23.67 | 27.86 | 36.95 |
| CKB | 28.39 | 57.63 | 16.72 | 49.26 | 50.37 |
| SMG8 | 18.13 | 42.80 | 18.84 | 23.10 | 29.64 |
| SRP68 | 63.04 | 127.58 | 60.69 | 89.13 | 114.49 |
| BDKRB2 | 6.42 | 20.67 | 11.36 | 16.64 | 10.63 |
| ATXN2L | 12.95 | 29.40 | 13.55 | 27.19 | 22.85 |
| TSPAN5 | 13.84 | 28.37 | 14.23 | 14.62 | 17.93 |
| TM4SF20 | 5.83 | 14.49 | 2.44 | 6.84 | 7.72 |
| PFKFB3 | 21.56 | 56.28 | 16.95 | 49.48 | 31.58 |
| USP19 | 7.83 | 19.09 | 6.22 | 16.72 | 14.73 |
| PDE3A | 15.48 | 33.60 | 33.04 | 27.86 | 17.86 |
| PHF8 | 4.94 | 15.84 | 5.92 | 11.18 | 9.66 |
| HSPB7 | 482.17 | 1036.40 | 255.83 | 1043.63 | 845.61 |
| RPS6KB2 | 34.77 | 71.91 | 22.92 | 48.81 | 66.62 |
| A2M | 7.31 | 18.38 | 5.77 | 23.30 | 9.28 |
| BET1L | 38.18 | 77.38 | 20.27 | 68.48 | 69.06 |
| ALS2CL | 5.76 | 13.22 | 3.20 | 10.88 | 11.00 |
| KLHL11 | 7.09 | 15.84 | 8.11 | 13.72 | 13.53 |
| LACC1 | 7.69 | 15.84 | 11.05 | 7.96 | 9.43 |
| SEPHS2 | 20.67 | 59.22 | 12.19 | 32.95 | 38.73 |
| S1PR5 | 4.72 | 12.43 | 10.30 | 12.30 | 7.27 |
| CHRM2 | 10.36 | 22.26 | 19.21 | 25.17 | 9.36 |
| LDOC1 | 41.15 | 97.60 | 43.39 | 77.38 | 77.51 |
| VMO1 | 5.31 | 11.16 | 11.51 | 5.27 | 10.10 |
| CRELD2 | 19.78 | 39.63 | 16.27 | 29.06 | 39.03 |
| FAM43A | 6.13 | 14.41 | 4.25 | 13.65 | 10.33 |
| ADAMTSL5 | 5.83 | 14.89 | 6.07 | 16.72 | 10.40 |
| SPIN4 | 5.16 | 12.59 | 7.13 | 7.07 | 7.12 |
| IFT140 | 5.61 | 12.19 | 4.25 | 10.73 | 10.10 |
| FAM122A | 10.13 | 21.55 | 8.94 | 12.53 | 16.67 |
| UBQLN2 | 67.13 | 138.84 | 60.31 | 118.08 | 105.91 |
| FAM180A | 41.67 | 97.52 | 62.80 | 53.89 | 68.19 |
| SLC2A10 | 32.10 | 71.35 | 36.44 | 46.26 | 63.79 |
| ATG9A | 45.40 | 106.48 | 33.93 | 102.56 | 89.26 |
| ADGRG1 | 14.88 | 45.73 | 10.68 | 24.65 | 24.42 |
| CDPF1 | 4.87 | 9.89 | 6.90 | 14.47 | 9.43 |
| ZNF611 | 7.39 | 16.68 | 13.07 | 12.90 | 10.63 |
| MBNL1-AS1 | 36.18 | 75.79 | 42.48 | 54.34 | 64.98 |
| GSN-AS1 | 3.16 | 10.29 | 3.27 | 13.80 | 5.18 |
| PXN-AS1 | 8.20 | 17.66 | 9.32 | 12.83 | 16.29 |
| RP11-498C9.3 | 13.32 | 28.53 | 17.10 | 23.67 | 25.84 |
| PCDHGA4 | 3.90 | 16.66 | 6.57 | 17.09 | 5.11 |
| CTD-2541J13.2 | 12.88 | 37.41 | 14.00 | 20.91 | 23.38 |
| BIVM-ERCC5 | 5.70 | 12.57 | 9.22 | 15.86 | 10.73 |
| NATD1 | 4.64 | 10.60 | 1.99 | 11.11 | 7.27 |
| SYNRG | 16.14 | 33.05 | 27.52 | 24.20 | 31.88 |
| RP4-614O4.13 | 12.21 | 24.87 | 22.46 | 17.09 | 17.41 |
| SARM1 | 8.43 | 12.74 | 6.45 | 17.69 | 12.56 |
| PLXND1 | 34.55 | 59.85 | 25.41 | 88.68 | 54.39 |
| EHD3 | 17.11 | 31.46 | 18.08 | 36.32 | 19.65 |
| BAK1 | 28.02 | 46.45 | 11.89 | 60.10 | 51.63 |
| ZNF800 | 7.17 | 13.14 | 8.03 | 18.74 | 10.85 |
| ZNF76 | 11.02 | 15.12 | 10.60 | 24.05 | 15.10 |
| TBC1D22B | 9.02 | 11.95 | 7.81 | 18.59 | 10.40 |
| FRY | 6.42 | 5.92 | 4.78 | 13.80 | 8.39 |
| ARHGEF10L | 14.22 | 23.05 | 6.90 | 32.20 | 26.51 |
| COL5A3 | 5.83 | 7.99 | 6.60 | 22.03 | 9.13 |
| PPP1R15A | 78.03 | 146.85 | 104.36 | 156.45 | 135.44 |
| ITPR3 | 14.29 | 22.90 | 23.82 | 44.02 | 27.55 |
| MYH9 | 486.48 | 770.41 | 320.50 | 1027.32 | 852.25 |
| MTHFSD | 4.64 | 8.14 | 4.86 | 13.13 | 6.23 |
| SLC7A5 | 24.16 | 26.46 | 16.34 | 63.17 | 47.68 |
| CORO2B | 4.12 | 6.96 | 6.90 | 15.22 | 7.05 |
| MAN2B1 | 9.47 | 17.66 | 4.94 | 19.93 | 14.75 |
| NUCB1 | 105.12 | 207.67 | 99.52 | 222.05 | 186.21 |
| OLFM2 | 14.73 | 14.96 | 8.94 | 35.34 | 5.41 |
| PLD3 | 129.68 | 252.08 | 76.25 | 265.66 | 229.46 |
| NUMBL | 6.13 | 10.76 | 4.93 | 12.98 | 11.07 |
| CXCL12 | 163.08 | 279.36 | 166.00 | 587.32 | 233.18 |
| LZTS2 | 11.10 | 19.09 | 8.64 | 22.70 | 21.59 |
| TRIM16L | 8.98 | 9.21 | 6.31 | 26.42 | 5.15 |
| COL1A1 | 7807.86 | 9679.95 | 2890.88 | 22168.39 | 8135.61 |
| PHF12 | 7.39 | 12.51 | 5.99 | 14.92 | 8.24 |
| WHSC1 | 7.39 | 9.97 | 15.21 | 16.94 | 14.58 |
| TMEM109 | 76.70 | 144.86 | 83.73 | 157.87 | 141.63 |
| ICK | 10.13 | 14.96 | 14.68 | 22.63 | 15.55 |
| PAPD7 | 7.24 | 6.96 | 12.94 | 15.52 | 9.36 |
| PDGFRB | 65.79 | 119.80 | 63.18 | 214.57 | 114.56 |
| ABCB6 | 15.68 | 28.92 | 19.61 | 32.16 | 25.11 |
| ARHGEF2 | 27.13 | 52.71 | 23.82 | 79.33 | 36.27 |
| NID1 | 51.99 | 57.08 | 34.78 | 112.32 | 49.10 |
| HS1BP3 | 6.20 | 8.46 | 4.41 | 13.65 | 11.15 |
| LRP1 | 291.75 | 475.72 | 130.72 | 754.36 | 471.55 |
| SNAI1 | 5.98 | 10.84 | 2.14 | 14.40 | 10.85 |
| ARMC7 | 5.83 | 9.26 | 5.69 | 11.93 | 9.28 |
| MIF4GD | 7.46 | 11.40 | 6.29 | 15.22 | 11.22 |
| PLD2 | 6.05 | 6.96 | 3.42 | 12.90 | 9.06 |
| LGALS3 | 79.96 | 116.63 | 49.13 | 163.18 | 128.06 |
| EEFSEC | 10.28 | 17.26 | 11.81 | 21.35 | 17.86 |
| NES | 7.54 | 11.40 | 7.88 | 15.89 | 14.88 |
| IGHMBP2 | 9.02 | 17.11 | 8.86 | 19.63 | 15.85 |
| BTBD2 | 7.54 | 11.55 | 7.96 | 15.82 | 13.68 |
| IL18BP | 9.09 | 10.84 | 7.35 | 18.29 | 13.31 |
| ITGA11 | 227.34 | 384.04 | 103.15 | 497.26 | 450.67 |
| MFGE8 | 503.17 | 630.52 | 270.79 | 1033.01 | 629.99 |
| TGFB1I1 | 67.42 | 131.62 | 57.59 | 149.94 | 133.35 |
| GID4 | 4.72 | 6.96 | 10.30 | 11.33 | 5.63 |
| ATP8B2 | 10.50 | 14.25 | 7.50 | 23.45 | 17.26 |
| SOGA1 | 23.12 | 39.55 | 17.17 | 53.82 | 39.85 |
| ME3 | 5.09 | 9.89 | 7.88 | 11.63 | 8.91 |
| ACAN | 377.17 | 712.60 | 339.76 | 974.36 | 429.13 |
| IGF2BP1 | 6.79 | 8.30 | 4.56 | 16.34 | 7.27 |
| C1R | 17.70 | 16.15 | 8.49 | 45.22 | 29.12 |
| ARHGAP35 | 18.45 | 34.63 | 11.43 | 42.90 | 33.37 |
| ZNF362 | 5.98 | 11.08 | 5.84 | 13.20 | 8.46 |
| CBS | 21.93 | 25.35 | 18.38 | 47.42 | 23.38 |
| G6PD | 11.17 | 16.23 | 11.43 | 34.07 | 17.04 |
| CXCR5 | 8.65 | 10.84 | 11.13 | 17.39 | 15.17 |
| ATF3 | 9.84 | 11.48 | 11.96 | 21.73 | 19.13 |
| FBLN2 | 7.54 | 8.30 | 4.78 | 25.24 | 14.06 |
| COL1A2 | 4892.52 | 5126.17 | 2283.92 | 11468.41 | 4686.59 |
| FAM102A | 7.24 | 9.10 | 3.73 | 18.96 | 10.25 |
| CERCAM | 170.28 | 281.82 | 103.15 | 357.60 | 253.69 |
| ULK4 | 7.39 | 7.99 | 4.93 | 15.07 | 10.18 |
| TNXB | 5.76 | 8.22 | 3.12 | 17.39 | 7.94 |
| BMP1 | 83.75 | 82.93 | 39.46 | 169.84 | 110.98 |
| THBS3 | 13.84 | 22.10 | 11.13 | 29.43 | 24.79 |
| FOS | 140.52 | 186.66 | 179.00 | 299.18 | 201.87 |
| GAA | 15.63 | 27.81 | 8.18 | 34.37 | 28.07 |
| LAMB2 | 113.58 | 209.58 | 87.51 | 278.31 | 196.80 |
| RELA | 36.40 | 68.49 | 25.56 | 78.50 | 68.41 |
| VANGL1 | 19.41 | 28.45 | 20.50 | 39.01 | 24.27 |
| SLC19A1 | 14.73 | 13.46 | 9.47 | 31.98 | 13.16 |
| CMKLR1 | 8.50 | 6.32 | 10.83 | 18.06 | 4.29 |
| NR1D2 | 7.54 | 7.11 | 9.54 | 17.39 | 8.61 |
| DES | 7.61 | 13.93 | 6.45 | 16.49 | 12.94 |
| ACSF3 | 10.50 | 16.71 | 8.41 | 21.95 | 18.75 |
| TOP3A | 5.68 | 10.37 | 7.58 | 12.23 | 8.69 |
| RNF135 | 15.33 | 26.38 | 11.05 | 31.68 | 24.34 |
| BGN | 341.33 | 399.91 | 196.68 | 779.49 | 454.18 |
| TMEM198B | 6.28 | 9.10 | 8.33 | 13.05 | 8.17 |
| COL18A1 | 18.22 | 13.78 | 13.25 | 45.74 | 12.86 |
| AC138969.4 | 13.05 | 12.24 | 14.61 | 34.52 | 21.15 |
| NELFA | 5.68 | 7.99 | 4.63 | 12.53 | 7.27 |
| PARPBP | 4.57 | 9.02 | 12.41 | 10.06 | 6.75 |
| KLHDC8B | 10.50 | 15.44 | 5.69 | 22.03 | 20.54 |
| GPAT2 | 18.47 | 34.37 | 27.50 | 38.15 | 35.77 |
| LDLRAD2 | 47.83 | 67.62 | 40.82 | 100.80 | 63.64 |
| KCTD21 | 11.91 | 19.09 | 9.69 | 24.57 | 12.12 |
| SLC6A9 | 16.37 | 23.29 | 13.62 | 48.66 | 17.26 |
| C6orf106 | 13.32 | 24.32 | 9.32 | 28.61 | 22.18 |
| ZNF71 | 5.98 | 10.05 | 5.84 | 12.38 | 9.13 |
| GPATCH3 | 6.94 | 8.70 | 7.58 | 15.67 | 9.58 |
| PLXNB3 | 5.61 | 8.86 | 5.92 | 13.05 | 7.72 |
| MT-ND3 | 21.27 | 25.35 | 25.48 | 55.17 | 37.32 |
| SREBF2 | 18.22 | 28.29 | 15.21 | 37.74 | 32.77 |
| GP1BB | 5.37 | 6.08 | 3.30 | 12.94 | 7.86 |
| TCTN1 | 29.43 | 29.64 | 16.19 | 60.10 | 35.31 |
| KCTD11 | 16.66 | 28.53 | 2.44 | 38.26 | 25.02 |
| AP1G2 | 5.90 | 11.71 | 6.52 | 12.00 | 9.88 |
| TWF2 | 21.75 | 37.72 | 35.38 | 53.59 | 37.47 |
| CTD-2033D15.3 | 6.13 | 10.44 | 5.01 | 17.46 | 11.30 |
| RP3-394A18.1 | 9.99 | 19.56 | 7.50 | 24.05 | 15.47 |
| PAGR1 | 8.28 | 14.01 | 6.82 | 16.61 | 12.49 |
| RP11-949J7.8 | 5.90 | 6.77 | 7.26 | 13.69 | 8.16 |
| TFPI | 323.22 | 278.41 | 683.36 | 188.92 | 230.87 |
| RAD51 | 4.42 | 5.53 | 12.94 | 5.57 | 8.76 |
| DCBLD2 | 244.63 | 282.14 | 702.47 | 157.87 | 277.32 |
| DDX3Y | 5.16 | 10.05 | 12.49 | 6.47 | 9.81 |
| TGFBR3 | 15.18 | 21.31 | 30.70 | 19.04 | 20.32 |
| MEF2C | 6.94 | 10.76 | 15.06 | 11.33 | 8.24 |
| BIRC5 | 8.06 | 12.67 | 27.98 | 8.49 | 15.70 |
| PDCD7 | 6.05 | 6.48 | 13.32 | 4.97 | 6.53 |
| CDKN3 | 9.84 | 9.26 | 19.82 | 5.50 | 8.84 |
| VAC14 | 6.79 | 11.95 | 13.85 | 13.50 | 10.92 |
| ITGB8 | 6.28 | 9.65 | 12.57 | 5.57 | 8.17 |
| APBA1 | 16.41 | 16.47 | 33.57 | 11.18 | 20.02 |
| LIN7A | 14.14 | 22.66 | 31.15 | 18.21 | 17.41 |
| SH2B3 | 21.86 | 22.50 | 45.81 | 27.71 | 31.35 |
| CDCA3 | 6.20 | 10.44 | 12.49 | 6.24 | 6.38 |
| HECA | 14.22 | 9.10 | 28.66 | 8.64 | 15.17 |
| GPD2 | 17.55 | 30.43 | 38.63 | 16.72 | 31.73 |
| CCDC88A | 19.93 | 29.56 | 41.27 | 28.54 | 27.92 |
| MLPH | 9.17 | 12.67 | 18.46 | 10.73 | 17.86 |
| ADGRL2 | 32.69 | 44.15 | 69.23 | 47.09 | 38.88 |
| SSX2IP | 9.32 | 16.31 | 18.91 | 7.52 | 13.61 |
| CDC20 | 4.79 | 7.11 | 16.04 | 5.87 | 8.84 |
| SGIP1 | 32.32 | 43.28 | 67.72 | 33.55 | 46.71 |
| PTGFR | 6.94 | 11.24 | 17.10 | 9.16 | 9.28 |
| KCNJ2 | 9.17 | 9.41 | 30.70 | 3.92 | 10.85 |
| AKAP12 | 156.77 | 168.50 | 349.89 | 122.64 | 201.72 |
| RIN2 | 101.93 | 91.26 | 226.14 | 50.53 | 89.51 |
| TBC1D8B | 20.30 | 26.15 | 43.09 | 22.63 | 22.11 |
| CCNB1 | 20.00 | 25.83 | 54.27 | 15.15 | 32.47 |
| NT5E | 237.58 | 343.60 | 554.17 | 285.34 | 338.69 |
| SP110 | 8.65 | 9.65 | 17.70 | 11.55 | 16.89 |
| NEK3 | 5.01 | 5.85 | 12.04 | 7.89 | 4.96 |
| SPRY2 | 11.54 | 10.68 | 24.50 | 7.52 | 7.72 |
| PRPF4 | 19.48 | 28.21 | 49.74 | 26.59 | 32.77 |
| NUSAP1 | 6.13 | 9.73 | 22.84 | 5.05 | 7.27 |
| KIF23 | 16.96 | 26.78 | 43.46 | 16.49 | 24.87 |
| STAMBPL1 | 10.21 | 10.29 | 27.60 | 6.62 | 15.77 |
| AOX1 | 14.59 | 18.30 | 32.43 | 10.36 | 15.62 |
| BARD1 | 11.54 | 9.41 | 25.86 | 11.85 | 9.28 |
| SENP7 | 9.24 | 10.76 | 20.42 | 12.00 | 8.02 |
| FGF5 | 63.42 | 119.65 | 165.02 | 89.87 | 123.21 |
| FGD4 | 48.94 | 51.84 | 98.92 | 44.39 | 50.07 |
| WDFY2 | 17.33 | 24.80 | 54.49 | 28.39 | 34.41 |
| POU2F1 | 5.83 | 10.68 | 12.11 | 9.61 | 10.78 |
| NCEH1 | 65.27 | 98.23 | 179.60 | 66.39 | 99.65 |
| ARHGAP18 | 15.40 | 16.39 | 35.38 | 13.87 | 20.24 |
| MRRF | 9.69 | 15.60 | 23.29 | 17.91 | 17.49 |
| SLX4IP | 22.01 | 19.56 | 50.11 | 17.39 | 13.53 |
| ORAOV1 | 43.08 | 75.16 | 92.04 | 48.13 | 74.75 |
| HMGA2 | 17.93 | 20.99 | 44.82 | 15.00 | 22.63 |
| FBXO36 | 7.02 | 8.14 | 15.59 | 9.46 | 9.43 |
| FMN2 | 37.29 | 45.58 | 112.74 | 50.08 | 36.65 |
| FCHO2 | 29.21 | 21.07 | 60.24 | 27.71 | 26.43 |
| EMSY | 7.09 | 10.76 | 14.76 | 12.00 | 10.10 |
| RACGAP1 | 17.55 | 20.99 | 43.69 | 18.06 | 27.92 |
| DIRAS3 | 6.05 | 6.64 | 24.80 | 1.98 | 9.13 |
| WDR43 | 57.18 | 55.65 | 115.16 | 56.96 | 64.38 |
| MAD2L1 | 5.61 | 10.37 | 15.36 | 10.58 | 11.15 |
| ESM1 | 11.84 | 13.46 | 49.81 | 17.54 | 13.24 |
| TMEM200A | 131.76 | 210.13 | 296.10 | 97.28 | 178.46 |
| PTTG1 | 10.65 | 15.84 | 27.83 | 12.00 | 13.83 |
| SERPINB7 | 7.46 | 9.89 | 15.74 | 9.54 | 12.34 |
| FEN1 | 10.80 | 12.27 | 22.92 | 12.38 | 13.31 |
| IL7R | 16.37 | 29.79 | 46.86 | 17.61 | 27.92 |
| ADRB2 | 7.02 | 10.68 | 19.67 | 3.10 | 7.27 |
| ZNF439 | 6.42 | 10.29 | 15.06 | 8.04 | 5.78 |
| ZNF562 | 31.80 | 31.86 | 64.92 | 19.48 | 32.70 |
| ZWILCH | 15.11 | 23.69 | 38.25 | 18.89 | 23.15 |
| PLEKHM3 | 9.24 | 14.41 | 22.01 | 14.70 | 13.09 |
| BTBD9 | 25.72 | 31.70 | 53.81 | 23.52 | 30.09 |
| FOCAD | 50.50 | 97.84 | 101.03 | 77.83 | 89.06 |
| ACADSB | 22.97 | 26.62 | 47.09 | 24.94 | 18.16 |
| ZMYM1 | 9.39 | 11.32 | 19.52 | 11.33 | 7.87 |
| RP11-366L20.2 | 22.38 | 25.04 | 62.05 | 18.36 | 25.54 |
| PELI1 | 6.20 | 9.81 | 15.13 | 8.49 | 7.79 |
| VEPH1 | 17.11 | 25.12 | 48.98 | 8.11 | 24.64 |
| MAP3K5 | 4.72 | 8.46 | 12.19 | 6.54 | 7.72 |
| CD2AP | 17.55 | 22.74 | 39.38 | 20.01 | 22.85 |
| LPAR1 | 88.57 | 147.32 | 185.04 | 87.26 | 130.59 |
| ZNF652 | 23.05 | 30.03 | 50.49 | 32.05 | 23.38 |
| ZNF883 | 5.31 | 8.62 | 11.81 | 8.49 | 9.21 |
| AC016722.4 | 7.31 | 6.48 | 14.76 | 7.14 | 7.94 |
| AC093616.4 | 5.98 | 10.24 | 12.25 | 8.64 | 9.87 |
| RP11-342D11.2 | 6.20 | 7.75 | 12.94 | 6.47 | 6.60 |
| STON1 | 8.72 | 11.38 | 19.52 | 10.92 | 10.78 |
| BDNF-AS | 7.39 | 9.57 | 14.83 | 6.77 | 8.24 |
| RP11-283I3.6 | 9.84 | 6.96 | 20.27 | 6.54 | 8.91 |
| RP11-159D12.2 | 7.30 | 6.61 | 17.35 | 7.75 | 10.27 |
| CTC-425F1.4 | 15.85 | 9.73 | 33.04 | 9.83 | 9.81 |
| ZNF2 | 8.87 | 8.62 | 17.85 | 8.86 | 10.03 |
| FAM76A | 36.33 | 25.83 | 39.38 | 22.33 | 16.37 |
| RABGAP1 | 14.66 | 8.14 | 6.67 | 20.60 | 7.12 |
| DCN | 1292.82 | 748.68 | 985.40 | 1156.88 | 554.91 |
| CLK1 | 38.26 | 21.79 | 33.95 | 34.15 | 17.93 |
| HSD17B6 | 47.39 | 29.79 | 28.73 | 19.93 | 20.77 |
| ZCCHC8 | 16.52 | 7.91 | 19.74 | 12.23 | 7.72 |
| CUL3 | 32.10 | 14.33 | 14.98 | 18.89 | 15.03 |
| HHAT | 24.16 | 13.06 | 7.13 | 15.15 | 10.18 |
| SPAG4 | 15.48 | 9.10 | 8.49 | 11.55 | 2.87 |
| PDK3 | 27.80 | 42.09 | 23.75 | 44.02 | 12.42 |
| CLTCL1 | 28.02 | 17.11 | 18.00 | 22.78 | 13.98 |
| ASNS | 143.34 | 71.27 | 124.60 | 166.47 | 62.37 |
| ING3 | 48.20 | 18.06 | 36.14 | 23.82 | 19.72 |
| P4HA2 | 807.80 | 641.15 | 639.47 | 659.43 | 382.15 |
| PTGS2 | 42.64 | 28.37 | 16.04 | 25.69 | 16.07 |
| TIMM21 | 37.81 | 22.90 | 38.93 | 28.09 | 17.78 |
| C1QTNF3 | 24.53 | 11.48 | 4.25 | 37.44 | 5.41 |
| SERTAD4 | 53.47 | 28.53 | 43.16 | 52.10 | 20.24 |
| TASP1 | 27.13 | 14.81 | 22.24 | 21.50 | 12.71 |
| ZNF302 | 45.09 | 29.40 | 31.15 | 28.61 | 21.74 |
| PHGDH | 89.02 | 73.02 | 59.48 | 133.34 | 40.23 |
| UPRT | 42.12 | 24.16 | 27.07 | 26.14 | 15.25 |
| ZNF184 | 13.99 | 9.65 | 9.32 | 4.75 | 4.29 |
| SCD | 94.21 | 147.64 | 75.72 | 135.58 | 45.67 |
| ABLIM1 | 13.32 | 9.81 | 9.17 | 8.64 | 6.23 |
| VCPKMT | 16.14 | 10.52 | 16.87 | 10.96 | 6.30 |
| SOS2 | 16.44 | 8.38 | 10.98 | 11.18 | 8.09 |
| SIX4 | 9.24 | 10.13 | 10.37 | 11.26 | 4.21 |
| PGK1 | 1803.00 | 1468.05 | 1377.80 | 1635.78 | 486.01 |
| DGKH | 13.25 | 7.03 | 9.32 | 11.55 | 5.85 |
| CCP110 | 17.41 | 14.41 | 17.70 | 12.23 | 7.42 |
| SLC30A4 | 171.24 | 96.97 | 188.14 | 88.68 | 81.83 |
| GDAP1 | 9.69 | 13.30 | 13.93 | 7.44 | 3.84 |
| DCTN6 | 93.17 | 52.40 | 76.93 | 52.77 | 46.19 |
| BNIP3L | 711.18 | 580.08 | 777.64 | 612.16 | 322.06 |
| HBP1 | 266.08 | 145.02 | 238.83 | 144.03 | 96.15 |
| TTC26 | 23.42 | 15.68 | 19.21 | 16.12 | 9.13 |
| PLEKHA8 | 12.29 | 6.72 | 7.16 | 5.57 | 6.00 |
| LSM5 | 30.10 | 14.09 | 28.73 | 16.42 | 14.13 |
| ECM2 | 34.62 | 19.56 | 8.49 | 42.75 | 13.31 |
| ALDOC | 45.31 | 51.60 | 33.04 | 57.71 | 6.53 |
| KLHL5 | 25.49 | 11.32 | 22.69 | 10.73 | 10.70 |
| CEP126 | 9.99 | 9.02 | 11.73 | 11.93 | 4.74 |
| MAGOHB | 29.21 | 19.33 | 27.60 | 20.98 | 11.30 |
| TPI1 | 3778.24 | 3251.18 | 3241.94 | 3346.85 | 1559.22 |
| ENO2 | 70.98 | 69.76 | 67.64 | 85.16 | 19.27 |
| C6orf62 | 1163.40 | 579.69 | 1492.33 | 509.75 | 493.99 |
| QKI | 76.40 | 42.96 | 74.06 | 58.53 | 36.13 |
| HMGCS1 | 46.72 | 22.50 | 49.51 | 23.97 | 19.35 |
| CLK4 | 29.65 | 19.01 | 32.51 | 21.43 | 14.50 |
| CRBN | 75.07 | 49.38 | 76.03 | 54.34 | 32.32 |
| FAM162A | 106.16 | 49.22 | 90.30 | 68.71 | 25.24 |
| SNX4 | 62.60 | 36.30 | 40.29 | 38.63 | 30.38 |
| SOS1 | 31.51 | 15.12 | 24.05 | 24.05 | 15.55 |
| PADI2 | 50.80 | 25.04 | 17.63 | 58.01 | 10.48 |
| DDX59 | 24.60 | 12.98 | 14.76 | 17.24 | 10.25 |
| UBE2B | 112.54 | 59.77 | 89.40 | 64.67 | 53.35 |
| MXI1 | 23.19 | 11.87 | 15.29 | 13.95 | 6.60 |
| EXOSC8 | 65.20 | 23.77 | 71.42 | 39.31 | 24.12 |
| RDH10 | 39.37 | 20.75 | 21.93 | 17.39 | 19.42 |
| P4HA1 | 484.77 | 525.36 | 490.56 | 467.79 | 186.06 |
| B9D2 | 12.14 | 7.11 | 7.81 | 6.32 | 5.41 |
| MCEE | 16.66 | 8.86 | 14.91 | 9.24 | 7.57 |
| EIF2S2 | 548.48 | 239.48 | 558.99 | 285.19 | 236.11 |
| CHN1 | 20.15 | 9.81 | 10.68 | 20.31 | 8.24 |
| RPAIN | 21.56 | 10.76 | 17.48 | 14.92 | 10.63 |
| EAPP | 74.10 | 46.69 | 69.23 | 50.23 | 36.05 |
| PHF10 | 18.89 | 7.27 | 10.90 | 12.08 | 8.76 |
| ENOSF1 | 23.42 | 11.95 | 23.60 | 15.37 | 8.39 |
| PER2 | 22.16 | 9.65 | 22.16 | 8.86 | 8.31 |
| NASP | 26.24 | 16.39 | 27.37 | 21.65 | 11.97 |
| LPIN3 | 101.93 | 49.70 | 110.93 | 47.31 | 38.21 |
| TSPAN2 | 24.68 | 12.90 | 4.41 | 25.09 | 6.97 |
| GSTM3 | 510.59 | 267.94 | 519.42 | 238.66 | 243.62 |
| NGF | 64.90 | 31.06 | 43.46 | 29.88 | 25.09 |
| NREP | 543.47 | 201.01 | 160.19 | 537.73 | 160.71 |
| PSAT1 | 435.64 | 268.34 | 326.54 | 485.21 | 168.47 |
| CD36 | 1706.46 | 1080.97 | 1160.60 | 595.70 | 524.04 |
| TES | 359.36 | 203.87 | 282.35 | 266.79 | 152.96 |
| EGLN1 | 41.60 | 31.14 | 29.87 | 27.94 | 17.56 |
| ANKRD36 | 14.17 | 10.60 | 9.62 | 8.67 | 7.06 |
| SKIL | 326.56 | 163.98 | 440.77 | 189.36 | 140.81 |
| THAP12 | 20.82 | 7.99 | 11.81 | 14.62 | 10.40 |
| FAM117B | 11.62 | 6.64 | 14.15 | 5.72 | 3.54 |
| COX17 | 23.19 | 13.38 | 21.03 | 16.19 | 9.66 |
| ETFBKMT | 15.40 | 8.14 | 7.96 | 7.44 | 7.05 |
| AMIGO2 | 97.11 | 44.70 | 62.05 | 53.37 | 26.88 |
| TMTC3 | 358.32 | 206.88 | 424.30 | 178.07 | 170.41 |
| ARRDC4 | 16.37 | 9.73 | 6.90 | 10.36 | 4.36 |
| CDH13 | 410.56 | 168.81 | 359.56 | 335.38 | 163.40 |
| NARF | 75.66 | 53.43 | 63.18 | 55.54 | 35.16 |
| ITGB3BP | 17.18 | 11.16 | 17.93 | 12.23 | 6.53 |
| CRABP2 | 29.72 | 22.26 | 19.82 | 28.31 | 11.74 |
| POGZ | 20.52 | 16.71 | 11.51 | 26.67 | 9.36 |
| KCNH1 | 14.44 | 10.21 | 7.35 | 24.05 | 5.63 |
| PPFIA4 | 13.70 | 9.10 | 4.10 | 10.73 | 4.21 |
| ETAA1 | 24.31 | 12.74 | 20.04 | 14.70 | 11.45 |
| MEIS1 | 62.82 | 28.45 | 84.49 | 34.07 | 30.24 |
| TRPC1 | 16.00 | 11.24 | 14.83 | 8.79 | 7.49 |
| CORIN | 86.49 | 54.14 | 41.65 | 105.73 | 40.23 |
| GIN1 | 13.40 | 8.30 | 13.09 | 8.64 | 5.71 |
| BDP1 | 20.52 | 16.00 | 23.60 | 20.68 | 9.88 |
| PRSS35 | 11.47 | 8.54 | 6.29 | 11.03 | 4.44 |
| RNF217 | 32.92 | 18.14 | 33.95 | 26.37 | 13.83 |
| SHPRH | 19.86 | 8.86 | 10.37 | 13.13 | 6.60 |
| TMEM168 | 70.69 | 38.68 | 65.37 | 57.04 | 35.08 |
| CASK | 17.48 | 9.65 | 14.00 | 9.39 | 8.61 |
| VLDLR | 18.82 | 7.91 | 13.09 | 11.03 | 5.26 |
| ASTN2 | 11.32 | 6.16 | 6.67 | 11.18 | 5.03 |
| SLC7A11 | 85.53 | 29.00 | 52.76 | 108.28 | 26.36 |
| DPYSL4 | 11.77 | 9.18 | 4.33 | 9.54 | 2.80 |
| KCNE4 | 320.99 | 251.05 | 176.88 | 286.53 | 155.79 |
| PDK1 | 86.94 | 76.11 | 87.96 | 76.26 | 27.85 |
| TADA1 | 20.52 | 14.01 | 18.16 | 11.63 | 7.87 |
| XRCC4 | 24.46 | 10.21 | 17.10 | 13.57 | 10.33 |
| CDYL | 24.16 | 11.24 | 22.01 | 18.44 | 9.88 |
| ING1 | 13.84 | 7.75 | 14.23 | 8.64 | 6.53 |
| RMND5A | 64.97 | 27.65 | 39.01 | 25.39 | 21.21 |
| ANGPT1 | 98.14 | 65.08 | 64.47 | 76.78 | 48.73 |
| JAM2 | 37.74 | 31.70 | 18.16 | 11.03 | 11.37 |
| AGPAT5 | 40.86 | 20.67 | 30.62 | 27.11 | 10.18 |
| MAP3K7CL | 198.33 | 63.42 | 93.86 | 88.45 | 66.02 |
| FBXO32 | 89.83 | 53.27 | 31.83 | 65.79 | 44.10 |
| MITD1 | 20.60 | 12.82 | 11.36 | 11.55 | 10.25 |
| PAXBP1 | 18.15 | 8.30 | 15.36 | 9.61 | 8.24 |
| STC1 | 186.30 | 121.31 | 180.96 | 78.43 | 21.14 |
| ZNF761 | 42.64 | 21.48 | 50.72 | 28.29 | 19.92 |
| ICA1L | 13.32 | 7.27 | 7.81 | 6.17 | 6.15 |
| KIAA1143 | 31.80 | 21.55 | 18.08 | 25.43 | 13.16 |
| INTU | 28.17 | 20.12 | 23.97 | 20.98 | 13.83 |
| PITX2 | 104.01 | 65.48 | 69.76 | 61.30 | 40.08 |
| C4orf3 | 565.14 | 335.03 | 509.07 | 424.10 | 221.33 |
| PRSS12 | 77.00 | 49.30 | 31.15 | 61.82 | 23.90 |
| SCRG1 | 133.76 | 50.81 | 77.69 | 82.62 | 29.49 |
| MAP9 | 25.72 | 19.49 | 18.84 | 24.12 | 11.60 |
| STARD4 | 158.25 | 109.34 | 117.28 | 114.26 | 65.80 |
| F2RL2 | 22.08 | 36.69 | 22.76 | 29.43 | 7.49 |
| SLC35A1 | 78.01 | 43.59 | 60.56 | 44.30 | 36.88 |
| GEM | 24.01 | 11.71 | 18.38 | 11.55 | 11.45 |
| ABCA1 | 44.12 | 17.03 | 46.86 | 26.81 | 17.11 |
| PTER | 13.47 | 10.68 | 19.97 | 15.44 | 5.41 |
| FBN1 | 1373.56 | 802.45 | 796.38 | 1338.88 | 679.57 |
| DPCD | 61.11 | 39.79 | 52.53 | 39.76 | 28.89 |
| CYB5R2 | 28.91 | 14.17 | 16.95 | 20.16 | 12.79 |
| VKORC1 | 433.64 | 282.09 | 275.81 | 309.42 | 209.33 |
| ZNF701 | 16.04 | 7.51 | 11.50 | 3.85 | 5.71 |
| PPP1R14A | 270.53 | 115.44 | 201.28 | 175.60 | 118.14 |
| SPINT2 | 60.52 | 21.47 | 33.87 | 27.86 | 27.48 |
| DNAJC21 | 24.38 | 18.69 | 23.67 | 16.87 | 11.89 |
| TET2 | 16.29 | 8.22 | 10.68 | 10.13 | 6.75 |
| ANKRD49 | 29.80 | 15.76 | 29.34 | 21.28 | 13.31 |
| ZNF354A | 18.55 | 15.47 | 20.24 | 21.04 | 7.25 |
| SCN9A | 150.09 | 97.72 | 76.00 | 152.78 | 72.05 |
| RNF150 | 11.77 | 6.56 | 3.35 | 11.63 | 3.62 |
| CHRNB1 | 11.84 | 5.69 | 7.58 | 7.96 | 5.11 |
| INSR | 90.87 | 60.49 | 107.83 | 58.68 | 32.10 |
| KCND3 | 21.34 | 9.33 | 3.20 | 4.75 | 7.05 |
| MLLT3 | 28.09 | 15.52 | 14.98 | 15.44 | 11.00 |
| PTEN | 529.74 | 255.65 | 593.15 | 336.27 | 216.26 |
| SCG2 | 12.21 | 9.89 | 6.22 | 14.02 | 4.14 |
| SNTB1 | 40.11 | 22.02 | 31.45 | 35.57 | 19.80 |
| ISG20 | 13.25 | 7.51 | 6.90 | 8.11 | 5.33 |
| TMEM134 | 95.84 | 64.21 | 65.22 | 60.70 | 45.74 |
| DCP2 | 25.57 | 16.55 | 40.22 | 16.04 | 11.00 |
| DDIT3 | 73.95 | 40.82 | 59.48 | 51.28 | 31.35 |
| GOLGA8A | 58.84 | 27.03 | 40.00 | 64.50 | 21.16 |
| NUPR1 | 526.92 | 233.92 | 483.31 | 287.28 | 205.00 |
| BNIP3 | 1172.68 | 878.97 | 1025.27 | 838.88 | 263.72 |
| GCNT4 | 97.55 | 62.94 | 118.48 | 31.45 | 42.69 |
| FIBIN | 14.73 | 7.43 | 7.58 | 22.78 | 7.12 |
| THAP5 | 158.85 | 91.97 | 180.66 | 92.49 | 78.40 |
| NAALADL2 | 41.97 | 17.66 | 28.43 | 22.25 | 16.29 |
| C5orf46 | 43.90 | 18.93 | 17.85 | 36.17 | 8.09 |
| BBS12 | 12.73 | 7.75 | 6.67 | 9.09 | 6.15 |
| TMEM45A | 221.48 | 165.01 | 212.24 | 148.15 | 91.30 |
| CEP97 | 15.33 | 8.62 | 10.90 | 6.77 | 6.45 |
| PLCB1 | 23.27 | 11.16 | 16.57 | 17.24 | 6.97 |
| PAPPA | 305.71 | 211.96 | 262.40 | 151.89 | 143.04 |
| SLC8A1 | 83.67 | 57.79 | 50.26 | 57.41 | 41.49 |
| NOG | 50.58 | 34.00 | 29.11 | 19.63 | 17.34 |
| KCTD16 | 38.33 | 23.05 | 27.22 | 24.80 | 11.15 |
| EMILIN3 | 33.44 | 15.76 | 35.15 | 14.55 | 10.78 |
| OLFML1 | 15.40 | 7.35 | 10.68 | 17.02 | 4.29 |
| KMT5A | 60.89 | 27.26 | 82.75 | 25.69 | 28.07 |
| ZFP1 | 10.73 | 5.45 | 12.64 | 5.87 | 5.33 |
| NDUFA4L2 | 1202.07 | 573.98 | 588.62 | 1133.47 | 32.92 |
| ZNF17 | 9.84 | 6.39 | 11.28 | 5.87 | 3.92 |
| LINC00643 | 7.76 | 7.83 | 15.06 | 12.98 | 3.39 |
| EIF4EBP1 | 276.99 | 122.18 | 249.33 | 193.25 | 104.20 |
| HYKK | 12.43 | 6.72 | 11.89 | 3.48 | 5.63 |
| OSTN | 293.39 | 238.13 | 344.30 | 190.49 | 132.68 |
| RELN | 11.32 | 1.72 | 21.40 | 1.98 | 2.87 |
| ARID2 | 31.73 | 16.71 | 40.59 | 25.62 | 12.19 |
| SRGAP2B | 159.87 | 87.29 | 162.99 | 79.09 | 69.88 |
| FUT11 | 34.10 | 22.34 | 25.94 | 29.51 | 13.01 |
| ZNF398 | 12.43 | 10.44 | 9.54 | 9.09 | 5.56 |
| PDGFA | 115.51 | 51.21 | 130.19 | 97.13 | 49.92 |
| ZNF615 | 14.44 | 7.59 | 9.09 | 10.36 | 7.20 |
| ERO1A | 271.87 | 160.65 | 242.76 | 157.20 | 57.90 |
| ZNF248 | 13.70 | 12.56 | 11.51 | 12.15 | 5.93 |
| ZNF28 | 98.90 | 47.04 | 88.16 | 49.26 | 32.99 |
| GRK5 | 24.01 | 9.65 | 10.00 | 14.02 | 8.61 |
| MFAP3L | 24.23 | 13.78 | 2.14 | 23.45 | 11.00 |
| ZNF468 | 78.46 | 41.02 | 93.43 | 31.23 | 35.08 |
| ZSWIM7 | 16.44 | 12.27 | 17.70 | 11.48 | 8.02 |
| MIF-AS1 | 160.11 | 117.35 | 139.71 | 119.50 | 52.90 |
| EXOSC6 | 63.42 | 28.21 | 66.58 | 39.68 | 30.31 |
| LINC01133 | 34.55 | 13.70 | 19.89 | 23.15 | 15.85 |
| DUXAP9 | 21.61 | 12.03 | 14.53 | 13.51 | 9.69 |
| FAM212B-AS1 | 14.44 | 9.41 | 6.67 | 6.39 | 5.26 |
| CTB-89H12.4 | 25.05 | 15.53 | 22.90 | 21.27 | 11.16 |
| PET117 | 14.10 | 7.74 | 9.58 | 7.43 | 5.91 |
| CCDC144NL-AS1 | 20.08 | 9.18 | 6.90 | 11.33 | 6.90 |
| ZNF37BP | 21.64 | 14.65 | 18.68 | 10.81 | 9.28 |
| RP4-756G23.5 | 12.14 | 4.89 | 5.69 | 13.13 | 5.48 |
| MSC-AS1 | 35.66 | 15.36 | 15.06 | 39.68 | 9.81 |
| RP11-230B22.1 | 13.10 | 12.59 | 18.16 | 13.72 | 5.56 |
| LINC00607 | 14.29 | 8.78 | 10.07 | 6.62 | 6.53 |
| MIF | 840.30 | 576.91 | 714.26 | 603.85 | 259.28 |
| INMT | 64.97 | 38.76 | 16.49 | 23.07 | 25.69 |
| RP4-735C1.4 | 11.77 | 5.92 | 14.15 | 4.67 | 5.26 |
| LINC00968 | 60.30 | 45.89 | 28.43 | 39.31 | 18.83 |
| STARD4-AS1 | 17.70 | 8.38 | 6.60 | 20.91 | 6.97 |
| MIR210HG | 12.36 | 7.03 | 6.75 | 11.85 | 4.74 |
| UBA6-AS1 | 15.92 | 10.21 | 6.82 | 13.42 | 7.49 |
| BCKDHA | 46.48 | 27.66 | 33.46 | 23.88 | 22.96 |
| ZNF286B | 21.60 | 11.34 | 25.69 | 8.07 | 7.93 |
| RP11-61L23.2 | 11.09 | 8.32 | 5.69 | 7.85 | 4.77 |
| LYN | 17.55 | 8.70 | 9.24 | 10.28 | 5.93 |
| PAPPA-AS1 | 137.18 | 76.19 | 104.89 | 44.09 | 46.12 |
| RP11-841O20.2 | 9.69 | 5.69 | 13.47 | 5.94 | 3.54 |
| GS1-358P8.4 | 16.89 | 8.54 | 14.98 | 10.51 | 3.69 |
| LINC01355 | 11.99 | 6.88 | 8.86 | 6.47 | 3.54 |
| RP13-638C3.4 | 13.10 | 5.61 | 7.50 | 6.32 | 5.56 |
| TXNIP | 135.77 | 123.93 | 87.74 | 176.72 | 66.17 |
| ITGB1P1 | 105.51 | 64.55 | 106.27 | 76.44 | 47.94 |
| HIST2H4B | 33.86 | 18.24 | 19.13 | 7.86 | 7.22 |
| CTB-43P18.1 | 41.89 | 23.45 | 39.84 | 25.24 | 19.80 |
| RP11-67L2.2 | 11.25 | 6.16 | 7.50 | 7.07 | 4.29 |
| RP11-180C16.1 | 10.95 | 6.64 | 12.72 | 7.29 | 4.96 |
| ZNF280B | 19.11 | 20.52 | 16.49 | 14.02 | 7.49 |
| ZNF229 | 24.68 | 14.49 | 10.22 | 13.57 | 9.81 |
| TGFB2-OT1 | 15.71 | 6.56 | 10.17 | 4.11 | 7.39 |
| NDUFAF7 | 17.85 | 8.86 | 12.57 | 14.92 | 11.89 |
| DHX33 | 13.55 | 5.85 | 15.29 | 8.19 | 8.17 |
| RWDD2A | 26.68 | 11.16 | 11.58 | 14.02 | 13.68 |
| RGPD5 | 23.99 | 2.33 | 10.96 | 15.50 | 12.87 |
| AKAP11 | 24.23 | 10.21 | 21.71 | 14.32 | 14.65 |
| WISP2 | 39.67 | 17.03 | 7.96 | 62.27 | 19.95 |
| INPP5A | 20.23 | 7.67 | 16.80 | 13.95 | 15.25 |
| ADGRF5 | 34.55 | 13.38 | 6.52 | 15.00 | 29.49 |
| GPATCH1 | 16.07 | 6.16 | 14.91 | 10.96 | 11.22 |
| NCOA1 | 22.38 | 8.22 | 18.08 | 9.54 | 12.12 |
| JOSD1 | 25.27 | 8.14 | 16.72 | 22.03 | 13.31 |
| MEDAG | 32.03 | 13.70 | 32.36 | 16.94 | 25.39 |
| PPP2CB | 44.79 | 17.82 | 35.61 | 26.14 | 25.24 |
| SLC38A1 | 32.92 | 11.87 | 19.29 | 16.04 | 20.32 |
| C12orf49 | 46.87 | 20.91 | 53.21 | 26.22 | 30.83 |
| TTC33 | 21.04 | 9.97 | 27.07 | 17.46 | 10.85 |
| NPHP3 | 23.49 | 11.46 | 15.13 | 20.23 | 12.19 |
| WNT5A | 148.90 | 60.49 | 113.65 | 91.89 | 93.61 |
| TTL | 44.64 | 19.56 | 35.61 | 27.04 | 33.81 |
| F3 | 31.65 | 6.32 | 15.29 | 5.79 | 51.34 |
| PPP1R8 | 19.48 | 7.19 | 18.00 | 16.42 | 10.40 |
| IFIT2 | 14.63 | 6.72 | 22.13 | 11.11 | 29.09 |
| LRP11 | 18.37 | 8.94 | 11.13 | 17.46 | 14.73 |
| ZNF211 | 18.82 | 8.70 | 18.08 | 8.79 | 13.68 |
| RWDD3 | 20.58 | 9.15 | 19.09 | 13.69 | 11.27 |
| DCAF10 | 37.44 | 16.79 | 26.54 | 23.52 | 29.56 |
| XG | 25.87 | 11.71 | 16.95 | 7.14 | 16.89 |
| MTERF1 | 17.11 | 8.54 | 15.44 | 9.39 | 9.28 |
| PPFIA1 | 44.34 | 20.28 | 35.83 | 19.48 | 27.18 |
| KRT34 | 47.53 | 17.82 | 54.49 | 23.97 | 27.40 |
| UNK | 18.07 | 5.92 | 16.19 | 14.85 | 13.09 |
| POSTN | 1094.46 | 352.24 | 323.75 | 1058.96 | 665.92 |
| RRAS2 | 50.65 | 24.64 | 35.43 | 41.85 | 30.23 |
| ROCK2 | 31.80 | 15.68 | 34.02 | 31.60 | 24.34 |
| ADAM19 | 400.02 | 193.24 | 422.49 | 133.56 | 248.25 |
| NAB1 | 11.91 | 5.45 | 9.24 | 10.21 | 8.61 |
| ZSCAN32 | 18.00 | 7.75 | 14.68 | 10.73 | 12.34 |
| LYST | 17.04 | 6.88 | 20.88 | 13.87 | 9.58 |
| VPS54 | 11.40 | 5.61 | 15.06 | 6.77 | 8.84 |
| RNF145 | 14.07 | 6.24 | 13.25 | 8.71 | 7.72 |
| TLK2 | 26.30 | 11.86 | 24.72 | 14.01 | 13.76 |
| TIMM8B | 80.78 | 35.90 | 71.49 | 45.82 | 42.46 |
| ASAP2 | 34.99 | 13.93 | 36.44 | 22.93 | 26.06 |
| TBCEL | 17.41 | 7.91 | 14.98 | 10.43 | 9.36 |
| CCSAP | 17.48 | 8.46 | 6.22 | 14.77 | 9.21 |
| USP25 | 13.18 | 6.08 | 15.74 | 11.03 | 6.67 |
| DBI | 131.09 | 63.58 | 142.66 | 89.95 | 70.12 |
| PXYLP1 | 20.30 | 9.33 | 8.33 | 9.46 | 10.55 |
| MMP16 | 15.11 | 6.96 | 14.98 | 24.57 | 10.33 |
| CCDC28B | 15.48 | 7.11 | 7.88 | 11.78 | 9.51 |
| GLB1L | 13.99 | 6.96 | 8.94 | 6.09 | 7.49 |
| PGRMC2 | 97.40 | 48.51 | 53.89 | 43.35 | 52.16 |
| C9orf64 | 10.95 | 4.42 | 8.33 | 15.37 | 8.61 |
| PIGA | 13.55 | 5.61 | 17.17 | 9.24 | 9.81 |
| SPTSSA | 69.72 | 23.93 | 53.97 | 53.45 | 38.96 |
| B2M | 3390.99 | 1643.39 | 3155.22 | 2088.27 | 1998.52 |
| MPLKIP | 45.83 | 19.41 | 31.60 | 26.67 | 24.79 |
| KLHL30 | 20.45 | 10.05 | 9.47 | 12.90 | 17.34 |
| HNRNPH1 | 399.65 | 185.37 | 570.22 | 213.70 | 213.13 |
| ZNF318 | 15.03 | 5.69 | 6.97 | 10.43 | 9.28 |
| MALT1 | 16.37 | 6.08 | 13.02 | 10.13 | 9.66 |
| PTPRM | 97.85 | 48.43 | 65.60 | 78.80 | 50.22 |
| DLEU1 | 11.76 | 5.83 | 6.54 | 8.11 | 7.60 |
| METRNL | 13.25 | 5.85 | 11.51 | 9.09 | 8.84 |
| RCC2 | 15.77 | 6.72 | 12.19 | 14.62 | 10.40 |
| HCLS1 | 13.47 | 2.43 | 15.51 | 1.38 | 8.02 |
| TMEM136 | 18.37 | 7.99 | 7.50 | 11.33 | 10.78 |
| FAM101B | 51.17 | 22.74 | 32.96 | 23.07 | 32.32 |
| NKRF | 11.10 | 4.42 | 8.11 | 5.50 | 7.27 |
| PPARA | 10.06 | 4.89 | 13.47 | 10.51 | 9.51 |
| ADA | 120.11 | 52.87 | 41.42 | 114.41 | 82.58 |
| CDC42SE1 | 202.26 | 99.26 | 273.43 | 104.24 | 101.51 |
| DDX42 | 47.53 | 19.96 | 56.69 | 22.25 | 25.76 |
| ZNF26 | 18.89 | 6.96 | 11.36 | 12.90 | 11.22 |
| FICD | 105.19 | 52.55 | 145.00 | 49.93 | 63.34 |
| CCDC167 | 44.71 | 22.18 | 48.45 | 28.91 | 25.31 |
| SERF1B | 40.22 | 19.66 | 33.61 | 28.18 | 24.18 |
| TCTEX1D2 | 15.87 | 7.83 | 15.21 | 12.83 | 9.73 |
| CHUK | 21.12 | 10.52 | 11.28 | 15.89 | 12.64 |
| GOLGA8B | 20.87 | 8.69 | 19.59 | 20.77 | 11.44 |
| APOL6 | 11.10 | 5.45 | 8.41 | 21.73 | 10.55 |
| GAS5 | 45.31 | 18.69 | 39.01 | 27.86 | 23.08 |
| PSMB9 | 7.91 | 2.99 | 10.45 | 5.65 | 12.79 |
| TPM3P9 | 11.99 | 5.37 | 12.72 | 8.41 | 6.75 |
| ZNF702P | 25.12 | 11.87 | 18.89 | 12.38 | 13.46 |
| HLA-L | 11.91 | 5.53 | 8.33 | 8.64 | 6.45 |
| PSMA3-AS1 | 65.34 | 30.27 | 69.15 | 43.80 | 33.51 |
| LA16c-306E5.2 | 15.70 | 5.92 | 12.49 | 10.06 | 12.19 |
| CTC-459F4.3 | 11.84 | 5.69 | 5.77 | 6.17 | 5.93 |
| CTC-429P9.3 | 13.32 | 5.05 | 17.10 | 8.86 | 9.28 |
| SNHG8 | 29.80 | 14.49 | 22.92 | 20.23 | 18.75 |
| TSIX | 107.64 | 51.29 | 98.39 | 107.75 | 67.51 |
| HIST2H4A | 31.30 | 12.64 | 43.62 | 21.09 | 27.76 |
| SRXN1 | 30.17 | 14.81 | 18.61 | 24.42 | 17.19 |
| SOCS7 | 11.32 | 5.29 | 7.65 | 6.39 | 6.97 |
| RP5-875H18.9 | 14.07 | 6.48 | 16.57 | 6.17 | 8.09 |
| WNT16 | 9.02 | 5.05 | 11.36 | 3.92 | 12.71 |
| CCL26 | 16.66 | 11.87 | 17.40 | 6.84 | 12.04 |
| AASS | 10.06 | 9.73 | 12.64 | 4.75 | 6.53 |
| DNASE1L1 | 12.66 | 10.92 | 7.96 | 3.78 | 9.51 |
| GPRC5A | 65.34 | 86.58 | 103.22 | 16.87 | 77.51 |
| BID | 94.95 | 55.65 | 106.93 | 41.85 | 62.07 |
| BRD9 | 17.41 | 11.16 | 21.18 | 8.11 | 11.15 |
| HMGB3 | 21.04 | 16.95 | 27.75 | 10.13 | 18.98 |
| FOXC1 | 13.03 | 8.86 | 8.56 | 5.20 | 10.03 |
| FAR2 | 28.76 | 22.97 | 27.60 | 14.25 | 27.92 |
| CSNK2A2 | 114.25 | 81.74 | 135.56 | 51.20 | 70.20 |
| CDC42 | 528.92 | 312.27 | 577.29 | 244.64 | 352.70 |
| SEMA3A | 12.88 | 7.59 | 9.92 | 1.91 | 10.48 |
| LRRC16A | 44.94 | 23.05 | 56.99 | 18.81 | 32.17 |
| FAM234B | 32.40 | 22.82 | 37.35 | 14.17 | 28.59 |
| B4GALT1 | 1419.87 | 917.68 | 1576.49 | 635.27 | 884.31 |
| ITGA6 | 20.89 | 14.96 | 16.04 | 9.98 | 14.20 |
| ZNF516 | 16.00 | 11.87 | 15.21 | 4.07 | 12.34 |
| DNAJC17 | 15.03 | 9.26 | 15.21 | 7.29 | 10.10 |
| MEST | 255.84 | 287.29 | 141.75 | 100.50 | 379.92 |
| TMEM245 | 192.02 | 127.66 | 153.77 | 86.73 | 118.36 |
| TGFBR1 | 163.67 | 107.83 | 96.12 | 78.88 | 96.97 |
| INTS2 | 15.55 | 13.85 | 22.76 | 7.74 | 11.00 |
| CCL2 | 164.04 | 132.33 | 282.73 | 65.41 | 206.64 |
| BST1 | 135.99 | 69.13 | 85.02 | 60.18 | 90.70 |
| FOLR3 | 20.08 | 10.05 | 22.99 | 8.71 | 15.47 |
| ARHGDIB | 35.29 | 25.35 | 48.07 | 15.67 | 23.23 |
| NT5DC3 | 170.42 | 112.91 | 246.84 | 56.06 | 120.75 |
| SMIM8 | 13.40 | 7.19 | 14.23 | 6.54 | 8.39 |
| HINT3 | 53.17 | 31.14 | 41.42 | 23.30 | 33.96 |
| IGFBP2 | 27.05 | 18.22 | 16.64 | 11.48 | 25.31 |
| PLEKHA3 | 13.77 | 9.18 | 13.77 | 6.39 | 7.05 |
| EPHA4 | 13.99 | 7.83 | 8.64 | 6.84 | 11.60 |
| SPP1 | 78.78 | 55.96 | 99.45 | 34.37 | 41.12 |
| SET | 1003.78 | 512.68 | 1414.74 | 469.66 | 518.97 |
| BICC1 | 300.21 | 312.59 | 485.20 | 132.21 | 262.34 |
| TIMM8A | 20.89 | 12.11 | 19.36 | 9.91 | 16.29 |
| ZNF557 | 37.22 | 23.29 | 37.27 | 18.14 | 19.13 |
| PTCD3 | 91.02 | 71.98 | 93.10 | 45.07 | 67.81 |
| NHSL1 | 11.40 | 8.22 | 18.61 | 4.15 | 12.19 |
| TMEM8B | 15.85 | 9.81 | 8.03 | 5.94 | 9.21 |
| RNF144B | 36.26 | 27.26 | 48.60 | 16.79 | 27.77 |
| GGH | 85.97 | 55.57 | 62.20 | 42.15 | 75.19 |
| PLCE1 | 19.86 | 12.03 | 17.40 | 7.89 | 16.29 |
| PDE5A | 48.72 | 38.52 | 35.46 | 23.07 | 44.25 |
| CPNE8 | 10.50 | 5.45 | 15.96 | 2.58 | 10.70 |
| MPC2 | 301.40 | 241.06 | 298.14 | 139.17 | 182.04 |
| C1orf56 | 180.37 | 91.18 | 259.61 | 88.68 | 94.88 |
| CALM2 | 3857.68 | 1996.37 | 4063.24 | 1894.08 | 2082.32 |
| FAM171B | 353.35 | 308.23 | 479.30 | 144.11 | 268.00 |
| EAF1 | 78.92 | 48.67 | 90.38 | 33.32 | 51.19 |
| OCIAD2 | 74.25 | 52.24 | 113.72 | 35.94 | 60.51 |
| 6-Mar | 381.47 | 265.09 | 489.12 | 173.58 | 231.62 |
| NFIB | 28.02 | 23.05 | 27.90 | 11.41 | 31.06 |
| PIP4K2A | 122.93 | 72.22 | 181.79 | 56.74 | 86.75 |
| NOCT | 13.62 | 9.18 | 13.77 | 6.24 | 11.07 |
| SUV39H2 | 9.17 | 6.72 | 15.36 | 4.45 | 4.74 |
| PLEKHH2 | 14.22 | 9.73 | 12.41 | 6.17 | 12.42 |
| RASSF3 | 45.46 | 30.67 | 56.38 | 21.28 | 28.59 |
| DEPTOR | 45.01 | 29.95 | 29.03 | 10.73 | 41.79 |
| KIT | 13.32 | 15.36 | 16.12 | 2.65 | 8.39 |
| AAED1 | 65.34 | 45.66 | 69.98 | 32.35 | 43.06 |
| EYA3 | 114.77 | 70.95 | 167.97 | 52.02 | 90.18 |
| HIST1H4H | 49.24 | 35.35 | 38.03 | 12.45 | 36.50 |
| TMED4 | 376.87 | 214.73 | 462.46 | 170.36 | 246.53 |
| CAMK2N1 | 18.52 | 10.60 | 14.76 | 7.67 | 16.81 |
| SERPINI1 | 27.94 | 19.09 | 22.08 | 11.55 | 14.95 |
| EIF5A2 | 37.52 | 25.59 | 34.55 | 17.54 | 30.68 |
| TMEM161B | 14.81 | 7.91 | 16.12 | 6.92 | 9.13 |
| SERINC5 | 261.63 | 141.45 | 349.28 | 105.51 | 160.12 |
| REEP3 | 543.25 | 350.34 | 634.55 | 247.41 | 299.77 |
| RPL36AL | 196.40 | 102.75 | 284.31 | 97.50 | 126.27 |
| PDZD8 | 101.11 | 67.46 | 135.63 | 46.94 | 60.66 |
| TK2 | 170.94 | 108.70 | 235.36 | 85.46 | 104.64 |
| NKX3-1 | 31.73 | 29.32 | 52.30 | 14.10 | 21.96 |
| POP5 | 50.80 | 26.23 | 42.10 | 22.93 | 26.95 |
| PAFAH1B2 | 526.55 | 397.61 | 838.46 | 234.10 | 363.66 |
| TTC39C | 56.74 | 48.43 | 81.84 | 26.96 | 52.98 |
| CMAHP | 27.94 | 34.08 | 17.78 | 13.20 | 30.68 |
| GPR183 | 12.43 | 6.96 | 14.98 | 6.02 | 13.46 |
| SIK2 | 16.59 | 18.93 | 8.64 | 5.65 | 16.14 |
| HAS2 | 567.29 | 372.55 | 1101.22 | 279.35 | 327.73 |
| C1GALT1C1 | 60.52 | 41.77 | 72.55 | 29.51 | 48.65 |
| OLR1 | 11.84 | 6.24 | 7.58 | 2.35 | 10.85 |
| TRAF6 | 69.13 | 44.23 | 78.22 | 25.84 | 40.52 |
| ZNF613 | 16.44 | 9.65 | 21.03 | 6.47 | 8.69 |
| VSIG10 | 28.54 | 22.66 | 45.05 | 9.46 | 22.48 |
| NFATC2IP | 10.88 | 6.24 | 7.28 | 4.82 | 12.94 |
| ZBTB41 | 28.61 | 14.57 | 32.13 | 14.25 | 15.32 |
| HIST1H2BC | 23.27 | 17.11 | 17.10 | 8.26 | 18.98 |
| EFNA5 | 19.19 | 18.22 | 34.40 | 6.99 | 13.24 |
| CHP1 | 625.39 | 459.30 | 1020.83 | 307.55 | 454.70 |
| ZNF286A | 79.43 | 45.71 | 105.73 | 34.19 | 42.40 |
| BLOC1S3 | 39.67 | 24.40 | 35.53 | 16.49 | 29.64 |
| TSPYL1 | 755.18 | 524.89 | 1201.70 | 332.99 | 492.43 |
| STRN3 | 334.57 | 168.73 | 406.47 | 157.42 | 175.03 |
| ATL1 | 15.77 | 16.87 | 8.94 | 5.42 | 31.35 |
| HLA-DMA | 14.44 | 10.60 | 13.47 | 6.92 | 16.44 |
| PSG5 | 192.25 | 175.20 | 255.11 | 91.89 | 229.33 |
| LIN52 | 36.11 | 20.52 | 30.47 | 15.22 | 19.27 |
| PPP1CB | 792.44 | 450.10 | 1154.18 | 389.31 | 401.01 |
| NUDT19 | 298.36 | 158.66 | 381.85 | 108.65 | 161.24 |
| SFTA1P | 147.64 | 75.16 | 161.32 | 61.45 | 77.21 |
| JPX | 41.83 | 21.88 | 35.86 | 20.55 | 21.14 |
| LINC01013 | 21.34 | 13.06 | 6.82 | 9.54 | 11.15 |
| PSG1 | 22.67 | 23.41 | 23.83 | 7.29 | 16.91 |
| PSG2 | 17.55 | 9.41 | 14.03 | 5.42 | 26.81 |
| RP11-538P18.2 | 14.88 | 8.86 | 26.16 | 6.84 | 7.57 |
| LINC01085 | 14.07 | 10.05 | 26.77 | 5.79 | 11.07 |
| RP11-363E7.4 | 19.63 | 12.82 | 17.78 | 7.52 | 10.70 |
| RP11-294J22.6 | 77.81 | 43.43 | 104.36 | 33.85 | 40.30 |
| RP11-101E13.5 | 29.72 | 23.21 | 49.21 | 14.77 | 18.23 |
| LINC00547 | 15.11 | 9.97 | 8.94 | 4.00 | 10.03 |
| CLCN6 | 10.06 | 12.82 | 4.56 | 9.68 | 14.65 |
| APBA2 | 7.76 | 10.44 | 3.65 | 13.65 | 8.39 |
| PNKP | 7.02 | 12.59 | 3.50 | 8.86 | 12.12 |
| INPP4A | 18.89 | 10.29 | 8.26 | 12.83 | 12.49 |
| CNTLN | 14.66 | 8.46 | 6.97 | 11.11 | 10.03 |
| COL11A1 | 19.19 | 19.88 | 7.65 | 21.65 | 30.53 |
| ZNF275 | 35.07 | 24.96 | 14.30 | 25.32 | 30.38 |
| NEO1 | 11.40 | 8.46 | 3.95 | 13.50 | 12.04 |
| TBC1D25 | 10.43 | 13.62 | 4.41 | 13.28 | 17.71 |
| SMG6 | 14.66 | 25.75 | 6.29 | 26.96 | 19.65 |
| FSTL3 | 35.07 | 60.01 | 6.22 | 69.83 | 58.27 |
| LMCD1 | 69.80 | 71.75 | 21.18 | 50.98 | 72.66 |
| UNG | 20.15 | 19.80 | 9.85 | 18.51 | 18.23 |
| NOA1 | 15.92 | 15.92 | 7.28 | 13.95 | 20.99 |
| COL16A1 | 40.19 | 37.41 | 10.07 | 66.16 | 52.60 |
| CAD | 13.40 | 16.71 | 5.84 | 16.12 | 21.14 |
| HEPH | 12.21 | 17.34 | 5.09 | 7.07 | 19.72 |
| XXbac-B461K10.4 | 17.93 | 12.37 | 6.07 | 13.00 | 13.80 |
| EFHC1 | 15.48 | 12.11 | 6.82 | 19.71 | 15.03 |
| MZF1 | 8.65 | 12.51 | 2.67 | 7.81 | 9.06 |
| TRMT2A | 16.81 | 27.81 | 3.88 | 21.58 | 20.47 |
| TBC1D10A | 35.29 | 47.08 | 15.11 | 49.60 | 43.83 |
| PIK3IP1 | 42.27 | 47.48 | 20.20 | 33.32 | 23.52 |
| ABHD4 | 195.65 | 230.99 | 74.67 | 261.25 | 164.74 |
| PCK2 | 32.10 | 19.64 | 15.81 | 57.63 | 18.38 |
| NFATC4 | 42.56 | 56.76 | 21.10 | 56.21 | 53.57 |
| TRIB3 | 67.94 | 44.86 | 25.48 | 123.99 | 43.51 |
| NOMO3 | 17.96 | 19.75 | 8.16 | 11.57 | 23.34 |
| BRF2 | 31.06 | 29.56 | 15.44 | 23.45 | 22.41 |
| TRMT1 | 16.96 | 16.23 | 7.65 | 17.24 | 21.66 |
| DLX5 | 12.21 | 16.08 | 4.71 | 10.96 | 21.59 |
| UNC5B | 8.50 | 7.83 | 2.59 | 9.91 | 16.44 |
| TBC1D12 | 12.66 | 7.91 | 4.25 | 9.16 | 10.78 |
| COL12A1 | 171.98 | 242.96 | 81.77 | 165.58 | 251.97 |
| SPARC | 11294.76 | 8427.19 | 4445.06 | 15545.76 | 7924.75 |
| IGFBP5 | 65.86 | 82.06 | 21.18 | 96.98 | 59.61 |
| PARD3B | 11.25 | 10.52 | 4.03 | 13.35 | 10.78 |
| CD3EAP | 17.33 | 12.98 | 8.26 | 19.04 | 15.62 |
| PLXDC2 | 12.14 | 10.05 | 5.16 | 6.69 | 9.81 |
| ECHDC2 | 46.12 | 23.69 | 22.16 | 31.60 | 27.10 |
| GLIPR2 | 61.41 | 66.43 | 29.94 | 75.29 | 53.87 |
| TNFAIP6 | 13.92 | 7.35 | 6.29 | 18.96 | 11.67 |
| KDM5C | 27.72 | 40.42 | 13.77 | 55.39 | 54.47 |
| SLC10A3 | 30.10 | 26.31 | 14.00 | 31.83 | 43.36 |
| TUBGCP6 | 7.09 | 7.03 | 3.12 | 9.16 | 13.16 |
| POT1 | 13.32 | 8.86 | 5.77 | 7.89 | 10.33 |
| CNN1 | 147.34 | 176.51 | 72.55 | 204.55 | 145.88 |
| COL5A1 | 324.70 | 261.84 | 157.62 | 527.55 | 270.09 |
| TRAF3 | 13.62 | 9.02 | 4.56 | 8.56 | 12.56 |
| FBXW9 | 13.40 | 9.57 | 5.92 | 12.60 | 10.40 |
| MYH11 | 8.35 | 8.14 | 3.95 | 15.74 | 15.85 |
| LARGE1 | 12.14 | 7.83 | 2.22 | 8.86 | 8.39 |
| ITGA7 | 168.05 | 212.59 | 83.51 | 229.61 | 316.02 |
| SEMA4F | 15.25 | 18.69 | 7.50 | 13.28 | 18.38 |
| DYSF | 16.81 | 11.79 | 7.43 | 17.02 | 9.36 |
| BIN1 | 15.63 | 19.41 | 5.92 | 28.91 | 21.81 |
| DNAJB5 | 16.66 | 10.13 | 7.50 | 10.21 | 12.71 |
| SLC22A23 | 21.27 | 15.92 | 7.50 | 16.49 | 15.25 |
| IFT172 | 7.39 | 8.07 | 3.12 | 14.70 | 6.30 |
| LRIG3 | 16.00 | 11.40 | 4.25 | 22.18 | 10.18 |
| LUM | 880.30 | 568.35 | 427.17 | 611.18 | 789.32 |
| SLC46A3 | 18.15 | 12.82 | 9.01 | 18.59 | 11.60 |
| FBLN5 | 149.87 | 160.01 | 70.13 | 142.84 | 154.75 |
| ULK3 | 10.06 | 14.65 | 2.82 | 15.30 | 18.08 |
| HAPLN3 | 71.50 | 72.06 | 21.03 | 122.64 | 81.68 |
| SGSM2 | 12.51 | 12.19 | 6.07 | 10.66 | 14.80 |
| SMYD2 | 98.37 | 61.44 | 42.63 | 79.63 | 61.48 |
| AFF3 | 16.96 | 14.33 | 4.56 | 17.39 | 9.28 |
| SPAG16 | 22.68 | 14.33 | 10.45 | 21.80 | 15.99 |
| LRIG1 | 9.91 | 9.02 | 3.95 | 15.00 | 10.03 |
| CDKN2B | 90.13 | 72.78 | 33.27 | 82.02 | 54.91 |
| ADAM12 | 126.42 | 104.90 | 59.78 | 112.76 | 108.67 |
| AKAP6 | 6.05 | 11.48 | 2.67 | 9.01 | 11.97 |
| NUBPL | 11.62 | 11.24 | 5.69 | 10.06 | 10.18 |
| AMN1 | 18.59 | 15.76 | 9.17 | 13.20 | 11.82 |
| ZNF776 | 15.40 | 11.63 | 7.05 | 10.96 | 9.13 |
| AFAP1L1 | 19.19 | 11.87 | 6.90 | 18.51 | 10.33 |
| RUNX1 | 22.23 | 18.38 | 10.07 | 24.50 | 17.78 |
| NR2F6 | 13.40 | 11.40 | 6.67 | 12.45 | 11.30 |
| PLXDC1 | 14.07 | 10.21 | 5.54 | 23.37 | 8.84 |
| FDXR | 9.09 | 9.73 | 3.27 | 11.11 | 8.54 |
| ALPL | 70.09 | 109.02 | 30.02 | 98.03 | 133.35 |
| MEGF6 | 18.59 | 25.67 | 5.69 | 26.59 | 21.96 |
| ZNF496 | 11.17 | 10.05 | 5.54 | 12.38 | 12.27 |
| PLXNB1 | 10.21 | 8.86 | 4.41 | 11.63 | 8.61 |
| FNDC1 | 9.17 | 14.81 | 4.33 | 10.51 | 11.74 |
| RPP25L | 15.77 | 11.71 | 7.73 | 10.51 | 16.37 |
| MAMDC2 | 8.43 | 7.27 | 4.10 | 11.63 | 4.59 |
| SVEP1 | 30.39 | 27.65 | 11.58 | 17.76 | 20.10 |
| ANKS6 | 13.84 | 7.99 | 5.01 | 8.19 | 9.06 |
| DACT1 | 17.48 | 9.10 | 8.03 | 20.98 | 10.03 |
| GPT2 | 22.68 | 17.34 | 8.71 | 13.95 | 17.63 |
| MFAP4 | 17.93 | 24.72 | 7.96 | 18.44 | 27.55 |
| ZNF91 | 17.93 | 9.26 | 4.48 | 14.92 | 8.99 |
| COL3A1 | 1382.39 | 1247.82 | 556.89 | 2454.36 | 1062.20 |
| TNIP2 | 7.91 | 6.48 | 3.95 | 4.97 | 13.38 |
| LRRC28 | 21.34 | 12.82 | 9.69 | 23.45 | 17.49 |
| ZNF212 | 10.95 | 12.51 | 3.35 | 15.37 | 10.03 |
| HINFP | 19.26 | 14.49 | 5.99 | 13.50 | 13.53 |
| ARNT2 | 8.20 | 10.44 | 3.95 | 5.35 | 12.34 |
| ZHX3 | 33.66 | 31.06 | 15.96 | 23.07 | 30.61 |
| PODN | 116.62 | 98.08 | 44.90 | 140.44 | 99.28 |
| IQCK | 11.77 | 10.68 | 5.01 | 10.51 | 13.53 |
| CSRP2 | 141.78 | 82.61 | 46.11 | 96.68 | 73.85 |
| ORAI3 | 11.38 | 7.68 | 4.88 | 8.83 | 8.27 |
| KCTD12 | 42.71 | 28.05 | 19.74 | 31.08 | 59.76 |
| MSC | 26.46 | 14.57 | 7.58 | 37.96 | 13.83 |
| FOXS1 | 46.35 | 36.06 | 17.78 | 88.90 | 57.30 |
| DHTKD1 | 9.39 | 11.79 | 4.48 | 14.47 | 14.28 |
| SETD4 | 16.29 | 8.86 | 7.43 | 8.19 | 9.06 |
| SPIN2B | 11.77 | 6.07 | 5.48 | 8.53 | 7.57 |
| ZSCAN30 | 15.63 | 13.38 | 5.39 | 16.79 | 12.27 |
| CYHR1 | 18.22 | 16.08 | 8.11 | 21.20 | 18.16 |
| S100A3 | 12.51 | 6.48 | 5.09 | 9.24 | 7.05 |
| ZNF569 | 17.85 | 12.74 | 8.41 | 13.50 | 9.51 |
| ZNF770 | 40.41 | 35.98 | 19.14 | 46.04 | 37.47 |
| FAM179B | 19.48 | 13.93 | 5.77 | 18.06 | 15.92 |
| EHMT2 | 15.03 | 22.66 | 6.29 | 16.27 | 19.35 |
| DDR1 | 15.85 | 24.64 | 7.35 | 24.65 | 23.23 |
| RBMXL1 | 12.13 | 7.31 | 3.66 | 11.69 | 9.01 |
| LBH | 75.59 | 84.12 | 35.61 | 100.57 | 82.05 |
| TRIM16 | 30.21 | 33.72 | 8.62 | 58.25 | 28.98 |
| AFG3L1P | 9.84 | 7.35 | 4.78 | 12.53 | 5.11 |
| ATXN1L | 12.66 | 9.89 | 5.16 | 9.54 | 12.56 |
| AQP1 | 13.77 | 16.15 | 5.09 | 16.79 | 9.06 |
| ZNF550 | 9.17 | 6.56 | 3.95 | 11.85 | 9.66 |
| RP1-140K8.5 | 11.40 | 6.16 | 3.80 | 8.19 | 5.85 |
| WFDC21P | 22.53 | 12.67 | 9.85 | 20.46 | 12.34 |
| HIST2H2AA4 | 20.65 | 30.03 | 7.76 | 18.17 | 32.25 |
