# Supplementary material for: Transcriptome and lipidome profile of human mesenchymal stem cells with reduced senescence and increased trilineage differentiation ability upon drug treatment
Source: Aging (Albany NY). 2021 Mar 26;13(7):9991–10014. doi: 10.18632/aging.202759 (PMC8064146; doi:10.18632/aging.202759)
Supplement: Supplementary Table 2 [file aging-13-202759-s003.docx]

Supplementary Table 2. Enriched GO terms for DEGs. GO enrichment analysis of the four drugs’ DEGs is performed on CPDB database [1, 2]. The enriched GO is retrieved by giving a cut-off 0.01 on the *p*-value. A relative loose cut-off allows us to find slightly influenced GOs by drugs.

| ***p*-value** | ***q*-value** | **term_goid** | **term_category** | **term_level** | **term_name** | **members_input_overlap** | **members_input_overlap_geneids** | **size** | **effective_size** |
| --- | --- | --- | --- | --- | --- | --- | --- | --- | --- |
| 1.02E-11 | 8.33E-09 | GO:0071310 | b | 4 | cellular response to organic substance | 27; 37; 60; 81; 331; 445; 65992; 467; 471; 476; 595; 598; 896; 972; 1265; 1291; 1388; 1435; 1490; 1535; 1545; 196883; 2002; 2022; 2064; 2134; 2252; 2621; 2817; 2852; 3162; 3164; 3297; 3340; 3371; 3397; 3429; 3434; 3437; 3455; 3481; 3572; 3575; 3590; 3636; 3675; 3726; 3856; 3875; 3953; 4000; 4055; 4313; 4502; 4641; 4681; 4926; 5055; 5058; 5118; 5127; 5167; 5176; 5179; 5371; 5598; 5599; 5606; 5696; 5905; 5916; 5978; 55627; 6300; 6424; 6464; 6624; 6774; 6778; 7041; 7128; 7200; 7421; 7538; 7791; 8200; 23213; 8425; 8519; 8611; 8751; 8877; 9076; 9146; 9230; 9531; 9612; 10014; 10025; 10113; 10312; 10370; 10376; 10432; 10435; 10437; 10458; 10581; 10678; 10938; 55738; 112398; 79876; 84335; 29988; 147372; 81858; 114990; 116372; 83667; 118813; 23558; 285704; 23586; 23641; 23770; 155066; 56928; 57175; 26119; 26585; 92140; 27429; 53834; 29965; 30827; 5170; 64855 | BCL2L1; HSF1; FGFRL1; ACADVL; IFI27; IL6ST; GPER1; SULF1; ASS1; ZYX; LTBR; SESN2; RARG; NDST1; AKT1S1; ERBB2; IFITM2; IFITM1; SHC1; EXTL1; RANGAP1; IFI30; ENPP1; CLDN1; SERPINB2; ADCY4; IFNAR2; ATIC; CNN2; CCND1; CCND3; SLC2A8; CSF1; ELK1; CXXC1; ACTB; SPPL2B; PAK1; IL7R; VDR; ITGA3; GREM1; ARFGAP1; PREB; IFIT3; IFIT1; DDX58; KRT8; NBL1; CTGF; RAB11B; LDOC1; FAM129B; PCOLCE; TGFB1I1; IGF2; TRH; ENG; UBA5; FGF7; STAT3; EGLN2; B3GNT2; HMOX1; TNFAIP3; TCIRG1; DDRGK1; IL11RA; BAIAP2; KRT18; CD74; BAG3; HDAC5; EHD1; MYO1C; FKBP8; SMPD4; XIAP; MAPK12; ABL2; RGMB; NCOR2; HTRA2; STAT6; CYP1B1; MAPK7; RBM14; PML; CITED2; MAPK8; PENK; PLPP1; CCBE1; LEPR; SERPINF1; FSCN1; CORO1B; ZFYVE27; SFRP4; ID1; ZFP36; ATP1A1; PSMB8; CDIP1; LDLRAP1; SPHK1; MT2A; NUMA1; JUNB; MAP2K3; ADAM15; MTDH; INPPL1; LYPD1; CDK16; GDF5; MED16; LTBP4; VASN; CDC42EP2; NR4A1; HGS; REST; GPC1; CYBA; TNC; PDPK1; LMNA; ATP6V0E2; ACTN4; SHARPIN; TUBA1B; COL6A1; WBP2; ATF6B; GAS6; MMP2; ATF3 | 2601 | 2596 |
| 1.04E-08 | 4.24E-06 | GO:0042127 | b | 4 | regulation of cell proliferation | 27; 331; 65992; 467; 595; 598; 823; 896; 972; 1435; 1490; 1535; 1545; 2022; 2064; 2192; 2252; 2621; 2852; 3084; 3159; 3162; 3164; 3297; 3371; 3397; 3398; 3437; 3481; 3489; 3572; 3575; 3590; 3636; 3776; 4000; 4209; 4313; 5058; 5176; 5270; 5328; 5371; 5880; 5916; 5978; 6424; 6464; 6774; 6778; 7041; 7052; 7128; 7421; 7538; 8200; 23213; 8519; 8558; 8611; 8877; 9055; 9133; 9146; 9500; 9536; 9537; 1717; 10312; 11070; 338773; 11180; 11285; 192669; 51157; 79647; 51548; 57124; 115908; 54962; 55214; 80381; 23641; 26119; 26585; 53834; 30851; 5170; 64855 | BCL2L1; HSF1; TGM2; FGFRL1; IL6ST; GPER1; KCNK2; FBLN1; LDOC1; RARG; P3H2; CAPN1; ERBB2; WDR6; IFITM1; SHC1; FAM129B; DHCR7; SFRP4; CCND1; MAGED1; CCND3; CSF1; PAK1; IL7R; VDR; TMEM119; IFIT3; TMEM115; SERPINE2; CTGF; SULF1; TGFB1I1; B4GALT7; IGF2; SIRT6; ENG; ZNF580; IGFBP6; REST; FGF7; STAT3; PTGES; TP53I11; CD276; HMOX1; TNFAIP3; CTHRC1; TCIRG1; MEF2D; IL11RA; AGO3; GAS6; CD74; DDRGK1; XIAP; ABL2; STAT6; CYP1B1; PML; PLPP1; HMGA1; SERPINF1; AKIRIN1; ID2; ID1; ZFP36; LDLRAP1; SPHK1; PRC1; CD248; INPPL1; TIPIN; CDK10; GDF5; NRG1; GREM1; RAC2; NR4A1; HGS; PLAU; CYBA; TNC; LMNA; CCNB2; TAX1BP3; PDPK1; MMP2; ATF3 | 1601 | 1592 |
| 8.80E-08 | 2.40E-05 | GO:0007167 | b | 4 | enzyme linked receptor protein signaling pathway | 60; 160; 331; 896; 1435; 1464; 1490; 1535; 2022; 2064; 2252; 2621; 2817; 2852; 3084; 3340; 3397; 3481; 3489; 3572; 3675; 4313; 4641; 4681; 4926; 5058; 5167; 5327; 5371; 5879; 6424; 6464; 6774; 7041; 7763; 7791; 7867; 8200; 23213; 8425; 9146; 9961; 10312; 10370; 10432; 10458; 84335; 29988; 147372; 114990; 93343; 116985; 118813; 285704; 23770; 155066; 26585; 92799; 53834; 5170; 64651; 64855 | IGF2; FGFRL1; NUMA1; ITGA3; MYO1C; FKBP8; IL6ST; GPER1; XIAP; GREM1; SULF1; RGMB; PML; MAPKAPK3; SHKBP1; ZYX; CCBE1; MVB12A; STAT3; ARAP1; NDST1; RAC1; CSRNP1; GDF5; AKT1S1; RBM14; NRG1; TGFB1I1; ERBB2; LTBP4; VASN; SHC1; ENG; FAM129B; HGS; ENPP1; MVP; GPC1; ZFAND5; CYBA; AP2A1; CITED2; FGF7; PLAT; ATP6V0E2; ZFYVE27; SFRP4; CSPG4; SLC2A8; CCND3; CSF1; ID1; TCIRG1; CTGF; PDPK1; NBL1; IGFBP6; ACTB; BAIAP2; MMP2; PAK1; GAS6 | 1009 | 1008 |
| 4.28E-07 | 8.75E-05 | GO:0009966 | b | 4 | regulation of signal transduction | 29; 81; 331; 396; 65992; 467; 598; 823; 896; 972; 1263; 1388; 1435; 1464; 1490; 1535; 1545; 1760; 1848; 2022; 2064; 2192; 2252; 2621; 2671; 2817; 2852; 2907; 3084; 3162; 3297; 3340; 3397; 3455; 3481; 3489; 3572; 3575; 3675; 3927; 4000; 4055; 4192; 4550; 4641; 4681; 4926; 5058; 5167; 5179; 5270; 5328; 5371; 5536; 5598; 5599; 5606; 5879; 5880; 5998; 6275; 6424; 6455; 6464; 6574; 6774; 6878; 7041; 7052; 7070; 7128; 7200; 7867; 6790; 8200; 23213; 8425; 8482; 8558; 8613; 8877; 9146; 9265; 9322; 9454; 9500; 9612; 9828; 10174; 10370; 10435; 10758; 192669; 400916; 79156; 55738; 84231; 79876; 79971; 84335; 375033; 29984; 147372; 81858; 114990; 115908; 93343; 83596; 116372; 83667; 51246; 116985; 84934; 128869; 54386; 55343; 22848; 23274; 23558; 23641; 23770; 56926; 56928; 57153; 89941; 253980; 25894; 25987; 26119; 91663; 26155; 124583; 26585; 92140; 92799; 27352; 27429; 30851; 5170; 64855; 64857 | BCL2L1; HSF1; TGM2; PLK3; WLS; IL6ST; GPER1; GDF5; FBLN1; FKBP8; LDOC1; LTBR; NDST1; DMPK; THY1; ERBB2; SHC1; ARHGEF17; GRINA; RGS3; ENPP1; TERF2IP; TRAF3IP2; SEMA7A; KCTD13; IFNAR2; CSPG4; MAGED1; CCND3; CSF1; SPPL2B; PAK1; IL7R; ITGA3; GREM1; ARFGAP1; VASN; MTDH; MAPKAPK3; SERPINE2; GFER; SHKBP1; NBL1; TRAF7; NCOR2; AKT1S1; FAM129B; TGFB1I1; IGF2; TRH; ENG; UBA5; IGFBP6; FGF7; STAT3; RHOD; TAF6; PPP5C; HMOX1; AAK1; TNFAIP3; LDLRAP1; SHISA5; DDRGK1; AGO3; GAS6; CD74; SESN2; MYO1C; MT-RNR2; XIAP; RHOT2; CHCHD10; SH3GL1; LMNA; HTRA2; MDK; CYP1B1; NOC2L; MAPK7; PML; S100A4; MAPK8; DUSP6; PENK; SLC35C1; PLPP3; TSKU; CAPN1; CCBE1; CITED2; LASP1; RITA1; MVB12A; SFRP4; ID1; CTGF; CTHRC1; SPHK1; NUMA1; CLEC16A; MYADM; MAP2K3; SORBS3; PLEKHF1; ARAP1; BCL2L12; CDK10; PEAR1; SULF1; AURKA; ARHGDIA; NRG1; HOMER3; SLC44A2; LTBP4; RAC2; RAC1; CDC42EP2; HGS; PLAU; GPC1; ABR; CYBA; SGSM3; PIGU; PDPK1; TRIP10; CYTH3; PLEKHG2; TAX1BP3; SLC20A1; PLEKHG4; ACTN4; LYPD1; NCLN; SHARPIN; CANT1; WBP2; ATF6B; ATF3 | 3144 | 3139 |
| 9.51E-07 | 0.000155509 | GO:0007010 | b | 4 | cytoskeleton organization | 27; 29; 60; 81; 331; 396; 822; 1263; 1265; 1490; 1627; 2006; 2252; 2275; 2314; 3397; 3636; 3856; 3861; 3875; 3880; 4000; 4130; 4641; 4741; 4926; 5058; 5339; 5879; 5880; 5987; 6464; 6525; 6624; 7070; 7128; 7280; 7791; 22924; 6790; 8558; 9055; 9322; 9828; 10152; 10174; 10376; 10381; 10383; 10432; 10435; 10458; 11135; 11188; 51421; 29984; 84790; 116985; 54461; 54870; 55201; 23122; 57175; 253980; 57606; 91663; 27183; 30846; 5170; 64423; 55616; 64857 | AMOTL2; EHD2; NUMA1; PLK3; MYO1C; CORO1B; TRIP10; XIAP; MYADM; DBN1; PRC1; ABL2; RAC2; SORBS3; ZYX; CLASP2; FBXW5; KRT8; RBM14; INPPL1; NEFM; TUBA1B; CDK10; ARHGEF17; INF2; FHL3; FLII; AURKA; ARHGDIA; PLEKHG2; THY1; NISCH; MAPRE3; QRICH1; MAP1A; ELN; SHC1; RAC1; CDC42EP2; CDC42EP1; TRIM27; TUBB3; ABR; MAP1S; PLEC; VPS4A; PDPK1; FGF7; LMNA; CAPG; TUBB2A; FSCN1; RHOD; KCTD13; ARAP1; ACTN4; ABI2; TUBB4B; CNN2; SMTN; TNFAIP3; ID1; TUBA1C; CTGF; KRT14; SLAIN2; BAIAP2; ACTB; ASAP3; PAK1; KRT19; KRT18 | 1328 | 1326 |
| 1.27E-06 | 0.000173365 | GO:0016477 | b | 4 | cell migration | 29; 81; 309; 65992; 972; 1435; 1464; 1490; 1545; 2022; 2252; 2621; 2817; 2852; 3084; 3162; 3164; 3397; 3675; 3910; 4000; 4192; 4550; 4641; 4681; 4967; 5058; 5176; 5191; 5270; 5327; 5328; 5371; 5420; 5606; 22801; 5879; 5880; 6464; 6624; 6695; 6774; 6793; 7070; 7128; 7763; 8140; 23213; 8482; 8519; 8611; 8613; 8751; 8877; 10014; 10152; 10370; 11188; 51157; 221927; 79143; 51421; 79647; 80270; 57124; 29984; 115908; 23122; 23586; 57175; 57214; 253980; 91663; 26585; 60672; 5170; 55616 | CD74; SPHK1; AMOTL2; HDAC5; STK10; ITGA3; MYO1C; PLPP3; MT-RNR2; GPER1; ZFAND5; ITGA11; MBOAT7; ADAM15; MYADM; RAC2; PML; CD248; MIIP; SERPINE2; CLASP2; MDK; OGDH; CYP1B1; AKIRIN1; CORO1B; NBL1; ZNF580; ENG; SULF1; MAP2K3; CTHRC1; NRG1; CITED2; THY1; NISCH; GREM1; PLPP1; IFITM1; SLC7A5; LAMA4; SHC1; ANXA6; NR4A1; DDX58; PLAU; PLAT; GPC1; ABR; CEMIP; SERPINF1; FGF7; STAT3; LMNA; SEMA7A; BRAT1; FSCN1; RHOD; KCTD13; PODXL; PEX7; CSPG4; ACTN4; ABI2; HMOX1; CSF1; TNFAIP3; ID1; CTGF; HSD3B7; SPOCK1; RAC1; DDRGK1; PDPK1; ASAP3; PAK1; GAS6 | 1468 | 1463 |
| 1.59E-06 | 0.00018554 | GO:0070848 | b | 4 | response to growth factor | 331; 1490; 1848; 2002; 2022; 2064; 2252; 2621; 2817; 3164; 3340; 3371; 3397; 3675; 4641; 4681; 4926; 5179; 5371; 5598; 5606; 6424; 6464; 7041; 7538; 7791; 8200; 23213; 8425; 9076; 9146; 10370; 10938; 84335; 147372; 114990; 118813; 285704; 23770; 57175; 26585; 27429; 53834; 5170; 64855 | FGFRL1; EHD1; NUMA1; ITGA3; MYO1C; FKBP8; MAP2K3; XIAP; GREM1; SULF1; RGMB; ZYX; NDST1; CCBE1; ZFP36; GDF5; AKT1S1; MAPK7; FAM129B; PML; TGFB1I1; DUSP6; PENK; HTRA2; ERBB2; LTBP4; VASN; SHC1; ENG; NR4A1; HGS; GPC1; TNC; CLDN1; FGF7; CORO1B; ZFYVE27; SFRP4; CITED2; ID1; CTGF; ELK1; NBL1; PDPK1; GAS6 | 699 | 697 |
| 1.96E-06 | 0.000200245 | GO:0006915 | b | 4 | apoptotic process | 29; 81; 220; 309; 322; 331; 396; 65992; 467; 598; 972; 1263; 1396; 1490; 1545; 1760; 1848; 2022; 2068; 2621; 2852; 2907; 3162; 3297; 3397; 3429; 3437; 3572; 3575; 3856; 3875; 4000; 4055; 4085; 4192; 4209; 4550; 5055; 5058; 5371; 5598; 5599; 5916; 5978; 6424; 6464; 6774; 6878; 7052; 7128; 7421; 7538; 6790; 8200; 23213; 8751; 8877; 9500; 9531; 9828; 10370; 10514; 10758; 10899; 10922; 10945; 11188; 221927; 400916; 79156; 10280; 84231; 112398; 84335; 57124; 81858; 83596; 51246; 84287; 54867; 55201; 23770; 89941; 26155; 26585; 92140; 27429; 29965; 22822; 5170; 64651; 64782; 64855; 64857 | BCL2L1; HSF1; TGM2; ZDHHC16; NISCH; IL6ST; GPER1; GDF5; FKBP8; LTBR; RARG; APBB1; DMPK; PLK3; SHC1; ARHGEF17; GRINA; IFI27; SERPINB2; BRAT1; MAGED1; CSRNP1; KDELR1; PAK1; IL7R; VDR; MTDH; IFIT3; AEN; ANXA6; CTGF; AKT1S1; FAM129B; TRAF3IP2; MYBBP1A; ENG; MAP1S; STAT3; JTB; EGLN2; HMOX1; TNFAIP3; SHISA5; TMEM214; CRIP1; KRT18; CD74; BAG3; MT-RNR2; XIAP; RHOT2; CHCHD10; KRT8; HTRA2; MDK; CYP1B1; NOC2L; MAPK7; PML; CITED2; MAPK8; DUSP6; TAF6; SFRP4; ID1; ZFP36; CDIP1; SIGMAR1; SPHK1; MAD2L1; ERCC2; ADAM15; CD248; ALDH1A3; PLEKHF1; BCL2L12; PHLDA1; SULF1; FASTK; AURKA; ARHGDIA; GREM1; TRAF7; MEF2D; REST; ABR; LMNA; DDRGK1; PLEKHG2; ACTN4; ATF3; PDPK1; GAS6; SHARPIN | 1933 | 1924 |
| 2.29E-06 | 0.000208199 | GO:0010646 | b | 4 | regulation of cell communication | 29; 81; 331; 396; 65992; 467; 598; 823; 896; 972; 1263; 1388; 1435; 1464; 1490; 1535; 1545; 1627; 1760; 1848; 2022; 2064; 2192; 2252; 2621; 2671; 2817; 2852; 2907; 3084; 3162; 3297; 3340; 3397; 3455; 3481; 3489; 3572; 3575; 3675; 3927; 4000; 4055; 4130; 4192; 4550; 4641; 4681; 4926; 5058; 5127; 5167; 5179; 5270; 5327; 5328; 5371; 5413; 5536; 5598; 5599; 5606; 5879; 5880; 5978; 5998; 6275; 6424; 6447; 6455; 6464; 6574; 6774; 6878; 7041; 7052; 7070; 7128; 7200; 23154; 7867; 6790; 8200; 23213; 8425; 8482; 8558; 8613; 8877; 9146; 9265; 9322; 9454; 9500; 9612; 9828; 10174; 10370; 10435; 10758; 11188; 192669; 400916; 79156; 55738; 84231; 79876; 79971; 84335; 375033; 29984; 147372; 81858; 114990; 115908; 93343; 83596; 116372; 83667; 51246; 116985; 84934; 128869; 54386; 55343; 22848; 23274; 23558; 23641; 23770; 56926; 56928; 57153; 89941; 253980; 25894; 25987; 26119; 91663; 26155; 124583; 26585; 92140; 92799; 27352; 27429; 30851; 5170; 64855; 64857 | BCL2L1; HSF1; TGM2; PEAR1; PLK3; WLS; IL6ST; GPER1; NISCH; GDF5; DBN1; FBLN1; FKBP8; LDOC1; LTBR; NDST1; DMPK; THY1; SCG5; ERBB2; SHC1; ARHGEF17; GRINA; RGS3; ENPP1; TERF2IP; TRAF3IP2; SEMA7A; KCTD13; IFNAR2; CSPG4; MAGED1; CCND3; CSF1; SPPL2B; PAK1; IL7R; TRH; ITGA3; GREM1; ARFGAP1; SEPT5; VASN; MTDH; MAPKAPK3; SERPINE2; GFER; SHKBP1; NBL1; TRAF7; NCOR2; AKT1S1; FAM129B; TGFB1I1; IGF2; MAP1A; ENG; UBA5; IGFBP6; REST; FGF7; STAT3; RHOD; TAF6; PPP5C; HMOX1; AAK1; TNFAIP3; LDLRAP1; SHISA5; DDRGK1; AGO3; GAS6; CD74; SESN2; MYO1C; MT-RNR2; XIAP; RHOT2; CHCHD10; SH3GL1; CYTH3; HTRA2; MDK; CYP1B1; NOC2L; MAPK7; PML; S100A4; MAPK8; DUSP6; PENK; SLC35C1; PLPP3; TSKU; CAPN1; CCBE1; CITED2; LASP1; RITA1; MVB12A; SFRP4; ID1; CTGF; CTHRC1; SPHK1; NUMA1; CLEC16A; MYADM; MAP2K3; SORBS3; PLEKHF1; ARAP1; BCL2L12; CDK10; CDK16; SULF1; AURKA; ARHGDIA; NRG1; HOMER3; SLC44A2; LTBP4; RAC2; RAC1; CDC42EP2; HGS; PLAU; PLAT; GPC1; ABR; CYBA; SGSM3; PIGU; PDPK1; TRIP10; LMNA; PLEKHG2; TAX1BP3; SLC20A1; PLEKHG4; ACTN4; LYPD1; NCLN; SHARPIN; CANT1; NCDN; WBP2; ATF6B; ATF3 | 3496 | 3491 |
| 3.44E-06 | 0.000281459 | GO:2000145 | b | 4 | regulation of cell motility | 27; 29; 81; 65992; 972; 1435; 1545; 2022; 2064; 2192; 2252; 2621; 2852; 3084; 3162; 3675; 3910; 4000; 4641; 4681; 5058; 5176; 5270; 5328; 5420; 5606; 5879; 5880; 6774; 6793; 7070; 23213; 8482; 8519; 8613; 8751; 8877; 10014; 10370; 11188; 51157; 51421; 79647; 29984; 23122; 23586; 57175; 57214; 91663; 26585; 60672; 5170 | CD74; SPHK1; AMOTL2; HDAC5; STK10; ITGA3; MYO1C; PLPP3; GPER1; CORO1B; CEMIP; ADAM15; MYADM; RAC2; FBLN1; ABL2; MIIP; SERPINE2; DDX58; CYP1B1; NBL1; RAC1; SULF1; MAP2K3; NRG1; CITED2; THY1; NISCH; ERBB2; IFITM1; GREM1; LAMA4; CLASP2; ENG; PLAU; ZNF580; ABR; SERPINF1; FGF7; STAT3; LMNA; SEMA7A; RHOD; PODXL; AKIRIN1; ACTN4; HMOX1; CSF1; DDRGK1; PDPK1; PAK1; GAS6 | 881 | 881 |
| 5.80E-06 | 0.000429607 | GO:0051128 | b | 4 | regulation of cellular component organization | 27; 29; 81; 160; 322; 396; 595; 598; 822; 896; 1490; 1535; 1627; 1760; 2006; 2064; 2068; 2192; 2621; 2852; 3159; 3297; 3397; 3481; 3675; 4000; 4085; 4130; 4641; 4651; 4926; 5058; 5167; 5176; 5270; 5371; 5420; 5533; 5599; 5878; 5879; 5880; 5987; 6424; 6455; 6624; 6695; 7070; 7162; 22924; 6790; 8425; 8482; 8507; 8558; 8751; 8877; 9076; 9094; 9133; 7013; 9531; 9810; 10014; 10113; 10152; 10174; 10432; 10435; 10458; 10493; 10801; 10938; 11065; 11135; 221927; 400916; 55738; 112398; 79647; 51548; 146691; 29984; 83667; 116985; 118813; 54386; 54461; 54662; 54732; 54870; 55201; 55357; 22848; 23122; 23274; 23558; 56926; 89941; 57175; 57606; 26119; 91663; 26155; 26585; 27352; 27429; 23381; 30846; 64423; 55616; 64857; 65094 | BCL2L1; HSF1; GPER1; TPBG; DBN1; FBLN1; SMG5; SESN2; APBB1; CLEC16A; DMPK; THY1; SGSM3; MAPRE3; ERBB2; RAB5C; TRIM27; ENPP1; TERF2IP; SEMA7A; BRAT1; PODXL; VAT1; SFRP4; CCND1; CCND3; TERF1; ENC1; PAK1; ITGA3; SLAIN2; GREM1; SEPT9; ARFGAP1; ID1; SERPINE2; CLDN1; QRICH1; IGF2; MAP1A; SIRT6; RNF40; MAP1S; CAPG; RHOD; EGLN2; UBE2C; AAK1; SPOCK1; BAIAP2; ASAP3; GAS6; BAG3; HDAC5; EHD2; EHD1; MYO1C; RHOT2; CHCHD10; ABL2; SH3GL1; INF2; HTRA2; JMJD4; TBC1D2; NOC2L; TMED9; RBM14; PML; MAPK8; FBXW5; ELN; HMGA1; SERPINF1; FSCN1; ZFYVE27; AKIRIN1; PREB; CTGF; TBC1D13; LDLRAP1; MYO10; SPHK1; MAD2L1; NUMA1; ERCC2; MYADM; CORO1B; ADAM15; SORBS3; ARAP1; UNC119; CDK10; AURKA; ARHGDIA; LTBP4; RAC2; CLASP2; RAC1; CDC42EP2; CDC42EP1; ABR; CYBA; AP2A1; LMNA; PLEKHG2; CCNB2; ACTN4; ABI2; NCLN; TOM1L2; WBP2; PPP3CC | 2498 | 2495 |
| 6.82E-06 | 0.000429607 | GO:0016192 | b | 4 | vesicle-mediated transport | 27; 29; 60; 81; 160; 162; 221; 290; 311; 377; 598; 823; 976; 1265; 1535; 2621; 3162; 3481; 3614; 3636; 3671; 3831; 3875; 3953; 4641; 4651; 5058; 5127; 5167; 5328; 5413; 5533; 5709; 5834; 5878; 5879; 5880; 5978; 5987; 6424; 6455; 6793; 6892; 7052; 7867; 8635; 8804; 8907; 9094; 9146; 9230; 9265; 9322; 23406; 9632; 9902; 9961; 10043; 10113; 10312; 10383; 10458; 10493; 10652; 10808; 10938; 10945; 10948; 11070; 55738; 79792; 375033; 93343; 84283; 51150; 84171; 84286; 23265; 118813; 152579; 54732; 26088; 22848; 23122; 23274; 57153; 57222; 26020; 26119; 124583; 26585; 27183; 27352; 64837; 29952; 30846; 5170; 5298; 63910 | BCL2L1; TGM2; SCFD2; PEAR1; STK10; AP1M1; ERGIC1; TMEM175; PI4KB; MRC2; SDF4; GSDMD; COTL1; PYGB; CAPN1; SGSM3; RAB5C; TRIM27; ENPP1; SEC24C; VAT1; CNN2; TUBB4B; ISLR; STARD3; ACTB; KDELR1; PAK1; ALDH3B1; ARFGAP1; SEPT5; MAPKAPK3; TMEM115; HSPH1; SH3GL1; TMEM79; RAB11B; ANXA11; IGF2; REST; KLC1; KLC2; GAS6; HMOX1; AAK1; TCIRG1; ANPEP; BAIAP2; KRT18; EHD2; EHD1; MYO1C; ABL2; RNASET2; ARF3; TMED9; MVP; YKT6; LEPR; GGA1; VPS4A; MVB12A; ZFYVE27; SLC17A9; SFRP4; PREB; TAPBP; LDLRAP1; MYO10; CREG1; ADGRE5; TOM1; CLEC16A; PSMD3; LOXL4; INPPL1; UNC119; CDK16; IMPDH1; AP1B1; DPP7; LRP10; SLC44A2; GREM1; RAC2; CLASP2; RAC1; HGS; PLAU; ABR; CYBA; AP2A1; TRIP10; CYTH3; EXOC7; ACTN4; CANT1; PDPK1; PPP3CC | 2125 | 2118 |
| 6.83E-06 | 0.000429607 | GO:0034613 | b | 4 | cellular protein localization | 60; 160; 162; 377; 65992; 598; 972; 1263; 1384; 1627; 2064; 2252; 2621; 2852; 3397; 3429; 3434; 3675; 3875; 4000; 4130; 4641; 4926; 23383; 5058; 5191; 5371; 5599; 5878; 5880; 5905; 6192; 6447; 6449; 6774; 7070; 7128; 22924; 6790; 8086; 8266; 8733; 8907; 9146; 9230; 7013; 9454; 9531; 9632; 9697; 10043; 10113; 10938; 10945; 51114; 51172; 400916; 79156; 79971; 146691; 29984; 116372; 83667; 51608; 84885; 79711; 128869; 118813; 54386; 54662; 54732; 26088; 55357; 23122; 23511; 57175; 57214; 26119; 91663; 26262; 27183; 27352; 27429; 23381; 29927; 30846; 30851; 5170; 65083 | BCL2L1; AAAS; ZDHHC12; PLK3; WLS; TSPAN17; GPER1; DBN1; SMG5; SESN2; THY1; SCG5; MAPRE3; ERBB2; AP1M1; RAB5C; RANGAP1; IFI27; TERF2IP; SEC24C; HOMER3; TERF1; ACTB; KDELR1; PAK1; ITGA3; MYADM; ID1; IFIT1; RAB11B; MAP1A; MAU2; SGTA; FGF7; GET4; RHOD; PEX7; ZDHHC9; TNFAIP3; DDRGK1; AP2A1; IPO4; GAS6; CD74; BAG3; EHD2; EHD1; UBL4A; MYO1C; CHCHD10; HTRA2; RPS4Y1; STAT3; ARF3; TBC1D2; TMED9; PML; MAPK8; GGA1; VPS4A; NOL6; ZFYVE27; PREB; TRAM2; GPAA1; TBC1D13; LDLRAP1; NAGPA; CEMIP; NUMA1; TOM1; CORO1B; PLEKHF1; LYPD1; NUP188; AURKA; AP1B1; RAC2; CLASP2; HGS; SGSM3; PIGU; LMNA; CRAT; TAX1BP3; TOM1L2; PDPK1; KRT18; SEC61A1 | 1851 | 1848 |
| 8.77E-06 | 0.000488914 | GO:0016032 | b | 4 | viral process | 160; 162; 290; 598; 972; 2068; 2192; 2621; 3159; 3429; 3431; 3434; 3572; 3831; 3856; 3875; 3880; 4055; 5371; 5696; 5879; 5978; 5987; 6449; 6464; 6575; 6774; 7538; 7791; 8086; 8519; 8907; 9076; 10152; 10581; 79080; 93343; 54442; 54587; 23511; 23586; 23770; 90480; 27183 | BCL2L1; CD74; AAAS; KCTD5; ERCC2; AP1M1; IL6ST; FBLN1; KRT8; FKBP8; IFIT1; LTBR; DDX58; MVB12A; STAT3; GADD45GIP1; NUP188; ZYX; PML; CLDN1; AP1B1; IFI27; IFITM2; IFITM1; SHC1; RAC1; TRIM27; REST; HMGA1; SP110; AP2A1; VPS4A; MXRA8; SGTA; SLC20A2; ABI2; CCDC86; ZFP36; ANPEP; PSMB8; KLC1; GAS6; KRT19; KRT18 | 721 | 721 |
| 8.97E-06 | 0.000488914 | GO:0051270 | b | 4 | regulation of cellular component movement | 27; 29; 81; 65992; 476; 972; 1435; 1545; 2022; 2064; 2192; 2252; 2621; 2852; 3084; 3162; 3675; 3910; 4000; 4641; 4681; 5058; 5176; 5270; 5328; 5420; 5606; 5879; 5880; 6774; 6793; 7070; 23213; 8482; 8519; 8613; 8751; 8877; 10014; 10370; 11188; 51157; 51421; 79647; 29984; 116985; 23122; 23586; 57175; 57214; 91663; 26585; 60672; 5170 | CD74; SPHK1; AMOTL2; HDAC5; STK10; ITGA3; MYO1C; PLPP3; GPER1; CORO1B; CEMIP; ADAM15; MYADM; RAC2; FBLN1; ABL2; MIIP; SERPINE2; DDX58; CYP1B1; ARAP1; NBL1; RAC1; SULF1; MAP2K3; NRG1; CITED2; THY1; NISCH; ERBB2; IFITM1; GREM1; LAMA4; CLASP2; ENG; PLAU; ZNF580; ABR; SERPINF1; FGF7; STAT3; LMNA; SEMA7A; RHOD; PODXL; AKIRIN1; ACTN4; HMOX1; CSF1; ATP1A1; DDRGK1; PDPK1; PAK1; GAS6 | 960 | 960 |
| 1.31E-05 | 0.000669199 | GO:0051017 | b | 4 | actin filament bundle assembly | 81; 1490; 2006; 3397; 5058; 5879; 6624; 7791; 10174; 10458; 29984; 116985; 23122; 57175; 253980; 55616 | KCTD13; ELN; CLASP2; ARAP1; RAC1; ACTN4; ID1; CORO1B; CTGF; ZYX; BAIAP2; SORBS3; ASAP3; PAK1; FSCN1; RHOD | 150 | 150 |
| 1.52E-05 | 0.000730327 | GO:0036293 | b | 4 | response to decreased oxygen levels | 81; 1263; 1490; 1535; 2068; 3162; 3297; 3776; 4000; 4313; 4540; 5058; 5179; 5327; 5328; 5371; 5978; 7200; 8751; 10370; 112398; 29988; 114990; 22937; 64428 | HSF1; PLK3; ADAM15; SCAP; MT-ND5; KCNK2; NARFL; PML; CITED2; PENK; ERCC2; TRH; VASN; REST; PLAT; CYBA; PLAU; LMNA; EGLN2; ACTN4; SLC2A8; HMOX1; CTGF; MMP2; PAK1 | 320 | 319 |
| 1.71E-05 | 0.00077602 | GO:0034097 | b | 4 | response to cytokine | 81; 445; 471; 595; 598; 972; 1265; 1435; 1535; 2621; 2852; 3162; 3429; 3434; 3437; 3455; 3572; 3575; 3590; 3636; 3726; 3856; 3875; 3953; 4055; 4313; 4502; 4641; 5055; 5118; 5371; 5598; 5599; 5696; 5916; 55627; 6464; 6624; 6774; 6778; 7128; 7538; 7791; 7867; 8519; 8877; 9076; 9536; 10376; 10435; 10437; 10581; 10678; 81858; 23586; 56928; 26119; 27429; 29927; 29965 | BCL2L1; CD74; SPHK1; MT2A; IFI27; JUNB; MYO1C; IFITM1; IL6ST; IFNAR2; SMPD4; HTRA2; ZYX; KRT8; IFIT3; IFIT1; LTBR; STAT6; DDX58; STAT3; INPPL1; ASS1; MAPK7; CDC42EP2; PCOLCE; CLDN1; MAPK8; IFITM2; MAPKAPK3; SHC1; TUBA1B; IL7R; B3GNT2; IFI30; LEPR; PML; CYBA; PTGES; SERPINB2; FSCN1; GPER1; ATIC; RARG; ACTN4; CCND1; CNN2; HMOX1; CSF1; TNFAIP3; SEC61A1; ZFP36; SPPL2B; PSMB8; IL11RA; CDIP1; KRT18; MMP2; LDLRAP1; SHARPIN; GAS6 | 1133 | 1132 |
| 2.74E-05 | 0.001177679 | GO:0048522 | b | 4 | positive regulation of cellular process | 27; 29; 81; 160; 220; 322; 331; 396; 445; 65992; 467; 595; 598; 822; 823; 896; 972; 1263; 1388; 1435; 1464; 1490; 1535; 1545; 1627; 1848; 1889; 2002; 2022; 2064; 2068; 2192; 2252; 2621; 2817; 2852; 3084; 3159; 3162; 3164; 3297; 3340; 3371; 3397; 3398; 3481; 3572; 3575; 3590; 3675; 3726; 3776; 3927; 4000; 4055; 4085; 4192; 4209; 4313; 4641; 4665; 4681; 4782; 4926; 5058; 5118; 5176; 5270; 5328; 5371; 5420; 5533; 5536; 5598; 5599; 5606; 5879; 5880; 5916; 5978; 5987; 6199; 6275; 6300; 55859; 6424; 6449; 6455; 6464; 6574; 6601; 6624; 6774; 6778; 6793; 7030; 7041; 7052; 7070; 7128; 7162; 7200; 7421; 7538; 7867; 22924; 6790; 8078; 8200; 23213; 8482; 8507; 8519; 8558; 8613; 8877; 9055; 9076; 9094; 9133; 9146; 7013; 9500; 9531; 9810; 9828; 10014; 10025; 10152; 10174; 10312; 10370; 10432; 10435; 10458; 10514; 10758; 10801; 10808; 10899; 10938; 11065; 338773; 10985; 11135; 192669; 51157; 221927; 400916; 79156; 84231; 79647; 79792; 51548; 79971; 112770; 57060; 57124; 29984; 147372; 81858; 84836; 115908; 83596; 83667; 51246; 51334; 116985; 118813; 54386; 54732; 54962; 22848; 22937; 23122; 23274; 80381; 23558; 285704; 23586; 23640; 23764; 23770; 57153; 57175; 57214; 253980; 57606; 91304; 26073; 26119; 91663; 124583; 26585; 92140; 27183; 92799; 27429; 30827; 30846; 5170; 64651; 64855; 64857; 65094 | BCL2L1; HSF1; TGM2; PLK3; WLS; FAM129B; IL6ST; GPER1; TPBG; GDF5; DBN1; FBLN1; ASS1; MAFF; LTBR; SESN2; RARG; NDST1; GSDMD; APBB1; CAPN1; THY1; STK10; MAPRE3; ERBB2; IFITM1; SHC1; ARHGEF17; TRIM27; TERF2IP; CLDN1; SEMA7A; BRAT1; KCTD13; PODXL; AKIRIN1; CSPG4; CCND1; MAGED1; CCND3; CSF1; ELK1; PRR16; ENC1; CXXC1; PAK1; RNF40; IL7R; VDR; ITGA3; TMEM119; SEPT9; RGMB; MYADM; MTDH; MAPKAPK3; SERPINE2; DDX58; HSPH1; NBL1; TRAF7; ZFP36; PCBP4; TERF1; TFE3; SULF1; PCOLCE; TGFB1I1; MYBBP1A; IGF2; TRH; SIRT6; GLMP; ENG; TRAF3IP2; SGTA; ZNF580; REST; FGF7; STAT3; HGS; CAPG; JTB; CSRNP1; GAS6; PPP5C; UBE2C; CD276; HMOX1; AAK1; TNFAIP3; LDLRAP1; TCIRG1; SHISA5; MEF2D; IL11RA; BAIAP2; AGO3; NAB2; CD74; BAG3; HDAC5; EHD2; EHD1; MYO1C; FKBP8; DDRGK1; RHOD; XIAP; CEMIP; MAPK12; CHCHD10; ABL2; SH3GL1; SHKBP1; HTRA2; MDK; CYP1B1; JMJD4; POLDIP2; TMED9; RPS6KB2; MAPK7; RBM14; PML; S100A4; MAPK8; DUSP6; CLEC16A; PLEKHG2; PLPP3; SMARCC2; CCBE1; HMGA1; GCN1; SERPINF1; VPS4A; CITED2; LASP1; FSCN1; CORO1B; ZFYVE27; SFRP4; SLAIN2; ID2; ID1; CTGF; CTHRC1; SPHK1; MAD2L1; TMEM259; NUMA1; ERCC2; JUNB; USP5; MAP2K3; ECE1; SCAP; PRC1; CD248; SORBS3; HSPBP1; ALDH1A3; PLEKHF1; ARAP1; BCL2L12; UNC119; CDK10; KCNK2; AURKA; MED16; ARHGDIA; NRG1; SLC44A2; GREM1; RAC2; CLASP2; BEX1; RAC1; CDC42EP2; CDC42EP1; NR4A1; STAT6; PLAU; ABHD14B; GPC1; ABR; CYBA; AP2A1; TNC; PDPK1; LMNA; NFIC; CCNB2; SLC20A1; ACTN4; ABI2; TIPIN; SHARPIN; CANT1; WBP2; ATF6B; PPP3CC; MMP2; ATF3 | 5324 | 5307 |
| 4.81E-05 | 0.001950547 | GO:0001649 | b | 4 | osteoblast differentiation | 1291; 3371; 3397; 3398; 3481; 3572; 3726; 4209; 5179; 22801; 5978; 8482; 8519; 9902; 10514; 338773; 115908; 26585 | IGF2; IFITM1; GREM1; SEMA7A; JUNB; ID2; MRC2; REST; IL6ST; TMEM119; ITGA11; TNC; MEF2D; COL6A1; ID1; PENK; CTHRC1; MYBBP1A | 203 | 203 |
| 5.01E-05 | 0.001950547 | GO:0032870 | b | 4 | cellular response to hormone stimulus | 445; 65992; 476; 896; 1535; 196883; 2002; 2852; 3164; 3297; 3371; 3481; 3726; 3953; 4641; 5058; 5167; 5176; 5905; 5916; 5978; 6464; 6774; 7041; 7200; 7421; 7538; 8611; 9612; 10014; 10025; 10312; 10432; 10458; 112398; 79876; 29988; 116372; 23558; 155066; 5170 | HSF1; PLPP1; VDR; HDAC5; JUNB; MYO1C; GPER1; ASS1; NCOR2; STAT3; RARG; LYPD1; PDPK1; MED16; RBM14; TGFB1I1; IGF2; TRH; SHC1; UBA5; NR4A1; RANGAP1; REST; LEPR; CYBA; SERPINF1; TNC; WBP2; ENPP1; ADCY4; EGLN2; SLC2A8; CCND3; TCIRG1; ZFP36; ELK1; ATP1A1; DDRGK1; BAIAP2; ATP6V0E2; PAK1 | 705 | 703 |
| 6.63E-05 | 0.002463345 | GO:0072359 | b | 4 | circulatory system development | 162; 290; 1396; 1464; 1490; 1545; 1848; 1889; 2006; 2022; 2064; 2852; 2969; 3084; 3162; 3164; 3340; 3397; 3398; 3675; 3726; 3776; 3953; 4000; 4209; 4313; 4522; 5058; 5176; 5371; 5598; 5978; 6050; 6464; 6774; 7070; 7128; 7763; 23213; 8751; 8877; 9146; 10014; 1717; 10370; 51421; 51548; 147372; 84287; 26585; 92140; 53834; 5170; 64855 | SPHK1; AMOTL2; HDAC5; ID2; ITGA3; FGFRL1; DHCR7; GPER1; ZFAND5; ZDHHC16; RNH1; ECE1; MTDH; SIRT6; CYP1B1; NDST1; CCBE1; FAM129B; KCNK2; MAPK7; SULF1; NRG1; CITED2; AP1B1; GTF2I; GREM1; ERBB2; JUNB; ELN; SHC1; ENG; NR4A1; HGS; REST; LEPR; PML; SERPINF1; STAT3; LMNA; THY1; DUSP6; MTHFD1; CSPG4; MMP2; HMOX1; TNFAIP3; ID1; CTGF; ANPEP; MEF2D; PDPK1; CRIP1; PAK1; ADAM15 | 1033 | 1033 |
| 8.05E-05 | 0.002863333 | GO:0007015 | b | 4 | actin filament organization | 81; 822; 1490; 1627; 2006; 3397; 3636; 4641; 5058; 5879; 5880; 5987; 6624; 7791; 10152; 10174; 10435; 10458; 11135; 29984; 116985; 23122; 57175; 253980; 91663; 55616; 64857 | MYO1C; CORO1B; MYADM; DBN1; SORBS3; RAC2; ZYX; ARAP1; PLEKHG2; ELN; CLASP2; RAC1; CDC42EP2; CDC42EP1; TRIM27; CAPG; FSCN1; RHOD; KCTD13; INPPL1; ACTN4; ABI2; ID1; CTGF; BAIAP2; PAK1; ASAP3 | 397 | 396 |
| 9.02E-05 | 0.002970482 | GO:0032970 | b | 4 | regulation of actin filament-based process | 27; 396; 476; 822; 1265; 1490; 2006; 3397; 4641; 5058; 5879; 5987; 6624; 8558; 10152; 10174; 10435; 10458; 11135; 29984; 116985; 23122; 57175; 91663; 55616; 64857 | MYO1C; CORO1B; MYADM; SORBS3; ABL2; ARAP1; CDK10; ARHGDIA; ELN; CLASP2; RAC1; CDC42EP2; CDC42EP1; TRIM27; CAPG; FSCN1; RHOD; PLEKHG2; CNN2; ABI2; ID1; CTGF; ATP1A1; BAIAP2; PAK1; ASAP3 | 377 | 377 |
| 9.08E-05 | 0.002970482 | GO:0051094 | b | 4 | positive regulation of developmental process | 27; 160; 322; 396; 595; 972; 1435; 1490; 1545; 1627; 2022; 2252; 2621; 2817; 2852; 3084; 3159; 3162; 3297; 3398; 3481; 3572; 3575; 3675; 3726; 4000; 4681; 4926; 5058; 5176; 5270; 5879; 5978; 6300; 6424; 6774; 6778; 7041; 7128; 7162; 7421; 7538; 6790; 8200; 8482; 8507; 8519; 8877; 9500; 10370; 10938; 338773; 79647; 51548; 115908; 84283; 118813; 23122; 57175; 91663; 26585; 92140; 30846; 5170; 64855 | CD74; HSF1; SPHK1; VDR; EHD2; EHD1; NUMA1; HMOX1; ITGA3; IL6ST; GPER1; TPBG; GREM1; MAPK12; MYADM; DBN1; ABL2; SERPINE2; CLASP2; STAT6; MTDH; CYP1B1; NBL1; RAC1; APBB1; GDF5; TMEM79; PDPK1; AURKA; ARHGDIA; NRG1; TGFB1I1; ZFP36; IGF2; IFITM1; JUNB; SIRT6; ENG; IL7R; FAM129B; REST; HMGA1; GPC1; AP2A1; CITED2; FGF7; STAT3; LMNA; SEMA7A; CORO1B; ZFYVE27; AKIRIN1; SFRP4; TMEM119; CCND1; MAGED1; ID2; CSF1; TNFAIP3; CTHRC1; CTGF; ENC1; SERPINF1; PAK1; GAS6 | 1333 | 1331 |
| 0.000114431 | 0.003572848 | GO:0022603 | b | 4 | regulation of anatomical structure morphogenesis | 29; 81; 160; 396; 1435; 1545; 1627; 2022; 2064; 2192; 2252; 2969; 3162; 3397; 4651; 5058; 5176; 5371; 5598; 5879; 6050; 6774; 7070; 7128; 7421; 23213; 8482; 8877; 9146; 9500; 10014; 10370; 10435; 10458; 10493; 10938; 11135; 51548; 29984; 115908; 116985; 118813; 54870; 23122; 57175; 91663; 26585; 92140; 30846; 5170; 64423; 64855 | SPHK1; VDR; HDAC5; EHD2; EHD1; CORO1B; RNH1; MYADM; DBN1; FBLN1; MTDH; INF2; CLASP2; CYP1B1; ARAP1; RAC1; SULF1; PDPK1; MAPK7; ARHGDIA; PML; CITED2; THY1; GTF2I; QRICH1; ERBB2; GREM1; SIRT6; ENG; CDC42EP2; CDC42EP1; FAM129B; HGS; ABR; CTHRC1; SERPINF1; BAIAP2; FGF7; STAT3; SEMA7A; RHOD; VAT1; ZFYVE27; ACTN4; MAGED1; HMOX1; CSF1; TNFAIP3; ID1; AP2A1; PAK1; MYO10 | 1005 | 1004 |
| 0.00011793 | 0.003572848 | GO:0040013 | b | 4 | negative regulation of locomotion | 29; 1545; 2022; 2192; 3084; 3162; 4681; 5176; 5371; 5987; 6774; 7070; 23213; 8482; 8519; 8751; 10014; 10370; 11188; 23122; 57175; 26585; 60672 | HDAC5; NISCH; CORO1B; ADAM15; FBLN1; MIIP; CYP1B1; NBL1; SULF1; PML; CITED2; THY1; IFITM1; GREM1; CLASP2; ENG; TRIM27; NRG1; ABR; SERPINF1; STAT3; SEMA7A; HMOX1 | 319 | 319 |
| 0.00015148 | 0.004425366 | GO:0090287 | b | 4 | regulation of cellular response to growth factor stimulus | 331; 2022; 2817; 3675; 4641; 4681; 4926; 6424; 7041; 8200; 23213; 8425; 9146; 10370; 147372; 114990; 23770; 26585; 5170; 64855 | CCBE1; LTBP4; VASN; SFRP4; ENG; NBL1; ITGA3; MYO1C; HGS; GDF5; GPC1; XIAP; GREM1; NUMA1; SULF1; FAM129B; CITED2; TGFB1I1; PDPK1; FKBP8 | 262 | 262 |
| 0.000173833 | 0.004903284 | GO:0030198 | b | 4 | extracellular matrix organization | 822; 1291; 1490; 1545; 2006; 2022; 2068; 2192; 2621; 3371; 3675; 3910; 4313; 4320; 22801; 6624; 7373; 8076; 23213; 8751; 133584; 84171; 23122; 26585 | EGFLAM; MMP11; ERCC2; ITGA3; MFAP5; ITGA11; ADAM15; LAMA4; FBLN1; LOXL4; SULF1; GREM1; ELN; CLASP2; ENG; TNC; CYP1B1; CAPG; FSCN1; COL14A1; CTGF; COL6A1; MMP2; GAS6 | 349 | 349 |
| 0.000180661 | 0.004926016 | GO:0043620 | b | 4 | regulation of DNA-templated transcription in response to stress | 467; 1388; 3162; 3297; 5598; 9531; 10370; 10985; 112398; 83667 | HSF1; BAG3; SESN2; EGLN2; HMOX1; GCN1; MAPK7; CITED2; ATF6B; ATF3 | 82 | 82 |
| 0.000198097 | 0.005227194 | GO:0048545 | b | 4 | response to steroid hormone | 445; 65992; 476; 595; 1490; 1535; 2022; 2852; 3164; 4192; 5058; 5176; 5916; 5978; 7041; 7200; 7421; 7538; 8611; 9076; 9612; 10025; 10432; 112398; 79876; 23558 | VDR; GPER1; ASS1; NCOR2; MDK; RARG; CTGF; MED16; RBM14; CLDN1; PLPP1; TRH; ENG; NR4A1; REST; CYBA; SERPINF1; TGFB1I1; EGLN2; CCND1; ZFP36; UBA5; ATP1A1; DDRGK1; WBP2; PAK1 | 396 | 396 |
| 0.000256484 | 0.006556364 | GO:0036211 | b | 4 | protein modification process | 27; 60; 322; 331; 65992; 467; 595; 896; 972; 1263; 1435; 1464; 1490; 1760; 1801; 1802; 1848; 2022; 2064; 2068; 2134; 2192; 2252; 2590; 2621; 2852; 3084; 3297; 3340; 3371; 3397; 3429; 3481; 3572; 4000; 4055; 4085; 4245; 4665; 4967; 5058; 5127; 5167; 5179; 5327; 5371; 5531; 5533; 5536; 27315; 5598; 5599; 5606; 5696; 5709; 5879; 5880; 5978; 5987; 5998; 6199; 6300; 6464; 6484; 6774; 6793; 7052; 7064; 7070; 7128; 7538; 7867; 22924; 6790; 7991; 8078; 8200; 8266; 8460; 8482; 8507; 8558; 8611; 8613; 8733; 8877; 9094; 9133; 9146; 9230; 140459; 9500; 9810; 9961; 10014; 10152; 10174; 10432; 10489; 10612; 10678; 10899; 10922; 11065; 11070; 10985; 11176; 11285; 11313; 51114; 51172; 143888; 221927; 5891; 79102; 84231; 112398; 79876; 79903; 51548; 84335; 133584; 81858; 83667; 51200; 51246; 84171; 84287; 84885; 128869; 348180; 54386; 54461; 54587; 54681; 55214; 55343; 22848; 23558; 23586; 23640; 23770; 57214; 253980; 91663; 26133; 26155; 26262; 26585; 27090; 60509; 60681; 29089; 26090; 30827; 5170; 63891; 64840; 65094 | HSF1; TGM2; PGAP2; FKBP10; TUSC3; PLK3; IL6ST; GPER1; ZDHHC16; ABHD12; NAA60; FBLN1; FKBP8; LTBR; CTU2; OGDH; ASB6; B3GNT2; NDST1; PPP4C; ZDHHC12; APBB1; AKT1S1; P3H2; DMPK; THY1; IFI27; MAPRE3; ERBB2; SHC1; EXTL1; TRIM27; ENPP1; TERF2IP; TSPAN17; SEMA7A; BRAT1; KCTD13; LRRC41; CSPG4; CCND1; MAGED1; CCND3; CSF1; ENC1; ACTB; PAK1; EGFLAM; KDELC2; CXXC1; TRAF7; MYADM; BAZ2A; MAPKAPK3; TMEM115; DDX58; TPST1; CTGF; CPA4; RAB11B; B4GALT7; IGF2; SIRT6; ENG; RNF40; P4HTM; UBA5; FGF7; PLAT; GALNT2; JTB; EGLN2; LYPLA2; GAS6; ZDHHC9; PPP5C; UBE2C; TNFAIP3; MOK; SHISA5; STK10; DDRGK1; TRIM3; RNF123; UBE2T; NAB2; CD74; AGBL5; HDAC5; SESN2; UBL4A; NAGPA; XIAP; CEMIP; MAPK12; ABL2; STAT3; JMJD4; NOC2L; RPS6KB2; MAPK7; RBM14; PML; MAPK8; DUSP6; PENK; FBXW5; SLC35C1; PLPP1; PLPP3; CCNB2; GCN1; MXRA8; RNF26; PORCN; ID1; ZFP36; GPAA1; ST3GAL4; PSMB8; TRPC4AP; MVP; SPHK1; MAD2L1; ERCC2; USP5; PSMD3; MAP2K3; SORBS3; HSPBP1; LOXL4; UNC119; CDK10; CDK16; GDF5; FASTK; AURKA; RGS3; NRG1; GREM1; RAC2; RAC1; HGS; REST; THOP1; MGAT1; AAK1; PIGU; TNC; PDPK1; LMNA; ST6GALNAC4; DPH1; DPH2; ABI2; SHARPIN; WBP2; PPP3CC; ATF3 | 4097 | 4089 |
| 0.000285488 | 0.007076641 | GO:0022612 | b | 4 | gland morphogenesis | 823; 1396; 1435; 2252; 3371; 5371; 5916; 6778; 7052; 7128; 7421; 23213 | STAT6; TGM2; VDR; CAPN1; RARG; CSF1; TNFAIP3; PML; SULF1; TNC; FGF7; CRIP1 | 120 | 120 |
| 0.000306256 | 0.007328839 | GO:0023056 | b | 4 | positive regulation of signaling | 81; 331; 65992; 467; 598; 972; 1435; 1464; 1490; 1535; 1545; 1848; 1889; 2022; 2064; 2252; 2621; 2852; 3084; 3162; 3297; 3340; 3481; 3572; 3575; 3927; 4055; 4641; 4926; 5058; 5270; 5371; 5536; 5599; 5606; 5879; 5880; 6275; 6424; 6464; 6574; 6774; 7041; 7052; 7128; 7200; 7867; 8200; 23213; 8482; 8558; 8877; 9500; 10174; 10370; 10758; 192669; 400916; 79156; 84231; 79971; 147372; 81858; 51246; 116985; 54386; 22848; 23274; 23558; 23770; 57153; 253980; 26119; 124583; 26585; 92140; 92799; 27429 | BCL2L1; CD74; TGM2; NUMA1; SHARPIN; CLEC16A; WLS; MYO1C; SPHK1; FKBP8; IL6ST; GPER1; LASP1; XIAP; SULF1; ECE1; CHCHD10; SORBS3; MTDH; IGF2; SHKBP1; SERPINE2; LTBR; CCBE1; CYP1B1; SFRP4; ARAP1; CITED2; TRAF7; NDST1; CDK10; RAC1; GDF5; SLC44A2; HTRA2; NRG1; S100A4; MAPK8; DUSP6; ERBB2; TRH; MAPKAPK3; GREM1; RAC2; SHC1; ENG; TRAF3IP2; IL7R; PML; AAK1; CYBA; TERF2IP; TGFB1I1; FGF7; STAT3; PLEKHF1; SEMA7A; KCTD13; MAP2K3; SLC20A1; CSPG4; HSF1; ACTN4; PPP5C; HMOX1; CSF1; TNFAIP3; LDLRAP1; SHISA5; CTGF; AGO3; CANT1; DDRGK1; WBP2; MAGED1; PAK1; ATF3; GAS6 | 1748 | 1745 |
| 0.000313581 | 0.007328839 | GO:0030258 | b | 4 | lipid modification | 37; 1384; 2064; 2252; 3084; 3628; 3636; 5191; 5879; 5880; 6484; 8611; 8613; 8871; 8877; 83667; 55343; 27349; 126129; 5298; 64748 | CRAT; ERBB2; PLPP3; ACADVL; RAC2; PEX7; INPPL1; RAC1; PLPP1; ST3GAL4; SPHK1; CPT1C; NRG1; INPP1; PI4KB; SYNJ2; FGF7; MCAT; SESN2; PLPPR2; SLC35C1 | 299 | 298 |
| 0.000356983 | 0.008111458 | GO:0048705 | b | 4 | skeletal system morphogenesis | 309; 1291; 1490; 3340; 3636; 4209; 4313; 4522; 4665; 5191; 5916; 6424; 7059; 7373; 7763; 29985; 26585; 64651 | SLC39A3; SFRP4; MTHFD1; INPPL1; ANXA6; NDST1; RARG; COL14A1; THBS3; CSRNP1; PEX7; CTGF; GREM1; MEF2D; COL6A1; ZFAND5; MMP2; NAB2 | 239 | 238 |
| 0.000369967 | 0.00817928 | GO:0048514 | b | 4 | blood vessel morphogenesis | 290; 1464; 1490; 1545; 2022; 2064; 2969; 3162; 3164; 3397; 3726; 3953; 4313; 5176; 5371; 5598; 6050; 6464; 6774; 7070; 7128; 23213; 8751; 8877; 9146; 10014; 51421; 51548; 147372; 26585; 92140; 5170; 64855 | SPHK1; AMOTL2; HDAC5; JUNB; RNH1; MTDH; SIRT6; CYP1B1; CCBE1; SULF1; MAPK7; FAM129B; PML; THY1; GTF2I; ERBB2; GREM1; SHC1; ENG; NR4A1; HGS; LEPR; SERPINF1; STAT3; CSPG4; HMOX1; TNFAIP3; ID1; CTGF; ANPEP; PDPK1; MMP2; ADAM15 | 576 | 576 |
| 0.000395357 | 0.008510586 | GO:0048732 | b | 4 | gland development | 220; 445; 595; 823; 1396; 1435; 2252; 3084; 3162; 3371; 3398; 3481; 4192; 5176; 5270; 5371; 5916; 6778; 7052; 7128; 7421; 6790; 23213; 9076; 9133; 10370; 79971 | TGM2; VDR; ID2; WLS; ASS1; SERPINE2; ALDH1A3; MDK; RARG; SULF1; AURKA; CAPN1; PML; CLDN1; IGF2; STAT6; NRG1; SERPINF1; TNC; CITED2; FGF7; CCNB2; CCND1; HMOX1; CSF1; TNFAIP3; CRIP1 | 437 | 437 |
| 0.000421763 | 0.008846203 | GO:0044267 | b | 4 | cellular protein metabolic process | 27; 60; 10939; 160; 322; 331; 65992; 467; 595; 896; 972; 1263; 1435; 1464; 1490; 1760; 1801; 1802; 1848; 2022; 2064; 2068; 2134; 2192; 2252; 2590; 2621; 2687; 2852; 3084; 3297; 3340; 3371; 3397; 3429; 3481; 3489; 3572; 4000; 4055; 4085; 4130; 4245; 4313; 4665; 4967; 5055; 5058; 5118; 5127; 5167; 5176; 5179; 5270; 5327; 5371; 5531; 5533; 5536; 27315; 5598; 5599; 5606; 5696; 5709; 5879; 5880; 5978; 5987; 5998; 6192; 6199; 6300; 6449; 6464; 6484; 6695; 6774; 6793; 7041; 7052; 7064; 7070; 7128; 7538; 7867; 22924; 6790; 7991; 8078; 8200; 8266; 8460; 8482; 8507; 8558; 8611; 8613; 8722; 8733; 8877; 9094; 9133; 9146; 9230; 140459; 9500; 9810; 9961; 10014; 10152; 10174; 10312; 10432; 10489; 10612; 10678; 10899; 10922; 11065; 11070; 10985; 11176; 11285; 11313; 192669; 51114; 51172; 51204; 143888; 221927; 5891; 79102; 84231; 112398; 79876; 79903; 51548; 84335; 133584; 147372; 81858; 93343; 83596; 83667; 51200; 51246; 51334; 84171; 84287; 51608; 84885; 128869; 348180; 54386; 54442; 54461; 54587; 54681; 54938; 55214; 26088; 55343; 22848; 23274; 23558; 23586; 23640; 23770; 57214; 253980; 90480; 91304; 91663; 26133; 26155; 26164; 26262; 26585; 92399; 27090; 27183; 27429; 60509; 60681; 29089; 26090; 30827; 5170; 63891; 64840; 65094 | HSF1; TGM2; PGAP2; FKBP10; TUSC3; PLK3; TACO1; IL6ST; GPER1; AFG3L2; ABHD12; NAA60; FBLN1; FKBP8; LTBR; CTU2; OGDH; MVB12A; ASB6; B3GNT2; NDST1; PPP4C; ZDHHC12; APBB1; CLEC16A; AKT1S1; P3H2; DMPK; THY1; IFI27; MAPRE3; ERBB2; SHC1; EXTL1; TRIM27; CTSF; ENPP1; TERF2IP; SERPINB2; SEMA7A; BRAT1; KCTD13; LRRC41; MRRF; CSPG4; CCND1; MAGED1; CCND3; CSF1; GADD45GIP1; PRR16; ENC1; ACTB; PAK1; RNF40; EGFLAM; KDELC2; CXXC1; TRAF7; MYADM; TSPAN17; BAZ2A; MAPKAPK3; TMEM115; SERPINE2; DDX58; ST3GAL4; CTGF; CPA4; RAB11B; PCOLCE; TGFB1I1; B4GALT7; IGF2; MAP1A; SIRT6; ENG; SGTA; P4HTM; UBA5; IGFBP6; FGF7; PLAT; GET4; GALNT2; JTB; ZDHHC16; EGLN2; LYPLA2; GAS6; ZDHHC9; PPP5C; UBE2C; TNFAIP3; TCIRG1; SHISA5; STK10; SPOCK1; DDRGK1; TRIM3; RNF123; UBE2T; AGO3; NAB2; CD74; AGBL5; HDAC5; SESN2; UBL4A; NAGPA; XIAP; CEMIP; MAPK12; ABL2; HTRA2; RPS4Y1; STAT3; TMEM259; JMJD4; NOC2L; MTG2; RPS6KB2; MAPK7; RBM14; PML; MAPK8; DUSP6; PENK; FBXW5; SLC35C1; PLPP1; PLPP3; CCNB2; GGA1; GCN1; SERPINF1; VPS4A; MXRA8; RNF26; PORCN; ID1; ZFP36; GPAA1; PSMB8; TRPC4AP; SARS2; MVP; SPHK1; MAD2L1; KCTD5; ERCC2; USP5; PSMD3; MAP2K3; SORBS3; HSPBP1; MOK; LOXL4; BCL2L12; UNC119; CDK10; CCBE1; CDK16; GDF5; FASTK; PDPK1; AURKA; RGS3; NRG1; GREM1; RAC2; TPST1; RAC1; HGS; REST; THOP1; MGAT1; AAK1; AP2A1; PIGU; TNC; GGT5; LMNA; ST6GALNAC4; DPH1; DPH2; ABI2; SHARPIN; WBP2; PPP3CC; MMP2; ATF3 | 5155 | 5143 |
| 0.000495365 | 0.009986582 | GO:0006886 | b | 4 | intracellular protein transport | 160; 162; 377; 972; 1263; 1384; 2064; 2621; 3429; 3434; 4000; 4130; 4641; 5058; 5191; 5371; 5878; 5880; 5905; 6192; 6447; 6449; 6774; 8086; 8907; 9146; 9230; 9454; 9531; 9632; 9697; 10043; 10113; 10938; 10945; 51114; 51172; 79971; 146691; 29984; 84885; 79711; 54662; 54732; 26088; 55357; 23511; 57214; 27183; 27352; 27429; 23381; 29927; 65083 | CD74; BAG3; AAAS; CEMIP; EHD1; ZDHHC12; TOM1; PLK3; AP1M1; MYO1C; NAGPA; HGS; IFIT1; SMG5; RPS4Y1; STAT3; ARF3; TBC1D2; TMED9; NUP188; RAB11B; HTRA2; PML; AP1B1; SGSM3; SCG5; ERBB2; MAP1A; NOL6; WLS; RAC2; RAB5C; RANGAP1; SGTA; GGA1; IFI27; AP2A1; VPS4A; SEC24C; LMNA; SEC61A1; HOMER3; RHOD; CRAT; PEX7; ZDHHC9; PREB; TRAM2; TOM1L2; TBC1D13; IPO4; KDELR1; PAK1; GAS6 | 1122 | 1120 |
| 0.00050055 | 0.009986582 | GO:0001944 | b | 4 | vasculature development | 290; 1464; 1490; 1545; 2022; 2064; 2852; 2969; 3162; 3164; 3340; 3397; 3726; 3953; 4313; 5176; 5371; 5598; 6050; 6464; 6774; 7070; 7128; 7763; 23213; 8751; 8877; 9146; 10014; 1717; 51421; 51548; 147372; 26585; 92140; 5170; 64855 | SPHK1; AMOTL2; HDAC5; JUNB; DHCR7; GPER1; ZFAND5; RNH1; MTDH; SIRT6; CYP1B1; NDST1; CCBE1; SULF1; MAPK7; FAM129B; PML; THY1; GTF2I; ERBB2; GREM1; SHC1; ENG; NR4A1; HGS; LEPR; SERPINF1; STAT3; CSPG4; HMOX1; TNFAIP3; ID1; CTGF; ANPEP; PDPK1; MMP2; ADAM15 | 684 | 684 |
| 0.000558226 | 0.010872118 | GO:0010810 | b | 4 | regulation of cell-substrate adhesion | 81; 1435; 2192; 3675; 5328; 5879; 5880; 6695; 7070; 7402; 8751; 133584; 29984; 23122; 91663; 26585 | EGFLAM; GREM1; RAC2; CLASP2; RAC1; ITGA3; CSF1; PLAU; UTRN; SPOCK1; ACTN4; FBLN1; RHOD; MYADM; THY1; ADAM15 | 206 | 206 |
| 0.00063767 | 0.012130563 | GO:0072358 | b | 4 | cardiovascular system development | 290; 1464; 1490; 1545; 2022; 2064; 2852; 2969; 3162; 3164; 3340; 3397; 3726; 3953; 4313; 5176; 5371; 5598; 6050; 6464; 6774; 7070; 7128; 7763; 23213; 8751; 8877; 9146; 10014; 1717; 51421; 51548; 147372; 26585; 92140; 5170; 64855 | SPHK1; AMOTL2; HDAC5; JUNB; DHCR7; GPER1; ZFAND5; RNH1; MTDH; SIRT6; CYP1B1; NDST1; CCBE1; SULF1; MAPK7; FAM129B; PML; THY1; GTF2I; ERBB2; GREM1; SHC1; ENG; NR4A1; HGS; LEPR; SERPINF1; STAT3; CSPG4; HMOX1; TNFAIP3; ID1; CTGF; ANPEP; PDPK1; MMP2; ADAM15 | 693 | 693 |
| 0.000723929 | 0.013458489 | GO:0034976 | b | 4 | response to endoplasmic reticulum stress | 37; 65992; 467; 595; 598; 1388; 2134; 2907; 4000; 5371; 6449; 6464; 10113; 55738; 79876; 83667; 51608; 91304; 30827 | BCL2L1; TMEM259; ACADVL; SHC1; SESN2; CCND1; EXTL1; GRINA; ARFGAP1; SGTA; CXXC1; UBA5; DDRGK1; PML; ATF6B; PREB; GET4; LMNA; ATF3 | 276 | 274 |
| 0.000860258 | 0.014808401 | GO:0006986 | b | 4 | response to unfolded protein | 37; 467; 595; 1388; 2134; 3297; 3338; 4000; 6464; 9531; 10113; 10808; 55738; 30827 | HSF1; BAG3; ACADVL; SHC1; CCND1; EXTL1; PREB; DNAJC4; CXXC1; ARFGAP1; ATF6B; LMNA; ATF3; HSPH1 | 176 | 174 |
| 0.000862478 | 0.014808401 | GO:0002444 | b | 4 | myeloid leukocyte mediated immunity | 29; 221; 290; 823; 976; 1265; 1535; 3162; 3614; 5328; 5709; 5834; 5878; 5879; 5880; 6793; 8635; 8804; 8907; 23406; 9961; 10043; 10312; 10383; 10493; 79792; 23586; 57153; 124583; 29952; 5170 | CREG1; ADGRE5; TOM1; STK10; AP1M1; SLC44A2; PSMD3; RNASET2; DDX58; GSDMD; IMPDH1; COTL1; PYGB; CAPN1; DPP7; MVP; ALDH3B1; RAC2; RAB5C; RAC1; PLAU; ABR; CYBA; VAT1; CNN2; TUBB4B; HMOX1; TCIRG1; ANPEP; CANT1; PDPK1 | 558 | 556 |
| 0.000867553 | 0.014808401 | GO:0006796 | b | 4 | phosphate-containing compound metabolic process | 27; 60; 271; 377; 65992; 467; 471; 476; 595; 896; 972; 1263; 1435; 1464; 1490; 1497; 196883; 1737; 1760; 1848; 2022; 2064; 2068; 2192; 2252; 2621; 2852; 3084; 3297; 3340; 3397; 3481; 3572; 3614; 3628; 3636; 4055; 4512; 4522; 4540; 4665; 4833; 4967; 5058; 5127; 5167; 5371; 5531; 5533; 5536; 27315; 5598; 5599; 5606; 5833; 5879; 5880; 5987; 5998; 55627; 6199; 6241; 6300; 6464; 6574; 6576; 6774; 6793; 7070; 7083; 7128; 7538; 7867; 22924; 6790; 8200; 8226; 8460; 8482; 8558; 8611; 8613; 8733; 8871; 8877; 9094; 9133; 9146; 9500; 9961; 10152; 10174; 10899; 10908; 10922; 10985; 51477; 221927; 5891; 400916; 79143; 84231; 51548; 84335; 84836; 83667; 128869; 150763; 80221; 347735; 54386; 55163; 22848; 22993; 23659; 23770; 57153; 57214; 91663; 26289; 26585; 23381; 26090; 5170; 5298; 64748 | HSF1; MT-CO1; PGAP2; PLK3; GPAT2; IL6ST; GPER1; FBLN1; FKBP8; SYNJ2; LTBR; OGDH; MT-ND5; NDST1; PPP4C; DMPK; THY1; STK10; MAPRE3; ERBB2; ADCY4; SHC1; SLC25A1; TRIM27; ENPP1; TERF2IP; SEMA7A; BRAT1; NME4; ATIC; CSPG4; CCND1; MAGED1; CCND3; CSF1; PNPO; ACTB; PAK1; SERINC2; GREM1; TRAF7; MYADM; MAPKAPK3; TPST1; CTGF; ABHD12; AKT1S1; PNPLA6; IGF2; SMG5; SIRT6; PLPPR2; ENG; ACSF2; DLAT; TK1; FGF7; JTB; PLA2G15; GAS6; MTHFD1; PPP5C; TNFAIP3; MOK; DDRGK1; NAB2; CD74; PI4KB; SESN2; AMPD2; SMPD4; CEMIP; MBOAT7; MAPK12; CHCHD10; ABL2; STAT3; ARF3; RPS6KB2; MAPK7; PML; MAPK8; DUSP6; PLPP1; PLPP3; GCN1; SLC20A1; PUDP; AK5; ID1; ZFP36; GPAA1; ATP1A1; MVP; SPHK1; HMGXB3; ERCC2; MAP2K3; RRM2; SORBS3; INPPL1; UNC119; CDK10; CDK16; IMPDH1; GDF5; FASTK; AURKA; RGS3; NRG1; SLC44A2; PCYT2; RAC2; ISYNA1; RAC1; HGS; ABHD14B; AAK1; PIGU; CTNS; CCNB2; INPP1; ABI2; PDPK1; PPP3CC; ATF3 | 3208 | 3201 |
| 0.000868953 | 0.014808401 | GO:0044089 | b | 4 | positive regulation of cellular component biogenesis | 822; 1490; 2068; 3297; 3397; 4641; 4926; 5058; 5879; 5880; 5987; 6624; 7070; 7162; 9076; 9146; 7013; 10152; 10174; 10435; 10458; 10801; 11135; 79647; 51548; 116985; 23122; 57175; 57606; 27183 | HSF1; NUMA1; ERCC2; MYO1C; CORO1B; TPBG; SEPT9; SORBS3; CLASP2; ARAP1; CLDN1; THY1; RAC2; SIRT6; RAC1; CDC42EP2; CDC42EP1; TRIM27; HGS; VPS4A; CAPG; FSCN1; AKIRIN1; SLAIN2; ABI2; ID1; CTGF; TERF1; BAIAP2; PAK1 | 533 | 532 |
| 0.000940088 | 0.015693712 | GO:0043412 | b | 4 | macromolecule modification | 27; 60; 322; 331; 65992; 467; 595; 896; 972; 1263; 1435; 1464; 1490; 1760; 1801; 1802; 1848; 2022; 2064; 2068; 2134; 2192; 2252; 2590; 2621; 2852; 3084; 3297; 3340; 3371; 3397; 3429; 3481; 3572; 4000; 4055; 4085; 4245; 4665; 4967; 5058; 5127; 5167; 5179; 5327; 5371; 5531; 5533; 5536; 27315; 5598; 5599; 5606; 5696; 5709; 5879; 5880; 5978; 5987; 5998; 6199; 6300; 6464; 6484; 6774; 6793; 7052; 7064; 7070; 7128; 7538; 7867; 22924; 6790; 7991; 8078; 8200; 8266; 8460; 8482; 8507; 8558; 8611; 8613; 8733; 8877; 9094; 9133; 9146; 9230; 140459; 9500; 9810; 9961; 10014; 10152; 10174; 10432; 10489; 10612; 10678; 10899; 10922; 11065; 11070; 10985; 11176; 11285; 11313; 51114; 51172; 143888; 221927; 5891; 79102; 84231; 112398; 79876; 79903; 51548; 84335; 133584; 81858; 83667; 51200; 51246; 84171; 84287; 84885; 128869; 348180; 54386; 54461; 54587; 54681; 55214; 55343; 113000; 22848; 23558; 23586; 23640; 23770; 57214; 253980; 91663; 26133; 26155; 26262; 26585; 27090; 60509; 60681; 126789; 29089; 26090; 30827; 5170; 63891; 64840; 64855; 65094 | HSF1; TGM2; PGAP2; FKBP10; TUSC3; PLK3; IL6ST; GPER1; ZDHHC16; ABHD12; NAA60; FBLN1; FKBP8; LTBR; CTU2; OGDH; ASB6; B3GNT2; NDST1; PPP4C; ZDHHC12; APBB1; AKT1S1; P3H2; DMPK; THY1; IFI27; MAPRE3; ERBB2; SHC1; EXTL1; TRIM27; ENPP1; TERF2IP; TSPAN17; SEMA7A; BRAT1; KCTD13; LRRC41; CSPG4; CCND1; MAGED1; CCND3; CSF1; ENC1; ACTB; PAK1; EGFLAM; KDELC2; CXXC1; TRAF7; MYADM; BAZ2A; MAPKAPK3; TMEM115; DDX58; TPST1; CTGF; CPA4; RAB11B; FAM129B; B4GALT7; IGF2; SIRT6; ENG; RNF40; P4HTM; UBA5; RPUSD1; FGF7; PLAT; GALNT2; JTB; EGLN2; LYPLA2; GAS6; ZDHHC9; PPP5C; UBE2C; TNFAIP3; MOK; SHISA5; STK10; DDRGK1; TRIM3; RNF123; UBE2T; NAB2; CD74; AGBL5; HDAC5; SESN2; UBL4A; NAGPA; XIAP; CEMIP; MAPK12; ABL2; STAT3; JMJD4; NOC2L; RPS6KB2; MAPK7; RBM14; PML; MAPK8; DUSP6; PENK; FBXW5; SLC35C1; PLPP1; PLPP3; CCNB2; GCN1; MXRA8; RNF26; PORCN; ID1; ZFP36; GPAA1; ST3GAL4; PSMB8; TRPC4AP; MVP; SPHK1; MAD2L1; ERCC2; USP5; PSMD3; MAP2K3; SORBS3; PUSL1; HSPBP1; LOXL4; UNC119; CDK10; CDK16; GDF5; FASTK; AURKA; RGS3; NRG1; GREM1; RAC2; RAC1; HGS; REST; THOP1; MGAT1; AAK1; PIGU; TNC; PDPK1; LMNA; ST6GALNAC4; DPH1; DPH2; ABI2; SHARPIN; WBP2; PPP3CC; ATF3 | 4303 | 4295 |
| 0.000992187 | 0.016225924 | GO:0045785 | b | 4 | positive regulation of cell adhesion | 972; 1435; 2064; 3481; 3572; 3575; 3675; 4651; 5058; 5420; 5879; 7030; 7052; 7070; 7402; 8613; 9265; 10370; 10808; 133584; 29984; 80381; 91663; 5170 | CD74; IL7R; TGM2; ITGA3; EGFLAM; IL6ST; PODXL; MYADM; IGF2; HSPH1; TFE3; CITED2; THY1; ERBB2; PLPP3; RAC1; CYTH3; RHOD; CD276; CSF1; UTRN; PDPK1; PAK1; MYO10 | 395 | 394 |
| 0.001023061 | 0.016225924 | GO:0051726 | b | 4 | regulation of cell cycle | 60; 322; 65992; 595; 598; 896; 1263; 1490; 2068; 2621; 2852; 3164; 3297; 3397; 3398; 3481; 3726; 4085; 4926; 5127; 5371; 6241; 6300; 6774; 6878; 7128; 22924; 6790; 8558; 8877; 9055; 9133; 7013; 9810; 10370; 10383; 10432; 10514; 11065; 11180; 5891; 57060; 146691; 90313; 83746; 54461; 54962; 90480; 26073; 27183; 27352; 27429; 60672 | BCL2L1; HSF1; SPHK1; MAD2L1; NUMA1; ERCC2; JUNB; GPER1; RRM2; MAPK12; PRC1; MIIP; HTRA2; STAT3; TIPIN; POLDIP2; CDK10; CDK16; APBB1; PCBP4; L3MBTL2; AURKA; RBM14; PML; CITED2; PLK3; MAPRE3; MYBBP1A; IGF2; WDR6; FBXW5; CCNB2; NR4A1; RNF40; SGSM3; VPS4A; TAF6; GADD45GIP1; CCND1; TUBB4B; CCND3; ID2; TNFAIP3; ID1; MOK; CTGF; TERF1; TOM1L2; DDRGK1; TP53I13; ACTB; UBE2C; GAS6 | 1139 | 1129 |
| 0.001031477 | 0.016225924 | GO:0019221 | b | 4 | cytokine-mediated signaling pathway | 81; 595; 598; 972; 1265; 1435; 2621; 3162; 3429; 3434; 3437; 3455; 3572; 3575; 3590; 3636; 3726; 3856; 3875; 3953; 4055; 4313; 4502; 5055; 5371; 5696; 6464; 6624; 6774; 6778; 7128; 8519; 8877; 10437; 10581; 81858; 56928; 29965 | BCL2L1; CD74; SPHK1; MT2A; IFI27; JUNB; IL6ST; IFNAR2; KRT8; IFIT3; IFIT1; LTBR; STAT6; STAT3; INPPL1; PML; IFITM2; IFITM1; SHC1; IL7R; IFI30; LEPR; SERPINB2; FSCN1; SPPL2B; CNN2; CCND1; ACTN4; HMOX1; CSF1; TNFAIP3; PSMB8; IL11RA; CDIP1; GAS6; MMP2; SHARPIN; KRT18 | 738 | 737 |
| 0.001095368 | 0.016487316 | GO:0046903 | b | 4 | secretion | 29; 81; 221; 290; 322; 595; 823; 972; 976; 1265; 1490; 1535; 2022; 2252; 2621; 2852; 3084; 3162; 3481; 3614; 3671; 5058; 5127; 5270; 5328; 5371; 5413; 5709; 5834; 5878; 5879; 5880; 5978; 5987; 6447; 6793; 7128; 7200; 7421; 8425; 8635; 8804; 8907; 9146; 9230; 23406; 9961; 10043; 10113; 10312; 10383; 10493; 10652; 10758; 11188; 51172; 79792; 79971; 84283; 51150; 23265; 152579; 23122; 23586; 57153; 124583; 27183; 29952; 5170; 64855; 63910 | CD74; MVP; NAGPA; VDR; CREG1; ADGRE5; TOM1; SCG5; AP1M1; SLC44A2; PSMD3; IMPDH1; GPER1; NISCH; SCFD2; SEPT5; RNASET2; YKT6; SERPINE2; WLS; DDX58; SDF4; RAC1; CDK16; APBB1; COTL1; TMEM79; PYGB; RAB11B; CAPN1; FAM129B; NRG1; TRAF3IP2; STK10; DPP7; IGF2; TRH; ALDH3B1; LTBP4; RAC2; CLASP2; RAB5C; ENG; TRIM27; HGS; PLAU; GSDMD; PML; ABR; CYBA; REST; FGF7; SLC17A9; VAT1; EXOC7; VPS4A; ACTN4; CCND1; TUBB4B; ISLR; CNN2; HMOX1; TNFAIP3; PREB; TCIRG1; CTGF; ANPEP; CANT1; PDPK1; PAK1; GAS6 | 1630 | 1626 |
| 0.001098821 | 0.016487316 | GO:0007249 | b | 4 | I-kappaB kinase/NF-kappaB signaling | 65992; 972; 3162; 4055; 5536; 6275; 6574; 7052; 7128; 10758; 79971; 81858; 51246; 54386; 57153; 124583; 92140; 5170 | CD74; LTBR; TGM2; SLC20A1; CANT1; PPP5C; WLS; HMOX1; SLC44A2; TNFAIP3; SHISA5; PDPK1; TERF2IP; TRAF3IP2; DDRGK1; S100A4; MTDH; SHARPIN | 262 | 262 |
| 0.00110856 | 0.016487316 | GO:0001501 | b | 4 | skeletal system development | 309; 65992; 1291; 1490; 2022; 2134; 3340; 3481; 3636; 4209; 4313; 4522; 4665; 5191; 5916; 6424; 7059; 7373; 7421; 7763; 8200; 23213; 338773; 56172; 29985; 124565; 26585; 53834; 64651 | FGFRL1; VDR; ANKH; CSRNP1; MTHFD1; ZFAND5; SULF1; INPPL1; SLC38A10; NDST1; ANXA6; THBS3; GDF5; PEX7; IGF2; GREM1; ENG; EXTL1; DDRGK1; SLC39A3; RARG; SFRP4; TMEM119; COL14A1; CTGF; MEF2D; COL6A1; MMP2; NAB2 | 518 | 516 |
| 0.001128862 | 0.016489452 | GO:0007160 | b | 4 | cell-matrix adhesion | 1435; 1490; 3675; 5328; 22801; 5879; 7059; 7070; 7402; 7791; 8751; 29984; 54749; 23122; 26585; 5170 | UTRN; CLASP2; RAC1; GREM1; ITGA3; CSF1; PLAU; THBS3; CTGF; ITGA11; ADAM15; PDPK1; THY1; EPDR1; ZYX; RHOD | 220 | 220 |
| 0.001200678 | 0.017230786 | GO:0043434 | b | 4 | response to peptide hormone | 445; 896; 1490; 1535; 196883; 2852; 3164; 3297; 3481; 3636; 4641; 5058; 5167; 5905; 6464; 6774; 9810; 10014; 10312; 10458; 29988; 83667; 22937; 155066; 5170 | HSF1; HDAC5; SESN2; MYO1C; GPER1; SCAP; ASS1; STAT3; INPPL1; IGF2; SHC1; NR4A1; RANGAP1; ENPP1; CYBA; PDPK1; RNF40; ADCY4; SLC2A8; CCND3; TCIRG1; CTGF; BAIAP2; ATP6V0E2; PAK1 | 425 | 423 |
| 0.001255658 | 0.017709111 | GO:1901652 | b | 4 | response to peptide | 445; 896; 1490; 1535; 196883; 2852; 3164; 3297; 3397; 3481; 3636; 4641; 5058; 5167; 5905; 6464; 6774; 7128; 9810; 10014; 10312; 10458; 29988; 83667; 22937; 23641; 155066; 5170 | HSF1; HDAC5; SESN2; MYO1C; GPER1; SCAP; ASS1; LDOC1; STAT3; INPPL1; IGF2; SHC1; NR4A1; RANGAP1; ENPP1; CYBA; PDPK1; RNF40; ADCY4; SLC2A8; CCND3; TNFAIP3; ID1; TCIRG1; CTGF; BAIAP2; ATP6V0E2; PAK1 | 498 | 496 |
| 0.001289788 | 0.017882152 | GO:0071702 | b | 4 | organic substance transport | 60; 81; 160; 162; 322; 377; 972; 1263; 1384; 1497; 1535; 2064; 2621; 2852; 3297; 3429; 3434; 3875; 4000; 4130; 4641; 4833; 5058; 5127; 5167; 5191; 5371; 27315; 5878; 5880; 5905; 5978; 5987; 6192; 6447; 6449; 6524; 6563; 6576; 6774; 7128; 7200; 7263; 7538; 8086; 8140; 8425; 8907; 9094; 9146; 9230; 9399; 9454; 9531; 9632; 9697; 9961; 10043; 10113; 10312; 10612; 10652; 10758; 10938; 10945; 10948; 10999; 11070; 11188; 51114; 51172; 55738; 10280; 79792; 51548; 79971; 113178; 81031; 146691; 124565; 29984; 29988; 93343; 83667; 83985; 84885; 79711; 23265; 152579; 54662; 54732; 55144; 26088; 55343; 55357; 23511; 23586; 155066; 57153; 57214; 26020; 26119; 27183; 27352; 27429; 23381; 126129; 29927; 65083 | HSF1; AAAS; PGAP2; ZDHHC12; PLK3; WLS; GPER1; SCFD2; SMG5; NOL6; SESN2; GSDMD; APBB1; NISCH; ERBB2; SCAMP4; AP1M1; RAB5C; SLC25A1; TRIM27; RANGAP1; ENPP1; IFI27; SEC24C; HOMER3; NME4; SLC2A8; STARD3; ACTB; KDELR1; PAK1; TRH; SLC7A5; CPT1C; ARFGAP1; TST; TMEM115; IFIT1; DDX58; SCG5; RAB11B; TRAF3IP2; MAP1A; SIRT6; SLC14A1; SGTA; RHOD; PEX7; ZDHHC9; TNFAIP3; LDLRAP1; TCIRG1; SGSM3; TRIM3; GAS6; CD74; BAG3; EHD1; MYO1C; SLC2A10; HTRA2; RPS4Y1; STAT3; ARF3; TBC1D2; TMED9; PML; SLC35C1; MVP; LRRC8D; YKT6; GGA1; VPS4A; SLC27A4; MVB12A; STOML1; PREB; ZFP36; TRAM2; TBC1D13; SIGMAR1; NAGPA; CEMIP; TOM1; SPNS1; SLC5A2; UNC119; CDK16; NUP188; SLC38A10; AP1B1; LRP10; SLC44A2; LTBP4; RAC2; HGS; REST; CYBA; AP2A1; LMNA; CTNS; IPO4; CRAT; EXOC7; ACTN4; TOM1L2; ATP6V0E2; KRT18; SEC61A1 | 2735 | 2730 |
| 0.001342154 | 0.018298028 | GO:0002275 | b | 4 | myeloid cell activation involved in immune response | 29; 221; 290; 823; 976; 1265; 1535; 3162; 3614; 5328; 5709; 5834; 5878; 5879; 5880; 6793; 8635; 8804; 8907; 23406; 9961; 10043; 10312; 10383; 10493; 79792; 57153; 124583; 29952; 5170 | CREG1; ADGRE5; TOM1; STK10; AP1M1; SLC44A2; PSMD3; RNASET2; GSDMD; IMPDH1; COTL1; PYGB; CAPN1; DPP7; MVP; ALDH3B1; RAC2; RAB5C; RAC1; PLAU; ABR; CYBA; VAT1; CNN2; TUBB4B; HMOX1; TCIRG1; ANPEP; CANT1; PDPK1 | 549 | 547 |
| 0.0014993 | 0.020105364 | GO:0006643 | b | 4 | membrane lipid metabolic process | 221; 1545; 2531; 27315; 55627; 6484; 8611; 8613; 8733; 8877; 128869; 347735; 23659; 27090; 27429 | PLPP1; PLA2G15; ALDH3B1; PGAP2; CYP1B1; ST3GAL4; KDSR; SPHK1; SERINC2; SMPD4; GPAA1; ST6GALNAC4; PLPP3; HTRA2; PIGU | 207 | 205 |
| 0.001538866 | 0.020303103 | GO:1901137 | b | 4 | carbohydrate derivative biosynthetic process | 160; 271; 471; 1464; 196883; 1737; 2134; 2590; 2817; 3340; 3614; 4245; 4833; 4967; 27315; 6241; 6484; 6576; 6774; 7083; 7991; 8226; 8733; 10678; 11070; 11285; 51172; 143888; 51548; 128869; 55343; 57214; 124583; 26289; 27090; 29925; 22822 | NAGPA; PGAP2; TUSC3; AMPD2; KDELC2; CEMIP; RRM2; PHLDA1; TMEM115; OGDH; STAT3; B3GNT2; NDST1; IMPDH1; B4GALT7; SLC35C1; NME4; SIRT6; ST3GAL4; EXTL1; SLC25A1; GMPPB; MGAT1; DLAT; AP2A1; PUDP; TK1; ST6GALNAC4; GALNT2; ADCY4; ATIC; CSPG4; GPC1; AK5; GPAA1; CANT1; PIGU | 729 | 728 |
| 0.001581307 | 0.02053189 | GO:0010941 | b | 4 | regulation of cell death | 29; 81; 220; 322; 331; 396; 65992; 467; 598; 972; 1263; 1490; 1545; 1848; 2002; 2022; 2621; 2852; 2907; 3162; 3297; 3397; 3437; 3572; 3575; 3776; 3875; 4000; 4055; 4085; 4192; 4550; 5055; 5176; 5371; 5598; 5599; 5916; 5978; 6424; 6464; 6774; 7052; 7128; 7421; 7538; 6790; 8200; 8877; 9500; 9531; 9828; 10370; 10514; 400916; 79156; 10280; 84231; 112398; 84335; 57124; 83596; 23770; 91304; 26155; 26585; 92140; 27429; 5170; 64855; 64857 | BCL2L1; CD74; TGM2; MAD2L1; NOC2L; TMEM259; BAG3; PLK3; SPHK1; FKBP8; IL6ST; GPER1; XIAP; VDR; GDF5; PLEKHF1; CHCHD10; MTDH; IFIT3; HTRA2; ALDH1A3; LTBR; MDK; SERPINB2; CYP1B1; SFRP4; RARG; BCL2L12; TRAF7; CD248; ARHGEF17; APBB1; KCNK2; AKT1S1; MAPK7; ARHGDIA; PML; CITED2; MAPK8; DUSP6; ZFP36; AURKA; GREM1; SHC1; ENG; GRINA; MYBBP1A; REST; ABR; SERPINF1; STAT3; LMNA; MT-RNR2; PLEKHG2; EGLN2; IL7R; HSF1; ACTN4; FAM129B; MAGED1; HMOX1; TNFAIP3; ID1; CTGF; ELK1; DDRGK1; PDPK1; KRT18; SIGMAR1; ATF3; GAS6 | 1651 | 1649 |
| 0.001622156 | 0.020733186 | GO:0009893 | b | 4 | positive regulation of metabolic process | 27; 60; 81; 322; 331; 445; 65992; 467; 595; 896; 972; 1263; 1388; 1435; 1464; 1490; 1535; 1848; 1889; 2002; 2022; 2064; 2068; 2192; 2252; 2621; 2852; 3084; 3159; 3162; 3164; 3297; 3340; 3371; 3397; 3398; 3481; 3572; 3575; 3675; 3726; 3953; 4000; 4055; 4192; 4209; 4641; 4665; 4782; 5058; 5118; 5371; 5598; 5599; 5606; 5879; 5916; 5978; 6199; 6300; 55859; 6424; 6449; 6464; 6601; 6774; 6778; 6793; 7030; 7041; 7128; 7421; 7538; 7867; 22924; 6790; 8078; 8200; 8482; 8558; 8877; 9094; 9146; 9500; 9531; 9612; 9810; 10014; 10025; 10174; 10370; 10432; 10514; 10808; 10899; 11065; 10985; 192669; 51157; 221927; 400916; 79156; 84231; 112398; 51548; 112770; 147372; 84836; 83596; 83667; 51334; 116985; 54386; 26088; 22937; 23274; 80381; 23558; 285704; 23586; 23640; 23764; 57214; 253980; 91304; 26119; 26585; 92140; 27352; 27429; 30827; 5170; 64651; 64855; 65094 | HSF1; SMARCC2; PLK3; IL6ST; GPER1; FBLN1; ASS1; MAFF; LTBR; RARG; NDST1; APBB1; STK10; MAPRE3; ERBB2; SHC1; TERF2IP; SEMA7A; BRAT1; KCTD13; CSPG4; CCND1; MAGED1; CCND3; CSF1; ELK1; PRR16; CXXC1; ACTB; PAK1; IL7R; STAT6; VDR; ITGA3; MTDH; MAPKAPK3; DDX58; HSPH1; TRAF7; CTGF; TFE3; FAM129B; PCOLCE; TGFB1I1; MYBBP1A; IGF2; SIRT6; GLMP; ENG; SGTA; ZNF580; FGF7; RNF40; JTB; EGLN2; CSRNP1; UBE2C; CD276; HMOX1; TNFAIP3; MEF2D; AGO3; NAB2; CD74; BAG3; HDAC5; SESN2; MYO1C; DDRGK1; XIAP; MAPK12; CHCHD10; ABL2; RGMB; NCOR2; HTRA2; MDK; STAT3; JMJD4; RPS6KB2; MAPK7; RBM14; PML; CITED2; MAPK8; DUSP6; CLEC16A; CCBE1; HMGA1; GGA1; GCN1; SFRP4; ID2; ID1; ZFP36; LDLRAP1; SPHK1; CEMIP; TMEM259; ERCC2; JUNB; USP5; MAP2K3; ECE1; SCAP; SORBS3; HSPBP1; PLEKHF1; ARAP1; BCL2L12; UNC119; CDK10; GDF5; AURKA; MED16; NRG1; GREM1; BEX1; RAC1; NR4A1; HGS; REST; ABHD14B; CYBA; SGSM3; TNC; PDPK1; LMNA; LEPR; NFIC; ACTN4; WBP2; ATF6B; GAS6; ATF3 | 3537 | 3525 |
| 0.001716351 | 0.021599622 | GO:0031032 | b | 4 | actomyosin structure organization | 1265; 1490; 2006; 3856; 3880; 5058; 5879; 7791; 10174; 116985; 23122; 253980; 27183; 55616 | KCTD13; ELN; CLASP2; ARAP1; CNN2; RAC1; CTGF; ASAP3; ZYX; SORBS3; KRT8; PAK1; KRT19; VPS4A | 187 | 187 |
| 0.00175087 | 0.02163912 | GO:0030163 | b | 4 | protein catabolic process | 160; 65992; 1263; 2817; 3084; 3429; 4085; 4130; 5270; 5371; 5696; 5709; 6448; 6449; 7041; 7128; 6790; 8078; 8507; 8722; 9146; 9531; 9810; 10312; 10612; 11065; 11313; 112398; 81858; 93343; 51608; 54442; 54461; 26088; 23274; 23640; 253980; 91304; 26133; 27183; 27352; 27429; 26090; 63891 | BAG3; MAD2L1; KCTD5; PLK3; USP5; PSMD3; DDRGK1; HTRA2; ABHD12; SERPINE2; MVB12A; TMEM259; CLEC16A; AURKA; PML; TGFB1I1; IFI27; MAP1A; HSPBP1; NRG1; SGSH; HGS; CTSF; SGTA; GGA1; GPC1; AP2A1; VPS4A; RNF40; GET4; KCTD13; EGLN2; LYPLA2; UBE2C; TNFAIP3; FBXW5; TCIRG1; ENC1; PSMB8; SGSM3; TRIM3; RNF123; SHARPIN; TRPC4AP | 918 | 916 |
| 0.001805151 | 0.02163912 | GO:0040017 | b | 4 | positive regulation of locomotion | 81; 65992; 972; 1435; 2252; 2621; 2852; 3162; 3675; 4641; 5058; 5328; 5420; 5606; 5879; 5880; 6774; 7070; 8482; 8613; 8877; 51157; 79647; 29984; 23122; 57214; 91663; 27183; 5170 | CD74; SPHK1; CEMIP; ITGA3; MYO1C; PODXL; MYADM; STAT3; THY1; PLPP3; RAC2; CLASP2; RAC1; PLAU; ZNF580; MAP2K3; VPS4A; FGF7; SEMA7A; RHOD; GPER1; AKIRIN1; ACTN4; HMOX1; CSF1; DDRGK1; PDPK1; PAK1; GAS6 | 533 | 533 |
| 0.001813458 | 0.02163912 | GO:0060348 | b | 4 | bone development | 309; 1291; 2022; 3636; 4209; 4665; 5191; 5916; 6424; 7059; 7373; 23213; 338773; 124565; 26585 | SLC38A10; GREM1; SFRP4; PEX7; INPPL1; ENG; ANXA6; COL14A1; THBS3; SULF1; TMEM119; RARG; MEF2D; COL6A1; NAB2 | 210 | 209 |
| 0.001825305 | 0.02163912 | GO:0033993 | b | 4 | response to lipid | 27; 29; 445; 65992; 476; 595; 1490; 1535; 2002; 2022; 2852; 3164; 3297; 3371; 3726; 4192; 5058; 5176; 5179; 5536; 5599; 5916; 5978; 6774; 7041; 7128; 7200; 7421; 7538; 7867; 8611; 8751; 9076; 9536; 9612; 10014; 10025; 10432; 112398; 79876; 23558; 23641; 92140; 27429 | HSF1; VDR; HDAC5; JUNB; GPER1; ADAM15; ABL2; ASS1; NCOR2; LDOC1; MDK; MTDH; STAT3; RARG; MAPKAPK3; ZFP36; HTRA2; MED16; RBM14; TGFB1I1; MAPK8; PENK; PLPP1; TRH; ENG; UBA5; NR4A1; REST; ABR; CYBA; SERPINF1; TNC; CLDN1; PTGES; EGLN2; CCND1; PPP5C; TNFAIP3; CTGF; ELK1; ATP1A1; DDRGK1; WBP2; PAK1 | 919 | 918 |
| 0.001934212 | 0.022602652 | GO:0008610 | b | 4 | lipid biosynthetic process | 37; 220; 221; 377; 476; 972; 2252; 2531; 2852; 3636; 4047; 5191; 27315; 5833; 5978; 55627; 6484; 7421; 8611; 8613; 8733; 8871; 8877; 9536; 60343; 1717; 10948; 51477; 79143; 80270; 128869; 150763; 22937; 57153; 27349; 5298 | CD74; SPHK1; ACADVL; PI4KB; FAM3A; PLPP3; DHCR7; GPER1; SMPD4; PGAP2; MBOAT7; SCAP; ALDH1A3; INPPL1; ARF3; SLC44A2; LSS; SYNJ2; PLPP1; ALDH3B1; ISYNA1; ST3GAL4; PCYT2; REST; GPAT2; VDR; PIGU; MCAT; FGF7; PTGES; KDSR; PEX7; HSD3B7; GPAA1; ATP1A1; STARD3 | 713 | 712 |
| 0.002025032 | 0.022992946 | GO:0043312 | b | 4 | neutrophil degranulation | 29; 221; 290; 823; 976; 1265; 1535; 3614; 5328; 5709; 5834; 5878; 5879; 6793; 8635; 8804; 8907; 23406; 9961; 10043; 10312; 10383; 10493; 79792; 57153; 124583; 29952 | CREG1; ADGRE5; TOM1; STK10; AP1M1; SLC44A2; PSMD3; RNASET2; GSDMD; IMPDH1; COTL1; PYGB; CAPN1; DPP7; MVP; ALDH3B1; RAB5C; RAC1; PLAU; ABR; CYBA; VAT1; CNN2; TUBB4B; TCIRG1; ANPEP; CANT1 | 490 | 488 |
| 0.002080719 | 0.022992946 | GO:0007399 | b | 4 | nervous system development | 27; 29; 60; 10939; 160; 220; 322; 396; 471; 1435; 1464; 1497; 1627; 2022; 2064; 2068; 2621; 2817; 2852; 3084; 3340; 3371; 3397; 3398; 3572; 3675; 4130; 4192; 4209; 4512; 4522; 4665; 4681; 4741; 4967; 5058; 5127; 5176; 5179; 5191; 5270; 5533; 5598; 5599; 5879; 5880; 5916; 5978; 55859; 6455; 6464; 6601; 6695; 6774; 7070; 7162; 23154; 6790; 8140; 8200; 23213; 8482; 8507; 8871; 8877; 9076; 9094; 9531; 10014; 10152; 10381; 10458; 10612; 10678; 10938; 11313; 79143; 10280; 79971; 81858; 115908; 50863; 51150; 84287; 84934; 118813; 54587; 55201; 22907; 55715; 23770; 25987; 27429; 64855 | SMARCC2; MT-CO1; ZDHHC16; LYPLA2; WLS; IL6ST; GPER1; TPBG; AFG3L2; GDF5; DBN1; FKBP8; OGDH; SDF4; RARG; NDST1; APBB1; THY1; ERBB2; SHC1; ARHGDIA; SEMA7A; ATIC; CSPG4; CSF1; ENC1; ACTB; PAK1; ITGA3; DHX30; SERPINE2; NBL1; FAM129B; CLDN1; MAP1A; ENG; TUBB3; MAP1S; GAS6; B3GNT2; PEX7; CTHRC1; SPOCK1; MEF2D; BAIAP2; TRIM3; NAB2; BAG3; HDAC5; EHD1; MBOAT7; ABL2; SH3GL1; HTRA2; MDK; STAT3; MAPK7; MAPK8; PENK; SLC7A5; SERPINF1; MXRA8; RITA1; ZFYVE27; MTHFD1; ID2; ID1; NTM; SIGMAR1; SPHK1; ERCC2; TSKU; ALDH1A3; NEFM; UNC119; CDK16; SULF1; AURKA; DOK4; SYNJ2; NRG1; RAC2; BEX1; RAC1; REST; GPC1; ABR; AP2A1; TNC; CTNS; ABI2; NCDN; PPP3CC; SHARPIN | 2335 | 2328 |
| 0.002111435 | 0.022992946 | GO:0048523 | b | 4 | negative regulation of cellular process | 29; 37; 81; 160; 309; 322; 331; 396; 445; 65992; 467; 476; 595; 598; 822; 896; 972; 1263; 1388; 1490; 1497; 1535; 1545; 1848; 2022; 2064; 2192; 2621; 2817; 2852; 2907; 3084; 3159; 3162; 3164; 3297; 3371; 3397; 3398; 3429; 3437; 3481; 3489; 3572; 3575; 3636; 3675; 3726; 3776; 3875; 3953; 4000; 4085; 4130; 4192; 4320; 4550; 4665; 4681; 4782; 5055; 5058; 5167; 5176; 5270; 5327; 5371; 5420; 5598; 5599; 5879; 5916; 5978; 5987; 5998; 6241; 6300; 6424; 6449; 6464; 6601; 6695; 6774; 6778; 6878; 7041; 7070; 7128; 7200; 7421; 7538; 6790; 8200; 23213; 8482; 8507; 8519; 8558; 8611; 8613; 8751; 8804; 8877; 9094; 9146; 7013; 9454; 9500; 9531; 9536; 9537; 9612; 9810; 60343; 9961; 10014; 10174; 10370; 10432; 10493; 10514; 11070; 11176; 11180; 11188; 11285; 192669; 79647; 79759; 51548; 84335; 375033; 57060; 146691; 114990; 90313; 115908; 83596; 83667; 83746; 116985; 84232; 84814; 84934; 54386; 54962; 55214; 55343; 23122; 23274; 23641; 23770; 57175; 57214; 253980; 90480; 91304; 25987; 26073; 91663; 26155; 26585; 92140; 27183; 27352; 27429; 60672; 53834; 30851; 5170; 64855 | BCL2L1; HSF1; FGFRL1; ACADVL; FAM3A; PLK3; FAM129B; IL6ST; GPER1; NISCH; GDF5; FBLN1; ASS1; LDOC1; RARG; APBB1; ZNF668; P3H2; THY1; IFI27; ERBB2; WDR6; IFITM1; SHC1; GRINA; ARHGDIA; ENPP1; TERF2IP; SERPINB2; SEMA7A; KCTD13; PODXL; VAT1; AKIRIN1; CCND1; MAGED1; CCND3; GADD45GIP1; TERF1; ENC1; MAF1; PAK1; RNF40; L3MBTL2; IL7R; VDR; MMP11; TRH; ITGA3; GREM1; MYADM; BAZ2A; MTDH; IFIT3; TMEM115; SERPINE2; NBL1; ANXA6; ZFP36; NCOR2; PCBP4; AKT1S1; SULF1; TGFB1I1; B4GALT7; MYBBP1A; IGF2; MAP1A; SIRT6; ENG; SGTA; IGFBP6; STAT3; HGS; CAPG; TAF6; GAS6; TP53I11; TP53I13; HMOX1; TNFAIP3; SPOCK1; DDRGK1; SGSM3; AGO3; NAB2; CD74; BAG3; HDAC5; SESN2; FKBP8; MT-RNR2; XIAP; CEMIP; MAPK12; PTGES; HTRA2; MDK; CYP1B1; POLDIP2; NOC2L; MAPK7; RBM14; PML; CITED2; MAPK8; DUSP6; SLC35C1; PLPP1; PLPP3; TSKU; SMARCC2; LEPR; SERPINF1; VPS4A; RITA1; SFRP4; ID2; ID1; CTGF; ATP1A1; CTHRC1; ADAM15; MVP; SPHK1; MAD2L1; CREG1; TMEM259; CLEC16A; JUNB; CORO1B; RRM2; SORBS3; PLPP7; INPPL1; BCL2L12; UNC119; CDK10; PEAR1; KCNK2; MIIP; AURKA; RGS3; NRG1; HOMER3; TRIM27; VASN; CLASP2; RAC1; NR4A1; STAT6; REST; PLAT; GPC1; ABR; CYBA; AP2A1; TNC; LMNA; HMGA1; CTNS; NFIC; TAX1BP3; ARAP1; ACTN4; TIPIN; TOM1L2; PDPK1; ATF6B; KRT18; ATF3 | 4670 | 4652 |
| 0.002113331 | 0.022992946 | GO:1901565 | b | 4 | organonitrogen compound catabolic process | 160; 290; 65992; 1263; 1464; 1889; 2687; 2817; 3084; 3162; 3429; 4085; 4130; 5270; 5371; 5696; 5709; 55627; 6448; 6449; 7041; 7128; 7263; 6790; 8078; 8507; 8659; 8722; 8877; 9146; 9531; 9810; 10312; 10612; 11065; 11313; 112398; 81858; 93343; 51608; 54442; 54461; 26088; 23274; 23464; 23640; 23659; 57214; 253980; 91304; 26002; 26133; 27183; 27352; 27429; 26090; 63891 | BAG3; SPHK1; MAD2L1; KCTD5; PLK3; USP5; PSMD3; DDRGK1; SMPD4; HTRA2; CEMIP; ABHD12; ECE1; MOXD1; TST; SERPINE2; MVB12A; TMEM259; CLEC16A; AURKA; PML; TGFB1I1; IFI27; ENC1; MAP1A; HSPBP1; NRG1; PSMB8; HGS; CTSF; SGTA; GGA1; GPC1; AP2A1; VPS4A; GGT5; GCAT; RNF40; GET4; KCTD13; EGLN2; LYPLA2; ALDH4A1; CSPG4; UBE2C; HMOX1; TNFAIP3; FBXW5; TCIRG1; ANPEP; SGSH; SGSM3; TRIM3; PLA2G15; RNF123; SHARPIN; TRPC4AP | 1284 | 1277 |
| 0.002133747 | 0.022992946 | GO:0060349 | b | 4 | bone morphogenesis | 309; 1291; 3636; 4209; 4665; 5191; 5916; 6424; 7059; 7373 | SFRP4; INPPL1; PEX7; ANXA6; RARG; COL14A1; THBS3; MEF2D; COL6A1; NAB2 | 112 | 112 |
| 0.002136264 | 0.022992946 | GO:0045055 | b | 4 | regulated exocytosis | 29; 81; 221; 290; 823; 976; 1265; 1535; 2621; 3162; 3481; 3614; 3671; 5328; 5413; 5709; 5834; 5878; 5879; 5880; 5978; 6793; 8635; 8804; 8907; 9230; 23406; 9961; 10043; 10312; 10383; 10493; 79792; 84283; 51150; 57153; 124583; 29952; 5170 | MVP; CREG1; ADGRE5; TOM1; STK10; AP1M1; SLC44A2; PSMD3; SEPT5; RNASET2; SDF4; GSDMD; IMPDH1; COTL1; TMEM79; PYGB; RAB11B; CAPN1; DPP7; IGF2; ALDH3B1; RAC2; RAB5C; RAC1; REST; ABR; CYBA; PLAU; VAT1; ACTN4; TUBB4B; ISLR; CNN2; HMOX1; TCIRG1; ANPEP; CANT1; PDPK1; GAS6 | 797 | 794 |
| 0.002212525 | 0.023129344 | GO:0061082 | b | 4 | myeloid leukocyte cytokine production | 972; 2621; 3162; 8482; 23586 | CD74; SEMA7A; DDX58; HMOX1; GAS6 | 31 | 31 |
| 0.002247618 | 0.023129344 | GO:0071705 | b | 4 | nitrogen compound transport | 60; 81; 160; 162; 322; 377; 972; 1263; 1384; 1497; 1535; 2064; 2621; 2852; 3297; 3429; 3434; 3875; 4000; 4130; 4641; 5058; 5127; 5191; 5371; 27315; 5878; 5880; 5905; 5978; 5987; 6192; 6447; 6449; 6563; 6774; 6892; 7128; 7200; 7263; 7538; 8086; 8140; 8425; 8907; 9094; 9146; 9230; 9454; 9531; 9632; 9697; 9961; 10043; 10113; 10312; 10612; 10652; 10758; 10938; 10945; 11070; 11188; 51114; 51172; 55738; 79792; 79971; 113178; 146691; 124565; 29984; 93343; 84885; 79711; 23265; 152579; 54662; 54732; 55144; 26088; 55343; 55357; 23511; 23586; 155066; 57153; 57214; 27183; 27352; 27429; 23381; 29927; 65083 | HSF1; AAAS; PGAP2; ZDHHC12; PLK3; WLS; GPER1; SCFD2; SMG5; NOL6; GSDMD; APBB1; NISCH; ERBB2; SCAMP4; AP1M1; RAB5C; TRIM27; RANGAP1; IFI27; SEC24C; HOMER3; ACTB; KDELR1; PAK1; MAP1A; SLC7A5; ARFGAP1; TST; TMEM115; IFIT1; DDX58; SCG5; RAB11B; TRAF3IP2; TRH; SLC14A1; SGTA; RHOD; PEX7; ZDHHC9; TNFAIP3; TCIRG1; SGSM3; IPO4; GAS6; CD74; BAG3; EHD1; MYO1C; HTRA2; RPS4Y1; STAT3; ARF3; TBC1D2; TMED9; PML; SLC35C1; MVP; LRRC8D; YKT6; GGA1; VPS4A; MVB12A; PREB; TAPBP; ZFP36; TRAM2; TBC1D13; NAGPA; CEMIP; TOM1; UNC119; CDK16; NUP188; SLC38A10; AP1B1; SLC44A2; LTBP4; RAC2; HGS; REST; CYBA; AP2A1; LMNA; CTNS; TRIM3; CRAT; EXOC7; ACTN4; TOM1L2; ATP6V0E2; KRT18; SEC61A1 | 2339 | 2334 |
| 0.002260489 | 0.023129344 | GO:0071260 | b | 4 | cellular response to mechanical stimulus | 476; 1265; 1535; 2022; 4055; 5599; 5879; 9531 | LTBR; BAG3; ENG; CNN2; RAC1; CYBA; ATP1A1; MAPK8 | 77 | 77 |
| 0.002262039 | 0.023129344 | GO:0045184 | b | 4 | establishment of protein localization | 81; 160; 162; 322; 377; 972; 1263; 1384; 2064; 2621; 2852; 3429; 3434; 3875; 4000; 4130; 4641; 5058; 5127; 5191; 5371; 27315; 5599; 5878; 5880; 5905; 5978; 5987; 6192; 6447; 6449; 6774; 7128; 7200; 8086; 8266; 8907; 9094; 9146; 9230; 7013; 9454; 9531; 9632; 9697; 9961; 10043; 10113; 10312; 10612; 10652; 10758; 10938; 10945; 11070; 51114; 51172; 55738; 79792; 79971; 113178; 146691; 29984; 93343; 51608; 84885; 79711; 23265; 152579; 54662; 54732; 26088; 55357; 23511; 23558; 23586; 155066; 57214; 26262; 27183; 27352; 27429; 23381; 29927; 65083 | CD74; BAG3; AAAS; PGAP2; ATP6V0E2; EHD1; TRH; NOL6; TOM1; PLK3; UBL4A; MYO1C; NAGPA; TSPAN17; GPER1; SCFD2; ARFGAP1; HGS; TRAM2; TMEM115; IFIT1; WLS; SMG5; DDX58; RPS4Y1; STAT3; ARF3; UNC119; TBC1D2; ZDHHC12; CDK16; APBB1; TMED9; GSDMD; NUP188; SCG5; RAB11B; HTRA2; PML; TRAF3IP2; AP1B1; RHOD; SGSM3; IFI27; ERBB2; MAP1A; SCAMP4; AP1M1; RAC2; RAB5C; IPO4; TRIM27; RANGAP1; REST; SGTA; MVP; CEMIP; AP2A1; YKT6; SEC24C; WBP2; LMNA; MVB12A; GET4; MAPK8; HOMER3; CRAT; GGA1; VPS4A; KDELR1; EXOC7; PEX7; ACTN4; ZDHHC9; TNFAIP3; PREB; TCIRG1; TERF1; TOM1L2; TBC1D13; TRIM3; GAS6; PAK1; SEC61A1; KRT18 | 2077 | 2073 |
| 0.002442553 | 0.024666773 | GO:0002274 | b | 4 | myeloid leukocyte activation | 29; 221; 290; 823; 972; 976; 1265; 1435; 1535; 3162; 3614; 4055; 5328; 5709; 5834; 5878; 5879; 5880; 6793; 8635; 8804; 8907; 23406; 9961; 10043; 10312; 10383; 10493; 79792; 57153; 124583; 29952; 5170 | CD74; CREG1; ADGRE5; TOM1; STK10; AP1M1; SLC44A2; PSMD3; RNASET2; LTBR; GSDMD; IMPDH1; COTL1; PYGB; CAPN1; DPP7; MVP; ALDH3B1; RAC2; RAB5C; RAC1; PLAU; ABR; CYBA; VAT1; CNN2; TUBB4B; HMOX1; CSF1; TCIRG1; ANPEP; CANT1; PDPK1 | 647 | 645 |
| 0.002531485 | 0.025253102 | GO:0043401 | b | 4 | steroid hormone mediated signaling pathway | 65992; 2852; 3164; 5058; 5916; 7041; 7421; 8611; 9612; 10025; 10432; 112398; 79876; 23558 | PLPP1; RBM14; VDR; RARG; EGLN2; NR4A1; NCOR2; GPER1; UBA5; MED16; DDRGK1; WBP2; TGFB1I1; PAK1 | 195 | 195 |
| 0.002689879 | 0.026271928 | GO:1904844 | b | 4 | response to L-glutamine | 1535; 3297 | HSF1; CYBA | 3 | 3 |
| 0.002697851 | 0.026271928 | GO:0060341 | b | 4 | regulation of cellular localization | 60; 65992; 598; 1263; 1535; 1627; 2064; 2621; 2852; 3162; 3429; 3675; 4000; 4130; 4641; 4926; 5058; 5127; 5371; 5413; 5533; 5599; 5880; 5905; 6455; 7070; 7538; 8086; 9230; 7013; 9531; 10113; 10938; 116372; 83667; 57214; 26119; 27183; 27429; 30846; 30851; 5170 | BCL2L1; BAG3; AAAS; CEMIP; EHD2; EHD1; NUMA1; PLK3; ITGA3; MYO1C; GPER1; SEPT5; DBN1; SH3GL1; HTRA2; LDLRAP1; SESN2; LYPD1; CDK16; RAB11B; PML; MAPK8; IFI27; ERBB2; MAP1A; RAC2; RANGAP1; CYBA; VPS4A; LMNA; THY1; TAX1BP3; HMOX1; PREB; ZFP36; TERF1; DDRGK1; PDPK1; ACTB; PPP3CC; PAK1; GAS6 | 886 | 884 |
| 0.002737822 | 0.026347509 | GO:1901701 | b | 4 | cellular response to oxygen-containing compound | 27; 60; 445; 598; 896; 1291; 1535; 1545; 196883; 2002; 2621; 2852; 3164; 3297; 3371; 3397; 3481; 4313; 4641; 5058; 5127; 5167; 5176; 5179; 5598; 5599; 5905; 5916; 6464; 6774; 6778; 7128; 7421; 7538; 8751; 9076; 9230; 10014; 10312; 10458; 51157; 10280; 29988; 116372; 83667; 23641; 155066; 92140; 27429; 5170 | BCL2L1; HSF1; VDR; HDAC5; SESN2; MYO1C; GPER1; HTRA2; ADAM15; ABL2; ASS1; LDOC1; STAT6; MTDH; CYP1B1; RARG; LYPD1; CDK16; RAB11B; MAPK7; CLDN1; MAPK8; PDPK1; PENK; IGF2; SHC1; NR4A1; RANGAP1; ENPP1; ZNF580; CYBA; SERPINF1; TNC; SIGMAR1; STAT3; ATP6V0E2; ADCY4; SLC2A8; CCND3; TNFAIP3; ID1; TCIRG1; ZFP36; ELK1; COL6A1; BAIAP2; ACTB; MMP2; PAK1; GAS6 | 1104 | 1100 |
| 0.002791099 | 0.026547896 | GO:1901699 | b | 4 | cellular response to nitrogen compound | 60; 445; 598; 896; 1291; 1535; 196883; 2852; 3164; 3297; 3397; 3434; 3481; 4313; 4641; 5058; 5167; 5179; 5905; 6464; 6774; 6778; 10014; 10312; 10458; 29988; 116372; 83667; 23586; 23641; 155066; 5170 | BCL2L1; HSF1; HDAC5; SESN2; MYO1C; GPER1; ASS1; IFIT1; STAT6; DDX58; STAT3; LYPD1; LDOC1; PENK; IGF2; SHC1; NR4A1; RANGAP1; ENPP1; CYBA; PDPK1; ATP6V0E2; ADCY4; SLC2A8; CCND3; ID1; TCIRG1; COL6A1; BAIAP2; ACTB; MMP2; PAK1 | 628 | 625 |
| 0.002825522 | 0.026566403 | GO:0060346 | b | 4 | bone trabecula formation | 4313; 7059; 26585 | GREM1; MMP2; THBS3 | 10 | 10 |
| 0.002889288 | 0.026857249 | GO:0006644 | b | 4 | phospholipid metabolic process | 377; 2064; 2252; 3084; 3628; 3636; 27315; 5833; 5879; 5880; 55627; 8611; 8613; 8733; 8871; 10908; 51477; 79143; 128869; 150763; 347735; 23659; 57153; 26090; 5298; 64748 | PLPP1; PGAP2; PI4KB; GPAT2; SERINC2; SMPD4; MBOAT7; INPPL1; ARF3; ABHD12; SLC44A2; PNPLA6; SYNJ2; NRG1; ERBB2; PLPP3; RAC2; ISYNA1; PLPPR2; RAC1; PCYT2; PIGU; FGF7; PLA2G15; INPP1; GPAA1 | 477 | 476 |
| 0.003005356 | 0.027622261 | GO:0060627 | b | 4 | regulation of vesicle-mediated transport | 27; 29; 81; 160; 1535; 2621; 3162; 5413; 5533; 5878; 5879; 5880; 5978; 6424; 6455; 9094; 9146; 9230; 10113; 10938; 55738; 22848; 23122; 26119; 26585; 27183; 30846; 5170 | EHD2; EHD1; ARFGAP1; SEPT5; ABL2; SH3GL1; UNC119; RAB11B; GREM1; RAC2; CLASP2; RAB5C; RAC1; HGS; REST; AAK1; CYBA; AP2A1; VPS4A; SFRP4; ACTN4; HMOX1; ABR; PREB; PDPK1; PPP3CC; LDLRAP1; GAS6 | 529 | 527 |
| 0.003045388 | 0.027679198 | GO:0060491 | b | 4 | regulation of cell projection assembly | 4651; 5420; 5879; 5880; 6624; 8558; 10435; 10801; 11135; 79647; 116985; 54662; 55357; 27352 | RAC2; ARAP1; AKIRIN1; RAC1; CDC42EP2; CDK10; TBC1D2; PODXL; SEPT9; TBC1D13; SGSM3; CDC42EP1; FSCN1; MYO10 | 200 | 199 |
| 0.003102564 | 0.027888984 | GO:0140014 | b | 4 | mitotic nuclear division | 331; 595; 896; 3481; 4085; 200942; 4926; 23383; 6790; 9055; 9133; 11065; 146691; 54461; 54921; 23122; 27183; 10051 | IGF2; CCNB2; MAD2L1; CLASP2; VPS4A; NUMA1; CCND1; CCND3; TOM1L2; SMC4; FBXW5; XIAP; MAU2; CHTF8; KLHDC8B; PRC1; UBE2C; AURKA | 288 | 288 |
| 0.003301553 | 0.029355113 | GO:0031498 | b | 4 | chromatin disassembly | 3159; 6601; 55201; 83743 | SMARCC2; GRWD1; MAP1S; HMGA1 | 21 | 21 |
| 0.003342378 | 0.029398554 | GO:0035967 | b | 4 | cellular response to topologically incorrect protein | 37; 467; 595; 1388; 2134; 3297; 4000; 6464; 9531; 10113; 55738; 30827 | HSF1; BAG3; ACADVL; SHC1; CCND1; EXTL1; PREB; CXXC1; ARFGAP1; ATF6B; LMNA; ATF3 | 160 | 159 |
| 0.003426955 | 0.029821798 | GO:0036230 | b | 4 | granulocyte activation | 29; 221; 290; 823; 976; 1265; 1535; 3614; 5328; 5709; 5834; 5878; 5879; 6793; 8635; 8804; 8907; 23406; 9961; 10043; 10312; 10383; 10493; 79792; 57153; 124583; 29952 | CREG1; ADGRE5; TOM1; STK10; AP1M1; SLC44A2; PSMD3; RNASET2; GSDMD; IMPDH1; COTL1; PYGB; CAPN1; DPP7; MVP; ALDH3B1; RAB5C; RAC1; PLAU; ABR; CYBA; VAT1; CNN2; TUBB4B; TCIRG1; ANPEP; CANT1 | 509 | 507 |
| 0.00354807 | 0.030352585 | GO:0002495 | b | 4 | antigen processing and presentation of peptide antigen via MHC class II | 160; 162; 972; 3831; 8722; 8907; 9632; 10437; 64837 | CD74; AP1M1; CTSF; AP2A1; IFI30; SEC24C; KLC1; AP1B1; KLC2 | 101 | 101 |
| 0.003562162 | 0.030352585 | GO:0010038 | b | 4 | response to metal ion | 311; 322; 445; 595; 955; 1396; 3162; 3297; 3398; 3726; 3861; 4502; 4512; 5176; 5179; 5536; 5599; 9076; 9536; 353174; 91252 | HSF1; ENTPD6; PTGES; MT2A; MT-CO1; CCND1; ASS1; ZACN; JUNB; HMOX1; APBB1; PPP5C; KRT14; SERPINF1; SLC39A13; PENK; ANXA11; CLDN1; MAPK8; CRIP1; ID2 | 364 | 362 |
| 0.003670238 | 0.030951081 | GO:0097191 | b | 4 | extrinsic apoptotic signaling pathway | 467; 598; 2852; 3162; 3429; 3856; 3875; 4000; 4055; 5371; 5598; 7128; 9531; 27429; 5170 | BCL2L1; LTBR; BAG3; IFI27; HMOX1; TNFAIP3; GPER1; HTRA2; MAPK7; PML; PDPK1; LMNA; KRT8; ATF3; KRT18 | 225 | 225 |
| 0.003735856 | 0.031182965 | GO:0036498 | b | 4 | IRE1-mediated unfolded protein response | 37; 2134; 4000; 6464; 10113; 55738; 30827 | ACADVL; SHC1; EXTL1; PREB; CXXC1; ARFGAP1; LMNA | 67 | 66 |
| 0.003848739 | 0.031800696 | GO:1990874 | b | 4 | vascular smooth muscle cell proliferation | 2852; 3162; 4209; 4313; 5058; 26119 | HMOX1; PAK1; GPER1; MEF2D; MMP2; LDLRAP1 | 50 | 50 |
| 0.004085366 | 0.033418295 | GO:0071773 | b | 4 | cellular response to BMP stimulus | 331; 2022; 3397; 3675; 4681; 4926; 6424; 8200; 23213; 285704; 23770; 26585 | GREM1; FKBP8; SFRP4; NUMA1; NBL1; ITGA3; ENG; ID1; XIAP; GDF5; SULF1; RGMB | 163 | 163 |
| 0.004210125 | 0.034097848 | GO:0030518 | b | 4 | intracellular steroid hormone receptor signaling pathway | 65992; 2852; 5058; 7041; 8611; 9612; 10025; 10432; 112398; 79876; 23558 | PLPP1; EGLN2; WBP2; RBM14; PAK1; GPER1; UBA5; MED16; DDRGK1; TGFB1I1; NCOR2 | 143 | 143 |
| 0.004416412 | 0.035393375 | GO:0031670 | b | 4 | cellular response to nutrient | 1535; 2621; 3162; 3297; 3371; 5179; 7421 | HSF1; VDR; HMOX1; CYBA; TNC; PENK; GAS6 | 68 | 68 |
| 0.004456623 | 0.035393375 | GO:0002685 | b | 4 | regulation of leukocyte migration | 29; 972; 1435; 2621; 3162; 4681; 5879; 5880; 6793; 7070; 51157; 79647; 26585 | CD74; GREM1; RAC2; AKIRIN1; RAC1; NBL1; STK10; HMOX1; CSF1; ZNF580; ABR; THY1; GAS6 | 186 | 186 |
| 0.004773191 | 0.037315435 | GO:0048584 | b | 4 | positive regulation of response to stimulus | 60; 81; 322; 331; 65992; 467; 598; 661; 972; 1435; 1464; 1490; 1535; 1545; 1848; 2022; 2064; 2252; 2621; 2852; 3084; 3159; 3162; 3297; 3340; 3481; 3572; 3575; 3776; 3927; 4055; 4641; 4651; 4926; 5058; 5179; 5371; 5536; 5599; 5606; 5879; 5880; 6275; 6424; 6449; 6464; 6574; 6774; 6778; 7041; 7052; 7070; 7128; 7867; 8200; 23213; 8482; 8558; 8613; 8877; 9500; 10174; 10370; 10432; 10458; 10758; 192669; 51157; 400916; 79156; 84231; 79647; 79971; 147372; 81858; 51246; 116985; 54386; 22848; 23274; 80381; 23558; 23586; 23770; 57153; 91304; 26119; 124583; 26585; 92140; 92799; 27429; 5170 | BCL2L1; HSF1; TGM2; WLS; IL6ST; GPER1; POLR3D; KCNK2; FKBP8; LTBR; NDST1; APBB1; THY1; ERBB2; SHC1; TERF2IP; TGFB1I1; SEMA7A; SFRP4; CSPG4; MAGED1; CSF1; ACTB; PAK1; IL7R; MTDH; MAPKAPK3; DDX58; RAC1; SULF1; TRAF3IP2; IGF2; ENG; SGTA; ZNF580; FGF7; STAT3; PPP5C; CD276; HMOX1; TNFAIP3; SHISA5; DDRGK1; BAIAP2; AGO3; GAS6; CD74; MYO1C; XIAP; CHCHD10; SHKBP1; HTRA2; STAT6; CYP1B1; RBM14; PML; CITED2; MAPK8; DUSP6; PENK; PLPP3; CCBE1; HMGA1; S100A4; LASP1; AKIRIN1; CTGF; LDLRAP1; MYO10; SPHK1; TMEM259; NUMA1; CLEC16A; MAP2K3; SORBS3; PLEKHF1; ARAP1; CDK10; GDF5; NRG1; SLC44A2; GREM1; RAC2; TRAF7; AAK1; CYBA; PDPK1; SLC20A1; ACTN4; ATF3; CANT1; WBP2; SHARPIN | 2371 | 2366 |
| 0.004789879 | 0.037315435 | GO:0040014 | b | 4 | regulation of multicellular organism growth | 10939; 1435; 3297; 3481; 6774; 8200; 27429 | HSF1; IGF2; STAT3; CSF1; GDF5; AFG3L2; HTRA2 | 69 | 69 |
| 0.004998411 | 0.038572643 | GO:0002478 | b | 4 | antigen processing and presentation of exogenous peptide antigen | 160; 162; 972; 3831; 6892; 8722; 8907; 9632; 10437; 64837 | CD74; AP1M1; CTSF; TAPBP; AP2A1; IFI30; SEC24C; KLC1; AP1B1; KLC2 | 126 | 126 |
| 0.005221621 | 0.039650914 | GO:0014070 | b | 4 | response to organic cyclic compound | 60; 445; 65992; 476; 595; 598; 1490; 1535; 1545; 1848; 2002; 2022; 2852; 3164; 3297; 3371; 3397; 3434; 3726; 4192; 5058; 5176; 5179; 5916; 5978; 6774; 7041; 7200; 7421; 7538; 8611; 9076; 9536; 9612; 10014; 10025; 10432; 112398; 79876; 90313; 23558; 23586 | BCL2L1; HSF1; VDR; HDAC5; JUNB; GPER1; ASS1; NCOR2; IFIT1; MDK; DDX58; CYP1B1; RARG; ZFP36; MED16; RBM14; TGFB1I1; DUSP6; PENK; PLPP1; TRH; ENG; UBA5; NR4A1; REST; CYBA; SERPINF1; TNC; CLDN1; STAT3; PTGES; EGLN2; CCND1; TP53I13; ID1; CTGF; ELK1; ATP1A1; DDRGK1; WBP2; ACTB; PAK1 | 920 | 918 |
| 0.005235084 | 0.039650914 | GO:0015031 | b | 4 | protein transport | 81; 160; 162; 322; 377; 972; 1263; 1384; 2064; 2621; 2852; 3429; 3434; 3875; 4000; 4130; 4641; 5058; 5127; 5191; 5371; 27315; 5878; 5880; 5905; 5978; 5987; 6192; 6447; 6449; 6774; 7128; 7200; 8086; 8907; 9094; 9146; 9230; 9454; 9531; 9632; 9697; 9961; 10043; 10113; 10312; 10612; 10652; 10758; 10938; 10945; 11070; 51114; 51172; 55738; 79792; 79971; 113178; 146691; 29984; 93343; 84885; 79711; 23265; 152579; 54662; 54732; 26088; 55357; 23511; 23586; 155066; 57214; 27183; 27352; 27429; 23381; 29927; 65083 | CD74; BAG3; AAAS; PGAP2; ATP6V0E2; EHD1; TRH; NOL6; TOM1; PLK3; AP1M1; MYO1C; NAGPA; GPER1; SCFD2; ARFGAP1; HGS; TMEM115; IFIT1; SMG5; DDX58; RPS4Y1; STAT3; ARF3; UNC119; TBC1D2; ZDHHC12; CDK16; APBB1; TMED9; GSDMD; NUP188; SCG5; RAB11B; HTRA2; PML; TRAF3IP2; AP1B1; SGSM3; IFI27; ERBB2; MAP1A; SCAMP4; WLS; RAC2; RAB5C; IPO4; TRIM27; RANGAP1; REST; SGTA; MVP; CEMIP; AP2A1; YKT6; SEC24C; LMNA; MVB12A; HOMER3; RHOD; CRAT; GGA1; VPS4A; KDELR1; EXOC7; PEX7; ACTN4; ZDHHC9; TNFAIP3; PREB; TCIRG1; TRAM2; TOM1L2; TBC1D13; TRIM3; GAS6; PAK1; SEC61A1; KRT18 | 1967 | 1963 |
| 0.00613369 | 0.046001171 | GO:0034329 | b | 4 | cell junction assembly | 60; 81; 2318; 3861; 4641; 5339; 5879; 6624; 7070; 9076; 29984; 23122; 26585; 92140; 5170 | GREM1; MTDH; CLASP2; ACTN4; RAC1; MYO1C; KRT14; PLEC; PDPK1; CLDN1; ACTB; FLNC; THY1; FSCN1; RHOD | 238 | 238 |
| 0.006185977 | 0.046001171 | GO:0007029 | b | 4 | endoplasmic reticulum organization | 6449; 8266; 9697; 51608; 118813; 29927 | ZFYVE27; UBL4A; SGTA; TRAM2; GET4; SEC61A1 | 55 | 55 |
| 0.0062933 | 0.046377654 | GO:0061430 | b | 4 | bone trabecula morphogenesis | 4313; 7059; 26585 | GREM1; MMP2; THBS3 | 13 | 13 |
| 0.006753546 | 0.049325003 | GO:0032231 | b | 4 | regulation of actin filament bundle assembly | 1490; 3397; 5058; 5879; 10174; 116985; 23122; 55616 | CLASP2; ARAP1; RAC1; ID1; CTGF; SORBS3; PAK1; ASAP3 | 92 | 92 |
| 0.00693789 | 0.050222955 | GO:0000902 | b | 4 | cell morphogenesis | 81; 10939; 322; 396; 1291; 1627; 2064; 2192; 2817; 3084; 3397; 3398; 3575; 4130; 4651; 4681; 5058; 5598; 5879; 5880; 6464; 7070; 7373; 7421; 6790; 8482; 10381; 10435; 10458; 10678; 11135; 11313; 29985; 29984; 116985; 118813; 54870; 55201; 55715; 57175; 25987; 91663; 26585; 53834; 64855 | IL7R; FGFRL1; VDR; COL14A1; LYPLA2; CORO1B; AFG3L2; MYADM; DBN1; FBLN1; B3GNT2; NBL1; APBB1; MAPK7; DOK4; ARHGDIA; NRG1; THY1; QRICH1; GREM1; AURKA; ERBB2; MAP1A; TSKU; RAC2; SHC1; RAC1; CDC42EP2; CDC42EP1; FAM129B; TUBB3; GPC1; MAP1S; SLC39A3; SEMA7A; RHOD; ZFYVE27; ARAP1; ACTN4; ID2; ID1; COL6A1; BAIAP2; PAK1; MYO10 | 1017 | 1016 |
| 0.007310673 | 0.05166738 | GO:0001667 | b | 4 | ameboidal-type cell migration | 309; 1545; 2022; 2252; 3162; 3164; 3397; 3675; 5058; 5176; 5371; 5606; 7763; 8482; 10014; 51157; 51421; 23122; 57175; 26585; 5170 | CORO1B; ITGA3; CYP1B1; CLASP2; ANXA6; ENG; ZNF580; NR4A1; HMOX1; ID1; MAP2K3; PML; ZFAND5; AMOTL2; SERPINF1; PDPK1; FGF7; HDAC5; SEMA7A; PAK1; GREM1 | 388 | 386 |
| 0.007326749 | 0.05166738 | GO:0072331 | b | 4 | signal transduction by p53 class mediator | 972; 1263; 5371; 6878; 6790; 10514; 57060; 83596; 83667; 51246; 26155; 26585; 29965; 64782 | CD74; TAF6; GREM1; SESN2; BCL2L12; PLK3; NOC2L; SHISA5; PCBP4; AURKA; MYBBP1A; PML; CDIP1; AEN | 220 | 220 |
| 0.007326915 | 0.05166738 | GO:0043085 | b | 4 | positive regulation of catalytic activity | 27; 29; 396; 65992; 595; 896; 972; 1435; 1464; 1490; 1535; 1848; 2064; 2192; 2621; 2852; 3084; 3297; 3481; 4665; 5058; 5118; 5371; 5599; 5606; 5905; 5978; 5998; 6300; 6464; 6774; 6793; 7070; 7421; 7867; 22924; 9094; 9322; 9500; 10113; 10435; 10899; 11065; 10985; 400916; 55738; 84231; 116985; 347735; 54662; 55357; 57214; 25894; 26585; 27352; 27429; 30851; 5170; 55616 | CD74; HSF1; VDR; STK10; PML; GPER1; CEMIP; MAPK12; ARFGAP1; CHCHD10; FBLN1; ABL2; IGF2; HTRA2; ASAP3; STAT3; ARAP1; MAPKAPK3; TBC1D2; UNC119; ARHGDIA; PCOLCE; MAPK8; DUSP6; MAPRE3; ERBB2; GREM1; SHC1; TRAF7; CDC42EP2; RGS3; RANGAP1; REST; NRG1; ABR; CYBA; SGSM3; PDPK1; TRIP10; UBE2C; SERINC2; THY1; JTB; MAP2K3; TAX1BP3; PLEKHG4; CSPG4; GCN1; CCND1; MAGED1; CCND3; CSF1; PREB; CTGF; DDRGK1; TBC1D13; GAS6; PAK1; NAB2 | 1413 | 1412 |
| 0.007473489 | 0.052250545 | GO:0071417 | b | 4 | cellular response to organonitrogen compound | 60; 445; 598; 896; 1291; 1535; 196883; 2852; 3164; 3297; 3397; 3481; 4313; 4641; 5058; 5167; 5179; 5905; 6464; 6774; 10014; 10312; 10458; 29988; 83667; 23641; 155066; 5170 | BCL2L1; HSF1; HDAC5; SESN2; MYO1C; GPER1; ASS1; LDOC1; STAT3; PENK; IGF2; SHC1; NR4A1; RANGAP1; ENPP1; CYBA; PDPK1; ATP6V0E2; ADCY4; SLC2A8; CCND3; ID1; TCIRG1; COL6A1; BAIAP2; ACTB; MMP2; PAK1 | 567 | 564 |
| 0.00839021 | 0.058124637 | GO:0001958 | b | 4 | endochondral ossification | 3636; 4209; 4665; 5191 | NAB2; PEX7; INPPL1; MEF2D | 27 | 27 |
| 0.00848312 | 0.058124637 | GO:0048585 | b | 4 | negative regulation of response to stimulus | 29; 467; 598; 972; 1388; 1848; 2064; 2192; 2621; 2817; 2852; 2907; 3162; 3297; 3434; 3489; 3575; 3675; 4000; 4550; 4681; 5055; 5167; 5176; 5270; 5327; 5328; 5598; 5987; 5998; 6424; 6449; 6778; 7041; 7070; 7128; 23213; 8482; 9146; 9454; 9612; 60343; 79102; 84335; 375033; 81858; 114990; 115908; 83596; 83667; 84934; 55343; 23122; 57175; 253980; 25987; 91663; 26155; 26585; 27429; 30851; 5170; 64855 | BCL2L1; CD74; SESN2; FAM3A; ITGA3; FAM129B; MT-RNR2; GPER1; HTRA2; GREM1; MYADM; FBLN1; NCOR2; IFIT1; STAT6; RNF26; BCL2L12; NBL1; NOC2L; PEAR1; SULF1; AKT1S1; SERPINE2; RGS3; TGFB1I1; THY1; DUSP6; SGTA; GRINA; MAPK7; ERBB2; TSKU; VASN; CLASP2; PLAT; IL7R; TRIM27; HGS; PLAU; ENPP1; GPC1; ABR; IGFBP6; PDPK1; RITA1; LMNA; SERPINB2; SEMA7A; HOMER3; KCTD13; CORO1B; TAX1BP3; SFRP4; HSF1; HMOX1; TNFAIP3; SHARPIN; SERPINF1; ATF6B; SLC35C1; CTHRC1; ATF3; GAS6 | 1539 | 1537 |
| 0.008610503 | 0.058124637 | GO:0030050 | b | 4 | vesicle transport along actin filament | 81; 4641 | MYO1C; ACTN4 | 5 | 5 |
| 0.008610503 | 0.058124637 | GO:1990910 | b | 4 | response to hypobaric hypoxia | 3297; 8751 | HSF1; ADAM15 | 5 | 5 |
| 0.008668956 | 0.058124637 | GO:0031532 | b | 4 | actin cytoskeleton reorganization | 27; 396; 2252; 5058; 6464; 10458; 29984; 116985 | SHC1; ARAP1; ARHGDIA; FGF7; BAIAP2; ABL2; PAK1; RHOD | 96 | 96 |
| 0.009452766 | 0.06263867 | GO:0009057 | b | 4 | macromolecule catabolic process | 160; 65992; 823; 1263; 1464; 2817; 3084; 3297; 3429; 4085; 4130; 5270; 5371; 5696; 5709; 5834; 6050; 6192; 6448; 6449; 7041; 7128; 7538; 6790; 8078; 8507; 8635; 8722; 9146; 9531; 9810; 10312; 10612; 11065; 11313; 192669; 112398; 57060; 81858; 93343; 51608; 54442; 54461; 26088; 23274; 23640; 23644; 57214; 253980; 91304; 26133; 27183; 27352; 27429; 23381; 26090; 63891 | HSF1; BAG3; MAD2L1; KCTD5; SHARPIN; PLK3; USP5; PSMD3; DDRGK1; HTRA2; CEMIP; RNH1; EDC4; RNASET2; SERPINE2; ABHD12; SMG5; RPS4Y1; TMEM259; PCBP4; CLEC16A; PYGB; AURKA; CAPN1; PML; TGFB1I1; IFI27; MAP1A; HSPBP1; NRG1; PSMB8; HGS; CTSF; SGTA; GGA1; GPC1; AP2A1; VPS4A; MVB12A; RNF40; GET4; KCTD13; EGLN2; LYPLA2; CSPG4; UBE2C; TNFAIP3; FBXW5; TCIRG1; ZFP36; ENC1; SGSH; SGSM3; TRIM3; RNF123; AGO3; TRPC4AP | 1376 | 1373 |
| 0.00955875 | 0.06263867 | GO:0007628 | b | 4 | adult walking behavior | 1497; 7200; 27429; 26090 | TRH; CTNS; ABHD12; HTRA2 | 28 | 28 |
| 0.009571924 | 0.06263867 | GO:0019471 | b | 4 | 4-hydroxyproline metabolic process | 8659; 112398; 54681 | P4HTM; EGLN2; ALDH4A1 | 15 | 15 |
| 0.009824982 | 0.063784408 | GO:0071229 | b | 4 | cellular response to acid chemical | 27; 445; 598; 1291; 1535; 3297; 3371; 4313; 5176; 5916; 9076; 83667; 27429 | BCL2L1; HSF1; SESN2; RARG; MMP2; CYBA; SERPINF1; TNC; COL6A1; CLDN1; ABL2; ASS1; HTRA2 | 205 | 205 |
