# Supplementary material for: Transcriptome and lipidome profile of human mesenchymal stem cells with reduced senescence and increased trilineage differentiation ability upon drug treatment
Source: Aging (Albany NY). 2021 Mar 26;13(7):9991–10014. doi: 10.18632/aging.202759 (PMC8064146; doi:10.18632/aging.202759)
Supplement: Supplementary Tables 3 and 4 [file aging-13-202759-s004.pdf]

SUPPLEMENTARY TABLES

Supplementary Table 3. Clustering of the lipidomics time series data.

ClusterID	Name
1	Cer(d16:1/16:0)
1	Cer(d18:0/16:0)
1	Cer(d18:0/24:1)
1	Cer(d18:1/16:0)
1	Cer(d18:1/22:0)
1	Cer(d18:1/24:1)
1	Cer(d18:1/24:2)
1	Cer(d18:2/16:0)
1	CerG1(d18:1/16:0)
1	CerG1(d18:1/22:0)
1	CerG1(d18:1/24:0)
1	CerG1(d18:1/24:1)
1	CerG2(d18:1/16:0)
1	ChE(20:5)
1	DG(16:0_18:1)
1	DG(16:0_18:2)
1	DG(18:0_20:4)
1	DG(18:1_18:1);DG(18:0_18:2);DG(16:0_20:2)
1	DG(18:1_18:2)
1	LPC(16:0)
1	LPC(16:0e)
1	LPC(16:0p)
1	LPC(16:1)
1	LPC(17:0)
1	LPC(18:0)
1	LPC(18:0e)
1	LPC(18:0p)
1	LPC(20:3)
1	LPE(16:0p)
1	LPE(18:0p)
1	PC 35:3
1	PC 35:6
1	PC 36:5
1	PC 40:9
1	PC 42:7
1	PC(15:0_18:2)
1	PC(16:0_14:0)
1	PC(16:0_15:0)
1	PC(16:0_17:1)
1	PC(16:0_18:1)
1	PC(16:0_22:6)
1	PC(17:0_20:4)
1	PC(18:0_18:2)
1	PC(18:1_18:2)
1	PC-O 30:1-2
1	PC-O 34:0 (FA 16:0)
1	PC-O 34:1
1	PC-O 34:2
1	PC-O 34:3 (FA 18:2)
1	PC-O 40:7 (FA 22:6)
1	PE(16:0p_20:1)
1	PE(18:0_22:6)

1	PE(18:0p_22:6)
1	PE(18:1p_16:0)
1	PE-O 38:6
1	PE-O 39:5
1	SM 32:1
1	SM 32:2
1	SM 33:1
1	SM 34:0
1	SM 34:4
1	SM 35:1
1	SM 36:0
1	SM 36:1
1	SM 36:2
1	SM 36:3
1	SM 36:4
1	SM 38:1
1	SM 40:4
1	SM 41:2
1	SM 42:3
1	SM 44:2
1	SM 44:3
1	SM(d16:0/16:0)
1	SM(d16:0/18:1)
1	SM(d16:0/18:2)
1	Cer(d18:0/22:0)
1	Cer(d18:0/24:0)
1	Cer(d18:1/24:0)
1	ChE(18:2)
1	ChE(20:4)
1	Co(Q10)
1	TG(10:0_16:0_16:0)
1	TG(16:0_12:0_16:1)
1	TG(16:0_14:0_14:0);TG(16:0_16:0_12:0)
1	TG(16:0_14:0_16:0)
1	TG(16:0_15:0_16:1);TG(16:0_14:0_17:1);TG(14:0_15:0_18:1)
1	TG(16:0_16:0_16:0)
1	TG(16:0_16:0_16:1);TG(14:0_16:0_18:1)
1	TG(16:0_16:0_19:1);TG(16:0_17:0_18:1)
1	TG(16:0_16:1_16:1)
1	TG(16:0_16:1_18:1);TG(16:0_16:0_18:2)
1	TG(16:0_17:1_18:2)
1	TG(16:0_18:1_18:2)
1	TG(16:0_18:1_22:0)
1	TG(16:0_18:1_22:4)
1	TG(16:0_18:1_24:1)
1	TG(16:0_18:1_24:2)
1	TG(17:0_18:1_18:1)
1	TG(17:1_18:1_18:1)
1	TG(18:0_16:0_17:0)
1	TG(18:0_16:0_18:0)
1	TG(18:0_16:0_20:0)
1	TG(18:0_18:0_18:0)
1	TG(18:0_18:0_18:1)
1	TG(18:0_18:1_20:0)
1	TG(18:1_18:1_18:2)
1	TG(18:1_18:1_19:1)

1	TG(18:1_18:1_22:1)
1	TG(18:1_18:1_22:4)
1	TG(18:1_18:2_20:3)
1	TG(18:1_18:2_22:1)
1	TG(18:1_18:2_22:4)
1	TG(18:1_18:2_22:6)
1	TG(18:1_20:4_20:4)
1	TG(20:1_18:1_18:2)
1	TG(22:4_18:2_18:2);TG(22:5_18:1_18:2)
2	FFA 14:1
2	FFA 15:0
2	FFA 16:0
2	FFA 17:0
2	FFA 17:1
2	FFA 18:0
2	FFA 18:1
2	FFA 18:2
2	FFA 19:0
2	FFA 20:0
2	FFA 20:1
2	FFA 20:2
2	FFA 20:3
2	FFA 20:4
2	FFA 20:5
2	FFA 22:0
2	FFA 22:1
2	FFA 22:3
2	FFA 22:4
2	FFA 22:5
2	FFA 22:6
2	FFA 24:0
2	FFA 24:1

The first column contains the cluster ID, and the second column contains the name of lipidomics.

Supplementary Table 4. Clustering of the FFA time series data.

ClusterID	Name
1	FFA 24:1 R_div_control
1	FFA 15:0 R_div_control
1	FFA 18:0 R_div_control
1	FFA 14:1 R_div_control
1	FFA 24:0 R_div_control
1	FFA 24:1 O_div_control
1	FFA 19:0 R_div_control
1	FFA 20:0 R_div_control
1	FFA 20:3 R_div_control
1	FFA 16:0 R_div_control
1	FFA 17:0 R_div_control
1	FFA 17:1 R_div_control
1	FFA 20:1 R_div_control
1	FFA 20:4 R_div_control
1	FFA 22:0 R_div_control
1	FFA 22:6 R_div_control
2	FFA 22:4 V_div_control
2	FFA 20:1 V_div_control
2	FFA 20:2 V_div_control
3	FFA 20:3 V_div_control
3	FFA 18:0 V_div_control
3	FFA 15:0 V_div_control
3	FFA 20:0 V_div_control
3	FFA 17:0 V_div_control
3	FFA 19:0 V_div_control
3	FFA 16:0 V_div_control
3	FFA 20:2 R_div_control
3	FFA 22:1 R_div_control
3	FFA 14:1 V_div_control
3	FFA 24:0 V_div_control
3	FFA 24:1 V_div_control
4	FFA 22:1 V_div_control
4	FFA 18:2 V_div_control
4	FFA 22:6 V_div_control
4	FFA 18:1 V_div_control
4	FFA 22:0 V_div_control
4	FFA 22:3 V_div_control
5	FFA 20:5 V_div_control
5	FFA 22:5 V_div_control
6	FFA 20:5 R_div_control
6	FFA 22:4 O_div_control
6	FFA 17:1 O_div_control
6	FFA 22:1 O_div_control
6	FFA 19:0 O_div_control
6	FFA 16:0 O_div_control
6	FFA 18:1 O_div_control
6	FFA 20:0 O_div_control
6	FFA 22:6 O_div_control
6	FFA 18:1 R_div_control
6	FFA 18:0 O_div_control
6	FFA 20:5 O_div_control
6	FFA 22:5 O_div_control
6	FFA 20:4 O_div_control
6	FFA 20:3 O_div_control

6	FFA 15:0 O_div_control
6	FFA 22:5 R_div_control
6	FFA 18:2 R_div_control
6	FFA 17:0 O_div_control
6	FFA 18:2 O_div_control
6	FFA 20:1 O_div_control
6	FFA 22:0 O_div_control
6	FFA 24:0 O_div_control
7	FFA 20:4 V_div_control
7	FFA 17:1 V_div_control
8	FFA 22:4 R_div_control
8	FFA 18:1 M_div_control
8	FFA 20:1 M_div_control
8	FFA 20:4 M_div_control
8	FFA 22:5 M_div_control
8	FFA 20:5 M_div_control
8	FFA 22:1 M_div_control
8	FFA 15:0 M_div_control
8	FFA 17:0 M_div_control
8	FFA 20:3 M_div_control
8	FFA 17:1 M_div_control
8	FFA 20:2 O_div_control
8	FFA 18:2 M_div_control
8	FFA 22:3 O_div_control
8	FFA 24:0 M_div_control
8	FFA 20:0 M_div_control
8	FFA 14:1 O_div_control
8	FFA 22:3 R_div_control
8	FFA 14:1 M_div_control
8	FFA 16:0 M_div_control
8	FFA 18:0 M_div_control
8	FFA 19:0 M_div_control
8	FFA 20:2 M_div_control
8	FFA 22:0 M_div_control
8	FFA 22:3 M_div_control
8	FFA 22:4 M_div_control
8	FFA 22:6 M_div_control
8	FFA 24:1 M_div_control

Since the FFAs have significant changes compared with control. We combine all the FFAs' time-series fold-changes from the four drug-control pairs and cluster them using TiCoNE (version 1.3). The first column contains the cluster ID, and the second column contains the name of FFA.
