# Supplementary material for: Transcriptome and lipidome profile of human mesenchymal stem cells with reduced senescence and increased trilineage differentiation ability upon drug treatment
Source: Aging (Albany NY). 2021 Mar 26;13(7):9991–10014. doi: 10.18632/aging.202759 (PMC8064146; doi:10.18632/aging.202759)
Supplement: Supplementary References [file aging-13-202759-s005.pdf]

SUPPLEMENTARY REFERENCES

1. Kamburov A, Pentchev K, Galicka H, Wierling C, Lehrach H, Herwig R. ConsensusPathDB: toward a more complete picture of cell biology. Nucleic Acids Res. 2011; 39:D712–7. https://doi.org/10.1093/nar/gkq1156 PMID:21071422

2. Kamburov A, Wierling C, Lehrach H, Herwig R. ConsensusPathDB--a database for integrating human functional interaction networks. Nucleic Acids Res. 2009; 37:D623–628. https://doi.org/10.1093/nar/gkn698 PMID:18940869

3. Wiwie C, Kuznetsova I, Mostafa A, Rauch A, Haakonsson A, Barrio-Hernandez I, Blagoev B, Mandrup S, Schmidt H, Pleschka S, Rottger R, Baumbach J. Time-Resolved Systems Medicine Reveals Viral Infection-Modulating Host Targets. Syst Med (New Rochelle). 2019; 2:1–9. https://doi.org/10.1089/sysm.2018.0013 PMID:31119214
